# The metabolic signature of salt intake: a cross-sectional analysis from the SCAPIS-study

**DOI:** 10.1186/s12986-025-00997-y

**Published:** 2025-09-02

**Authors:** Jonas Wuopio, Lin Yi-Ting, Koen F. Dekkers, Tove Fall, J. Gustav Smith, Anders Larsson, Gunnar Engström, Marju Orho-Melander, Linda S. Johnson, Johan Ärnlöv

**Affiliations:** 1 Department of Neurobiology, Care Sciences and Society, Karolinska Institute, Stockholm, Sweden; 2 Center for clinical research, Uppsala University, Falun, Region Dalarna Sweden; 3 Department of Family Medicine, Kaohsiung Medical University Hospital, Kaohsiung Medical University, Kaohsiung, Taiwan; 4 Molecular Epidemiology, Department of Medical Sciences, Uppsala University, Uppsala, Sweden; 5 The Wallenberg Laboratory, Department of Molecular and Clinical Medicine, Institute of Medicine, Department of Cardiology, Gothenburg University, Sahlgrenska University Hospital, Gothenburg, Sweden; 6 Department of Cardiology, Clinical Sciences, Lund University and Skåne University Hospital, Lund, Sweden; 7 Wallenberg Center for Molecular Medicine and Lund University Diabetes Center, Lund University, Lund, Sweden; 8 Department of Clinical Sciences in Malmö, Lund University, Malmö, Sweden; 9 Department of Medical Sciences, Clinical Chemistry, Uppsala University, Uppsala, Sweden; 10 Population Health Research Institute, McMaster University, Hamilton, Canada; 11 School of Health and Social Studies, Dalarna University, Falun, Sweden

**Keywords:** Sodium, Salt, Metabolomics, Metabolism, Prevention, Cardiovascular disease

## Abstract

**Background:**

Untargeted metabolomic analysis provides novel insights into the relationship between sodium intake and cardiometabolic risk. This study examined cross-sectional associations between estimated sodium intake and plasma metabolite profiles in a large Swedish cohort.

**Methods:**

This cross-sectional analysis was conducted in the in the SCAPIS cohort (mean age 50–64 years, *n* = 8,957). Sodium intake was estimated using the Kawasaki formula (est24hNa) from urine samples. Plasma metabolites were measured using ultrahigh performance liquid chromatography-tandem mass spectrometry (UPLC-MS/MS) (Metabolon Inc^®^), identifying 713 metabolites grouped into eight biochemical classes (CC). Principal component analysis (PCA) was conducted for each CC, and the first principal component (PC1) was used as the response variable, with est24hNa, age, sex, and cardiovascular risk factors as predictors in restricted cubic spline models. ANOVA and pathway enrichment analyses were performed to explore associations.

**Results:**

Est24hNa was significantly associated with the lipid and energy CC. Lower est24hNa was linked to higher concentrations of free fatty acids and citric acid cycle intermediates, suggesting enhanced beta-oxidation. Bonferroni-adjusted analyses revealed 231 metabolites significantly associated with est24hNa, with 2 S,3R-dihydroxybutyrate (β = -0.13, *p* = 2.28 × 10^− 37^) showing the strongest association. Lipid subgroups including phosphatidylcholines, lysophospholipids, bile acids, and plasmalogens were positively associated with est24hNa. Pathway enrichment suggested links to branched-chain amino acid metabolism and biosynthesis of unsaturated fatty acids.

**Conclusions:**

Lower salt intake was associated with a metabolic profile indicative of increased beta-oxidation, while higher salt intake was linked to lipid species previously implicated in atherosclerosis. These findings highlight potential metabolic pathways through which salt intake may influence cardiovascular health and merit further evaluation in longitudinal studies.

**Supplementary Information:**

The online version contains supplementary material available at 10.1186/s12986-025-00997-y.

## Introduction

Excessive salt consumption is widely recognized as a risk factor for hypertension [1–4] and cardiovascular disease (CVD) [5, 6]. Emerging evidence suggests that excessive salt intake not only elevates blood pressure but also induces metabolic changes that could predispose to CVD [7, 8]. Untargeted analysis of the circulating metabolome offers a powerful tool to investigate such systemic effects by capturing comprehensive snapshots of metabolic profiles in relation to environmental exposures like diet. However, existing studies linking salt intake to the metabolome have been limited by small sample sizes, short-term intervention designs, or targeted analytical platforms [9–15]. As a result, there is a lack of large-scale, population-based studies using untargeted metabolomics to examine habitual salt intake in free-living humans. This represents a critical gap in understanding the molecular pathways through which salt may influence cardiometabolic risk.

We have previously shown that increased salt intake is associated with a higher prevalence of manifest sub-clinical coronary and carotid atherosclerosis [16] in a sub-study of the Swedish CardioPulmonary bioImage Study (SCAPIS). In the current study, we leverage untargeted plasma metabolomics in over 8,000 individuals from SCAPIS to investigate the metabolic patterns associated with estimated salt intake. This may offer new insights into mechanistic links between sodium exposure and cardiovascular disease risk.

## Method

### Study design and population

We used cross-sectional data from SCAPIS to investigate the metabolic patterns in plasma associated with increasing levels of salt intake. SCAPIS was designed to enhance the understanding of cardiovascular- and pulmonary diseases through detailed imaging and extensive biomarker profiling; details of this cohort have been described elsewhere [16, 17]. In short, 30,154 participants between the age 50–64 were recruited from the general population in six Swedish regions. They underwent detailed phenotype investigations to provide a rich dataset encompassing a wide range of clinical, imaging and biochemical parameters.

**Fig. 1:**
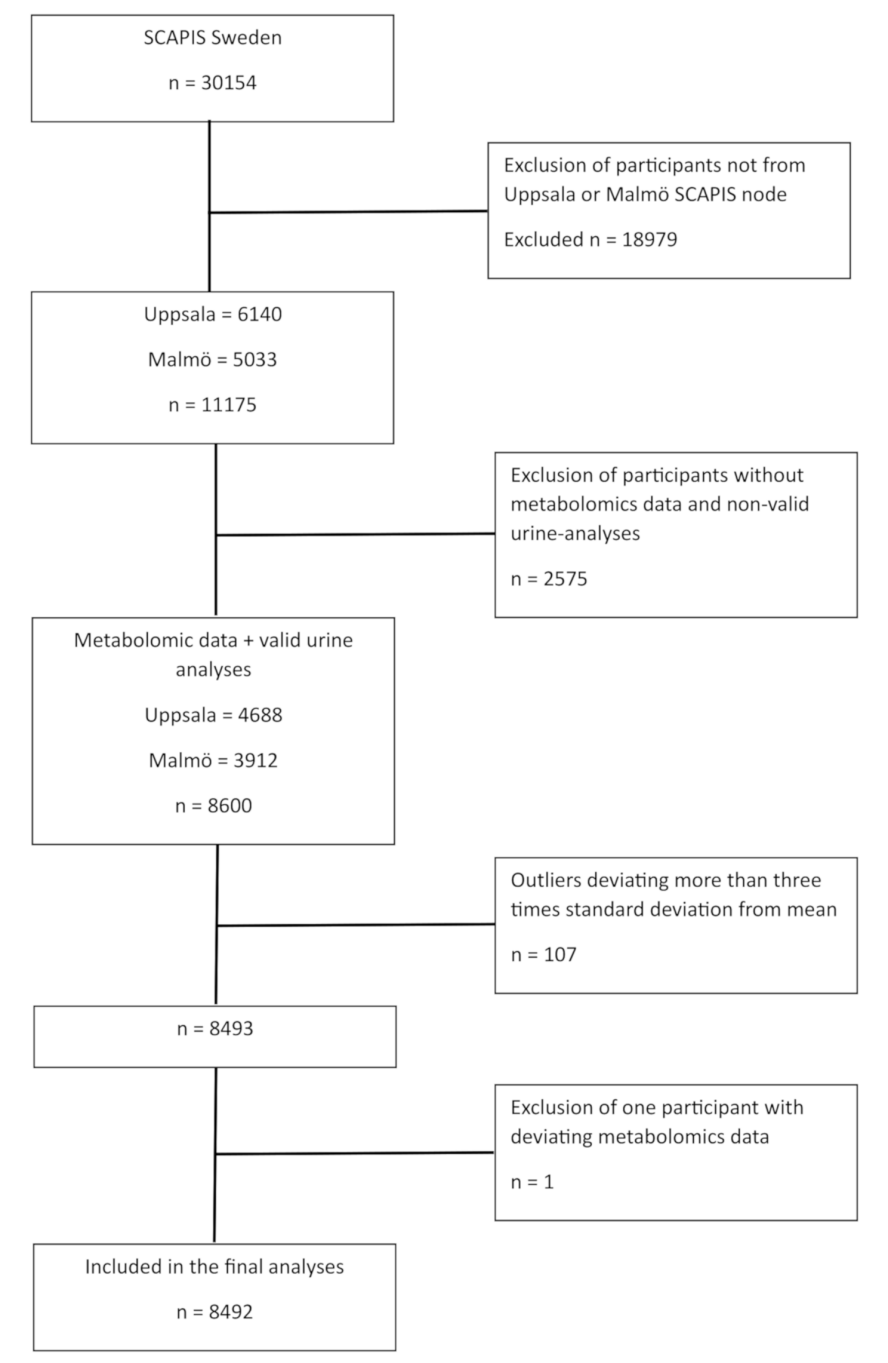
Flow chart of included participants

For this study, we used the participants with metabolomics data and valid analysis for urinary-sodium and urinary-creatinine (second morning void) from the Uppsala region (*n* = 4688) and Malmö region (*n* = 3912) (flow chart Fig. 1). We used the Kawasaki formula [18] to estimate 24-hour sodium excretion (est24hNa) as a proxy for salt intake. In the largest validation study against 24-hour urine collections, the Kawasaki formula showed an intraclass coefficient (ICC) of 0.71.

The Kawasaki formula:

$${\rm est24hNa}\, ({\rm mg/day}) = 22.99 \times 16.3 \,{\rm x}\:\sqrt{{X}_{Na}}$$

Where.

$${\rm X}_{\rm Na} =\:\frac{{SMU}_{Na}}{{SMU}_{Cr}}\:x\:(PreCr-excretion)\:$$

SMU_Na_ (mmol/L).

SMU_Cr_ (mg/L).

Male: PreCr-excretion (mg/day) = -12.63 x Age + 15.12 x Weight (kg) + 7.39 x Height (cm) – 79.9.

Female: PreCr-excretion (mg/day) = -4.72 x Age + 8.58 x Weight (kg) + 5.09 x Height (cm) – 74.5.

We excluded outliers deviating more than three times SD from the mean (*n* = 107) in est24hNa, leaving 8493 participants as the present study sample. The distribution for est24hNa is presented in additional file 1. The Kawasaki formula provides an estimate of raw 24-hour sodium excretion and does not adjust for energy intake. Therefore, to account for potential confounding by total energy intake, all downstream statistical models (including PCA and regression analyses) were adjusted for energy intake (kcal/day), calculated from food frequency questionnaires by a dietician.

### Metabolomics

Blood samples were collected at the time of investigation and frozen in -80 degrees Celsius until analysis. Ultrahigh performance liquid chromatography-tandem mass spectroscopy (UPLC-MS/MS) was conducted by Metabolon (Durham, NC, USA) to analyze untargeted plasma metabolites in three delivery batches from SCAPIS Uppsala-Malmö (Uppsala 1 and 2 [*n* = 4979] and Malmö [*n* = 3978]) as described previously [19]. After a quality-control step, the result generated a dataset delivered to SCAPIS with 1308 metabolites all together subclassified into 10 separate chemical classes (CC) based on their biochemical class.

The metabolites belonging to the CC “unknown metabolites” (m = 263) and “xenobiotics” (m = 210) were excluded, the latter since exogenous substances do not reflect the mechanism of interest in this study. We also chose to omit metabolites with more than 20% missing values (m = 122) leaving 713 metabolites included in the study. Missing data due to measurements under the detection level were imputed with the minimum level above detection limit by Metabolon. The remaining missing values in the dataset are due to factors such as “sample loss.”

### Statistical analysis

Descriptive statistics were calculated for the included participants. Continuous variables were summarized using mean and standard deviation, and categorical variables as counts and percentages. Differences between groups were assessed using Student’s t-test for continuous variables and chi-square test for categorical variables.

The entire dataset, including both metabolites and covariates, was imputed using multiple imputation with 10 imputed datasets via the `mice´ package in R (Missing values count for metabolites and covariates are presented in additional file 1). The metabolite data were standardized (mean = 0, standard deviation = 1) and then log-transformed to improve the normality of the distribution. Initially, we divided the sample into “high” and “low” est24hNa using the median value as cut-off. We performed a principal component analysis (PCA) on the whole metabolite dataset and visualized potential clustering between high and low est24hNa-groups using a PCA-plot. We noted that one participant deviated significantly from the others due to high divergence in several metabolites and was therefore considered as an outlier and removed from the analysis (*n* = 8492).

To gain a broader perspective on metabolic changes related to salt intake, and given that metabolic correlation is largely restricted to within chemical classes [19], we divided the dataset into the eight separate CC: s (excluding xenobiotics and unknown metabolites) and performed PCA on every CC.

PCA was used to reduce dimensionality and summarize correlated metabolites into principal components, which served as composite outcomes in models with estimated 24-hour sodium excretion as the exposure. This approach minimizes noise and multiple testing, and captures broader metabolic patterns relevant to salt intake.

For the whole metabolome and for each CC, we applied PCA separately within each of the 10 imputed datasets, using a custom dtrans() function in the fit.mult.impute() workflow (R, rms package). Metabolite data were log-transformed and standardized prior to PCA. The first principal component (PC1) from each imputed dataset was used as the response variable in regression models adjusted for relevant covariates. Regression models were fitted separately for each imputation, and Rubin’s rules were used to pool the regression coefficients and standard errors across imputations.

We selected PC1 as the best representation of overall metabolic variation and used it as the response variable. A model with restricted cubic splines was then fitted (via fit.mult.impute() from the rms package in R) with est24hNa as a continuous predictor variable (knots at the 5th, 35th, 65th, and 95th percentiles). We employed this approach on the whole metabolome as well as in the separate CCs. ANOVA was used for the statistical analysis of the model.

All models were adjusted for following covariates: age, sex, delivery batch (Uppsala 1, Uppsala 2, or Malmö), systolic and diastolic blood pressure (measured as the average of two readings taken after a 5-minute supine rest at inclusion), body mass index (BMI - calculated as weight/length²), diabetes mellitus (doctor-diagnosed or self-reported), smoking status (current, former, or never smoker), estimated glomerular filtration rate (eGFR, calculated using the Lund-Malmö formula [20]), total plasma cholesterol, total energy intake (calculated by a dietician from food frequency questionnaires), and the use of medications for hypertension or hyperlipidemia (derived from questionnaires). Descriptive analysis of the model showed no indication of multicollinearity, as Variance Inflation Factors (VIF) were close to one for all covariates.

We additionally conducted multiple linear regression analyses for each individual metabolite in the entire dataset, with est24hNa as the continuous predictor and using the same covariates as previously, ranking them based on the *p*-values (using the with(), and pool() function from `mice´ package in R which uses Rubin´s rule [21] for pooling of the results). We divided the metabolites into positively and negatively associated to est24hNa and used Metaboanalyst 6.0 to perform two enrichment analyses of the two separated significant metabolite-set using the KEGG-dataset as reference. Bonferroni-corrected *p*-values were applied in all analyses to correct for multiple testing analysis except for the enrichment analysis where False Discovery Rate (FDR) are used.

### Secondary analyses

To study the associated effect of meat intake and differences in intake in macronutrients on the metabolite patterns we did a sub-analysis where all analyses were adjusted for total intake of red meat (processed and non-processed), poultry, fat, protein and carbohydrates in g per day derived from questionnaires.

All analyses were performed in R version 4.4.0.

## Results

Descriptive statistics of the included participants are presented in Table 1.

**Table 1:** Descriptive statistics of the included participants (mean values, standard deviations and percentages). Student’s t test with corresponding *p*-value for difference (*indicates chi-square test)

	Low est24hNa (*n* = 4246)		High est24hNa (*n* = 4246)	*p*-value
Est24hNa (mg)	2263 (608)		4224 (858)	< 0.001
Systolic blood pressure (mmHg)	122 (16)		126 (16)	< 0.001
Diastolic blood pressure (mmHg)	75 (10)		77 (10)	< 0.001
Sex (% Women)	64		40	< 0.001*
Age (years)	57 (4.4)		57 (4.3)	0.77
BMI, kg/m2	26 (4.2)		28 (4.4)	< 0.001
Smoking (%) Current Never Former				0.23*
	13		12	
	52		53	
	35		35	
Diabetes Mellitus (%)	4.5		5.6	
Cholesterol (mmol/l)	5.6 (1.1)		5.5 (1.1)	< 0.001
eGFR (ml/min/1.73 m^2^)	77 (10)		79 (10)	< 0.001
Hypertension medication (%)	18		20	0.024*
Lipid medication (%)	7		9	0.004*
Energy intake (kJ)	7144 (2849)		7333 (3082)	0.004
Red meat intake (g/day)	65 (43)		78 (50)	< 0.001
Poultry intake (g/day)	22 (18)		24 (19)	< 0.001
Fat intake (g/day)	70 (39)		71 (37)	0.75
Protein intake (g/day)	69 (29)		70 (29)	0.058
Carbohydrates intake (g/day)	185 (104)		190 (103)	0.027

Participants with a higher est24hNa had higher systolic- and diastolic blood pressure and BMI, higher prevalence of diabetes mellitus and a higher proportion of participants with medication for hypertension and/or hyperlipidemia (splines for blood pressure, BMI and cholesterol are presented in additional file 1). Additionally, these participants also consumed significantly more energy and meat.

**Fig. 2:**
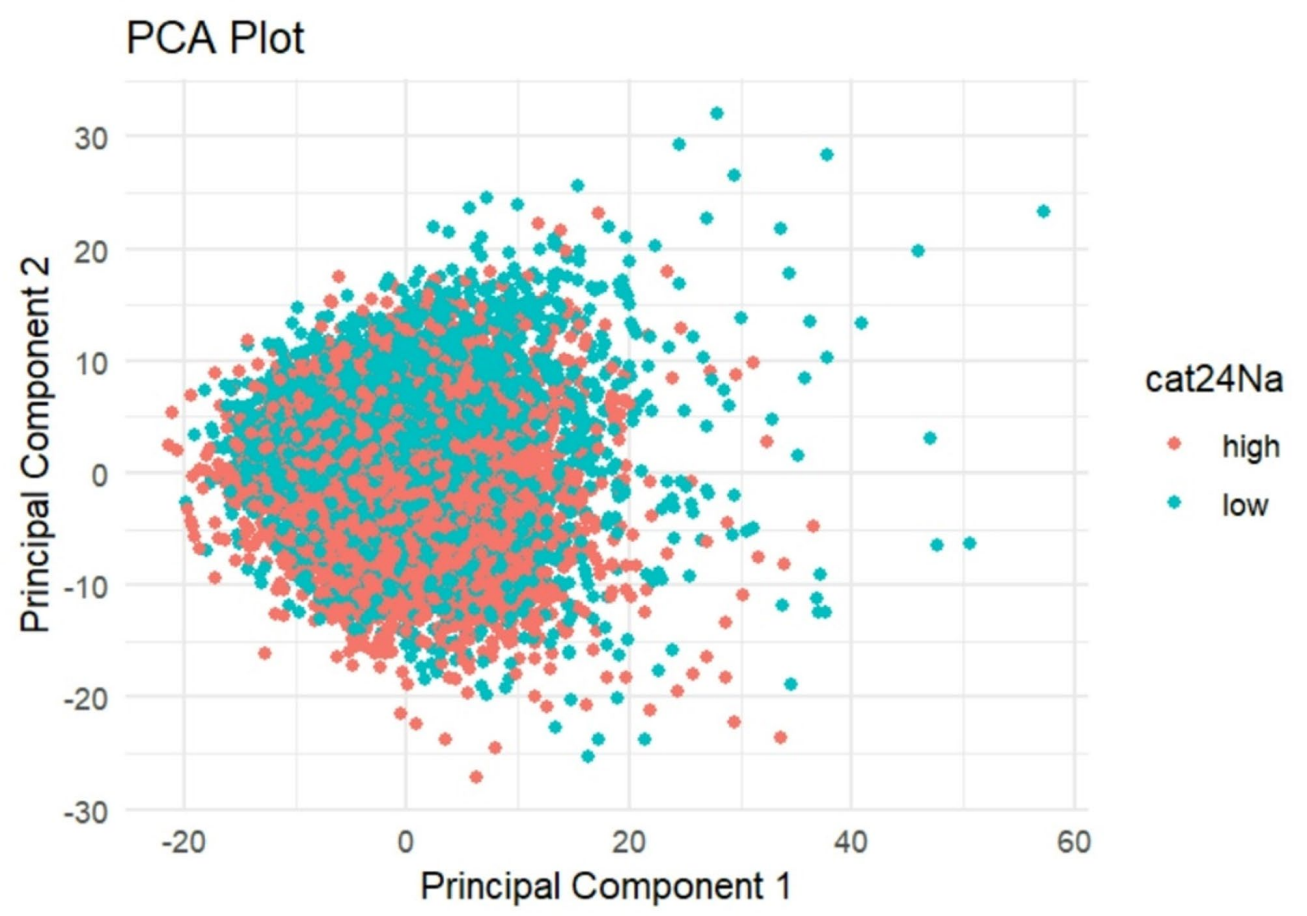
PCA-plot of the whole metabolome separated into high and low est24hNa

There was a significant association between PC1 and est24hNa in the analysis of the whole metabolome (*p* < 0.0001). The PCA-plot, stratified by high and low est24hNa intake, showed considerable overlap between the groups, although a subtle shift in the distribution was visible (Fig. 2). This suggests that while salt intake does not dominate the global variance in the metabolome, it may still influence specific metabolic patterns, as supported by subsequent regression analyses.

Three out of nine CCs (energy, lipid, and partially characterized molecules (PCM)) showed a statistically significant (p_bonf_ <0.05) association to est24hNa where energy and lipid CC stood out as highly significant (both p_bonf_ <0.0001, Table 2).

**Table 2:** Association between first principal components of chemical classes and est24hNa with Bonferroni-corrected *p*-values and p for non-linearity (ANOVA)

Chemical Class	*p*_bonf_	*p*_non−linearity_
Amino acids	1	0.063
Carbohydrates	0.58	0.035
Cofactors and vitamins	1	0.98
Energy	< 0.001	< 0.001
Lipids	< 0.001	< 0.001
Nucleotides	0.26	0.014
Peptides	1	0.11
Partially characterized molecules	0.006	0.48

All analyses adjusted for: Age, sex, delivery batch, systolic- and diastolic blood pressure, BMI, Diabetes Mellitus, smoking, Cholesterol, eGFR, total energy intake, medication for hypertension and hyperlipidemia

Splines for the analyses were presented in additional file 1. Splines for both energy and lipid CCs showed a negative slope down to approximately 4000 mg est24hNa where the curve leveled off (both p for non-linearity < 0.001). For PCM the spline showed a linear positive slope (*p* = 0.00074, p for non-linearity = 0.47).

Seven out of nine metabolites in the energy CC had a significant association to est24hNa, with five out of these seven metabolites showing an inverse association (Table 3). The three top ranked metabolites were citraconate/glutaconate, aconitate and malate.

**Table 3:** Energy metabolites ranking and Bonferroni-corrected *p*-values (est24hNa in g)

	Metabolite	HMDB	β	*p*_bonf_
1.	citraconate/glutaconate	HMDB0000634	0.070	< 0.001
2.	aconitate [cis or trans]	HMDB0000958	-0.064	< 0.001
3.	malate	HMDB0031518	-0.060	< 0.001
4.	fumarate	HMDB0000134	-0.047	< 0.001
5.	phosphate	HMDB0001429	-0.041	< 0.001
6.	citrate	HMDB0000094	-0.038	< 0.001
7.	succinylcarnitine (C4-DC)	HMDB0061717	0.027	0.021
8.	alpha-ketoglutarate	HMDB0000208	-0.020	0.20
9.	succinate	HMDB0000254	-0.017	0.36

All analyses adjusted for: Age, sex, delivery batch, systolic- and diastolic blood pressure, BMI, Diabetes Mellitus, smoking, Cholesterol, eGFR, total energy intake, medication for hypertension and hyperlipidemia

For the lipid CC, 149 out of 375 (40%) metabolites had a significant association to est24hNa (top ten presented in Table 4, full table of significant lipid metabolites in additional file 2). Approximately 84% of these metabolites had an inverse association. Metabolites with predominantly positive estimates belonged to the sub pathways lysophospholipids, phosphatidylcholine, plasmalogen, lysoplasmalogens and primary-, and secondary bile acid metabolism. The top three ranked metabolites from the lipid CC were 2 S,3R-dihydroxybutyrate, palmitate and oleate/vaccinate (Table 4).

**Table 4:** The 10 highest ranked lipid metabolites with Bonferroni-corrected *p*-values (est24hNa in g)

	Metabolite	HMDB	β	*p*_bonf_
1.	2 S,3R-dihydroxybutyrate	HMDB0002453	-0.11	< 0.001
2.	palmitate (16:0)	HMDB0000220	-0.10	< 0.001
3.	oleate/vaccenate (18:1)	HMDB0003231	-0.099	< 0.001
4.	acetylcarnitine (C2)	HMDB0000201	-0.10	< 0.001
5.	linoleate (18:2n6)	HMDB0006270	-0.093	< 0.001
6.	margarate (17:0)	HMDB0002259	-0.091	< 0.001
7.	dihomo-linoleate (20:2n6)	HMDB0005060	-0.092	< 0.001
8.	10-heptadecenoate (17:1n7)	HMDB0060038	-0.090	< 0.001
9.	eicosenoate (20:1)	HMDB0002231	-0.092	< 0.001
10.	10-nonadecenoate (19:1n9)	HMDB0013622	-0.089	< 0.001

All analyses adjusted for: Age, sex, delivery batch, systolic- and diastolic blood pressure, BMI, Diabetes Mellitus, smoking, Cholesterol, eGFR, total energy intake, medication for hypertension and hyperlipidemia

In total, 231 out of all 713 metabolites (32%) had a significant association to est24hNa. The top ranked metabolite was 2 S,3R-dihydroxybutyrate (β = -0.13, *p* = 2.28 × 10^− 37^) (Additional file 3).

**Fig. 3:**
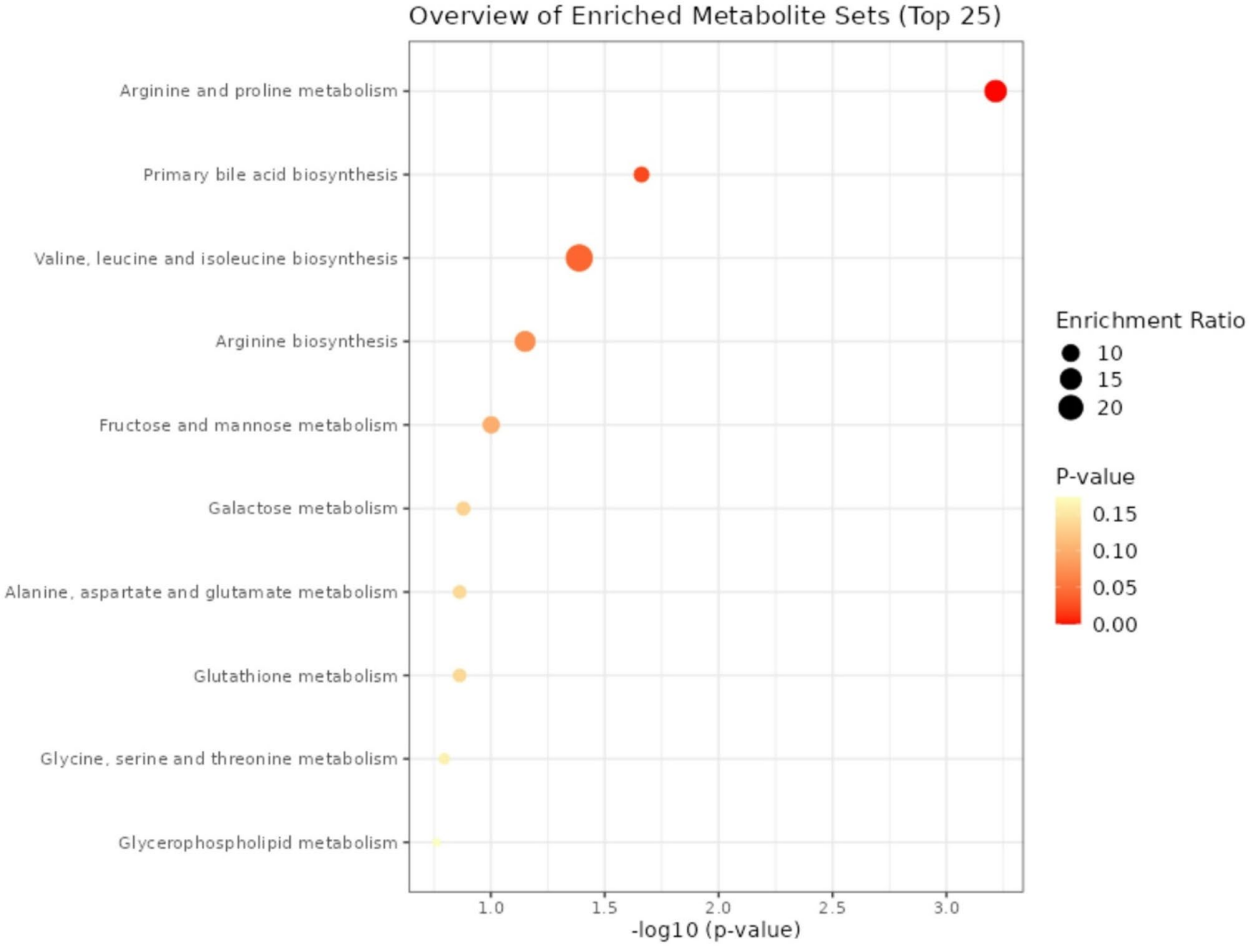
Enrichment analysis linking metabolic pathways for metabolites with an inverse association to est24hNa (Metaboanalyst 6.0)

When using the KEGG-database as reference, enrichment analysis linked lower est24hNa with upregulation of the valine, leucine and isoleucine biosynthesis pathway (FDR = 5.3 × 10^− 7^), the pathway for biosynthesis of unsaturated fatty acids (FDR = 1.1 × 10^− 3^) and the pathway for valine, leucine and isoleucine degradation (FDR = 1.1 × 10^− 2^) (Fig. 3). For metabolites with positive association to est24hNa, enrichment analysis showed association to arginine and proline metabolism (FDR = 4.9 × 10^− 2^).

Adjusting for meat intake and intake of macronutrients did not change the results significantly in any of the above analyses (additional file 4).

## Discussion

### Main findings

The primary finding of this cross-sectional metabolomic study was that lower estimated salt intake was predominantly associated with higher levels of metabolites involved in energy- and lipid metabolism. For energy-related metabolites, most were intermediates of the citric acid (Tricarboxylic acid - TCA) cycle, all of which were significantly elevated in participants with low salt intake. This increase in TCA intermediates, a process known as “anaplerosis”, replenishes the TCA cycle to support sustained energy production [22].

Additionally, we observed elevated levels of free fatty acids among participants with low salt intake. This indicates ongoing lipolysis and beta-oxidation as alternative energy sources. This hypothesis is further supported by higher levels of the ketone body 3-hydroxybutyrate, a product of beta-oxidation, and findings of higher concentrations of acyl carnitines which are upregulated to transport fatty acids in to the mitochondria for energy production [23]. The association to Branched Chained Amino Acids (BCAAs) in enrichment analysis also supports fatty acid oxidation since it has been shown that increased use of fat as an energy source indeed increases BCAA concentrations in blood [24]. This combination of anaplerosis and beta-oxidation typically occurs when there is a high energy demand or limited carbohydrate availability, such as during endurance exercise [25], fasting, or starvation [26]. The metabolomic pattern of low salt intake in this study aligns closely to the metabolomic findings of human starvation [27]. This metabolic state among participants with low salt intake seems unlikely to be attributed to calorie intake differences, as the results were adjusted for caloric intake, with only a minor difference of a few hundred kcal between high- and low-salt participants. There were no significant differences in intake of macronutrients between participants with high or low-salt intake, hence excluding this as an explanation.

While differences in micronutrients affecting metabolism or other residual confounding cannot be entirely excluded, one possible interpretation is that higher salt intake may be associated with a metabolic profile favoring lipogenesis and energy conservation. This interpretation is supported by the higher BMI and body fat observed in high-salt participants, both in this study and in previous research [7, 28, 29]. Although we did not observe a classic circulating metabolite pattern for lipogenesis among participants with a high intake of salt, we noted higher levels of phosphatidylcolines and lysophospholipids which could reflect adipogenic processes through membrane remodeling. In contrast, participants with low salt intake displayed a pattern indicative of enhanced β-oxidation and catabolic metabolism, suggesting a metabolic divergence. Another possibility is that adipogenesis occurs within the adipose tissues and is not fully reflected in peripheral blood. However, given the cross-sectional design, reverse causation is also plausible — for instance, metabolic dysfunction may influence salt preference. Such association may reflect a metabolic state linked to increased risk of metabolic syndrome and related cardiovascular outcomes.

Other important findings related to CVD include the positive association of several phosphatidylcholines (PCs), lysophosphatidylcholines (LPCs) – a subclass of lysophospholipids – and bile acid metabolites with salt intake. PCs, especially highly saturated or oxidized, have been linked to atherosclerosis both in plasma and within the atherosclerotic plaque [30, 31]. Although speculative, we observed a predominance of omega-6–containing phosphatidylcholines among the unsaturated PCs, suggesting a skewed omega-6 to omega-3 ratio — a pattern that has been associated with adverse cardiovascular health [32]. Notably, the only PC inversely associated with salt intake was 1-palmitoyl-2-docosahexaenoyl-GPC, which contains DHA — an omega-3 fatty acid well-documented for its cardioprotective properties [33]. The LPCs identified are all considered pro-inflammatory, capable of activating the endothelium, recruiting monocytes, and contributing to foam cell and plaque formation [34]. The bile acid profile observed aligns with patterns previously associated with atherosclerosis [35], including potential impairments in sulfation and clearance of toxic intermediates [36]. These findings complement the apparently higher metabolic flexibility observed among participants with low salt intake, a pattern generally associated with favorable cardiovascular health [37]. Together, the results provide a theoretical basis for how high salt intake may negatively impact the cardiovascular system beyond blood pressure, through adverse effects on lipid metabolism and endothelial function.

## Biological explanations

Salt has been shown to affect metabolism in several ways. In vitro, a high salt load increased the expression of adipogenic/lipogenic genes, while downregulating genes involved in lipolysis, resulting in fat accumulation [38]. In a high salt fed rat model, chronic high salt intake stimulated the polyol pathway causing increased glucose-to-fructose conversion which led to the metabolic syndrome [8, 39]. In the same study, it was shown that this led to leptin resistance. That high salt intake can affect leptin levels, leptin resistance and adipose hypertrophy has been confirmed in two other studies in rat [40, 41]. In humans, a seven-day high dietary salt intake stimulated ghrelin, a gastric derived peptide that has the ability to stimulate appetite to prevent undernutrition [8, 42]. The gut derived incretin Glucagon Like Peptide-1 (GLP-1), which has the ability to stimulate satiety, reduce food intake and has positive effects on cardiovascular health [43], has been shown to increase with low salt intake in salt-sensitive individuals [8, 44].

The highest-ranked metabolite was 2 S,3R-dihydroxybutyrate (also known as 4-deoxythreonic acid), which showed a negative linear association with salt intake. Little is known about this secondary metabolite which is a metabolite of the metabolism of L-threonine, though decreased urinary levels have been associated to higher intake of ultra processed foods [35] which is a theoretically appealing link to high salt intake.

### Comparisons with previous studies

Compared to previous studies, our work adds new insights by examining metabolic responses to habitual sodium intake in a large and well-characterized cohort. The availability of detailed phenotyping, including measures of subclinical and clinical atherosclerosis, allows us to explore possible links between sodium-related metabolic patterns and vascular health in greater depth than has typically been done.

There are a few previous studies addressing salt intake and metabolomics using short term interventions [9–14]. The findings from these studies have been varied, likely due to differences in the study design, specific metabolites analyzed, and the baseline characteristics of the participants. In a previous cross-sectional study by R. Hamaya et al. [15], salt intake was measured using 24-hour urinary collections. They identified 38 metabolites that were significantly associated with salt intake. The metabolite with the strongest positive association was “piperine”, a black pepper residue, while “proline-betaine”, a marker for citrus intake, showed the strongest negative association. We conducted an additional analysis focusing on these xenobiotic metabolites, and our results were largely consistent (additional file 1). Additionally, there were similar positive associations with plasmalogens and phosphatidylcholines, as well as the involvement of BCAAs in the enrichment analysis. The main difference in our study was the higher proportion of significantly associated metabolites, which is likely due to our larger metabolite set and a cohort that is approximately nine times larger, providing greater statistical power.

### Clinical implication

The observed association between lower est24hNa and increased concentrations of free fatty acids and citric acid cycle intermediates may reflect a more flexible and catabolically active metabolic state. While this may indicate favorable metabolic health in general populations, it could pose challenges in vulnerable groups such as older adults, patients with chronic kidney disease, or those undergoing dialysis, where energy balance and muscle preservation are critical. Importantly, the accompanying lipidomic profile associated with higher salt intake — characterized by pro-atherogenic phosphatidylcholines, pro-inflammatory lysophospholipids, and bile acids linked to atherosclerosis — points toward a metabolomic signature potentially associated with increased cardiovascular risk. Although the cross-sectional design limits causal inference, these findings underscore the importance of further investigating the cardiometabolic consequences of dietary salt intake in both the general and high-risk populations.

### Strengths and limitations

The strengths of the study are the large-scale analyses of 713 plasma metabolites in a large sample with detailed characterization of the study participants from the contemporary cohort SCAPIS. Measurement of metabolites in blood is complex, theoretically yielding results depending on several aspects in the handling of the blood sample (for example: batch number, platform, time to freezer). Multiple steps in the quality-control of the data by Metabolon and after data arrival at SCAPIS have been carried out to minimize these effects. Normalization and adjustment for delivery batch also deal with this problem. The high degree of collinearity among the metabolites, representing the same sub pathways, makes it hard to identify specific metabolites of importance.

The Kawasaki formula has been validated against 24-hour urine collections [18, 45] but also criticized for potentially causing biased results with J-formed associations, as some claim the constituent variables (like weight) correlate with the outcome [46, 47]. In the present study the Kawasaki formula did not show J-formed associations with BMI, blood pressure or cholesterol (additional file 1), nor did we observe such associations with atherosclerotic outcomes in our previous study [16]. In a simulation study, when sodium was kept constant in the Kawasaki formula, the associations disappeared [48], suggesting that sodium variation is the predictive factor, not the formula. However, caution is warranted when interpreting sodium values, as the formula may overestimate sodium excretion.

Other limitations include limited data regarding specific use of pharmaceuticals against hypertension, hyperlipidemia and the use of diuretics, the cross-sectional design of the study making it impossible to draw conclusions regarding causality and residual confounding where influence of habitual food intake, food quality, and amount of exercise serve as two particularly important confounders. Further, our study participants represent a population of 50–64-year-old people living in Sweden and therefore our results may not be generalizable to other populations or age groups.

## Conclusion

In this metabolomic study from a contemporary Swedish cohort, lower salt intake was associated with a metabolomic profile characterized by higher levels of free fatty acids and citric acid cycle intermediates, suggestive of increased beta-oxidation as an energy source. In contrast, higher salt intake was linked to a lipidomic pattern enriched in phosphatidylcholines, lysophospholipids, and bile acids previously associated with inflammation and atherosclerosis. Together, these findings provide metabolic insight into how salt intake may influence cardiovascular health beyond its impact on blood pressure. Further longitudinal studies are needed to validate these associations and clarify their relevance for clinical outcomes.

## Supplementary Information

Below is the link to the electronic supplementary material.

Supplementary Material 1

Supplementary Material 2

Supplementary Material 3

Supplementary Material 4

## Data Availability

The personal data in SCAPIS is of a sensitive nature and therefore cannot be made freely available. However, by contacting the corresponding author or study organization ([www.scapis.org](http://www.scapis.org)) sharing of data can be arranged for reproducing study results and procedures.

## Abbreviations

CVD: Cardiovascular Disease
SCAPIS: The Swedish CArdioPulmonary bioImage Study
CC: Chemical Class
Est24hNa: Estimated 24-hour sodium excretion
PCA: Principal Component Analysis
PC1: The first principal component
PCM: Partially Characterized Molecules
FDR: False Discovery Rate
TCA: Tricarboxylic acid
BCAA: Branched Chained Amino Acids

## References

[1] Intersalt. An international study of electrolyte excretion and blood pressure. Results for 24 hour urinary sodium and potassium excretion. Intersalt Coop Res Group Bmj. 1988;297(6644):319–28. 10.1136/bmj.297.6644.319 PMC1834069 3416162

[2] Mente A, O’Donnell MJ, Rangarajan S, McQueen MJ, Poirier P, Wielgosz A, et al. Association of urinary sodium and potassium excretion with blood pressure. N Engl J Med. 2014;371(7):601–11. 25119606 10.1056/NEJMoa1311989

[3] Graudal NA, Hubeck-Graudal T, Jurgens G. Effects of low sodium diet versus high sodium diet on blood pressure, renin, aldosterone, catecholamines, cholesterol, and triglyceride. Cochrane Database Syst Rev. 2020;12(12):Cd004022. 33314019 10.1002/14651858.CD004022.pub5 PMC8094404

[4] Filippini T, Malavolti M, Whelton PK, Vinceti M. Sodium intake and risk of hypertension: A systematic review and Dose-Response Meta-analysis of observational cohort studies. Curr Hypertens Rep. 2022;24(5):133–44. 35246796 10.1007/s11906-022-01182-9

[5] Wang YJ, Yeh TL, Shih MC, Tu YK, Chien KL. Dietary sodium intake and risk of cardiovascular disease: A systematic review and Dose-Response Meta-Analysis. Nutrients. 2020;12(10). 10.3390/nu12102934 PMC7601012 32992705

[6] Ma Y, He FJ, Sun Q, Yuan C, Kieneker LM, Curhan GC, et al. 24-Hour urinary sodium and potassium excretion and cardiovascular risk. N Engl J Med. 2022;386(3):252–63. 34767706 10.1056/NEJMoa2109794 PMC9153854

[7] Zhou L, Stamler J, Chan Q, Van Horn L, Daviglus ML, Dyer AR, et al. Salt intake and prevalence of overweight/obesity in japan, china, the united kingdom, and the united states: the INTERMAP study. Am J Clin Nutr. 2019;110(1):34–40. 31111867 10.1093/ajcn/nqz067 PMC6599742

[8] Wu Q, Burley G, Li LC, Lin S, Shi YC. The role of dietary salt in metabolism and energy balance: insights beyond cardiovascular disease. Diabetes Obes Metab. 2023;25(5):1147–61. 36655379 10.1111/dom.14980 PMC10946535

[9] Chaudhary P, Velkoska E, Wainford RD. An exploratory analysis of comparative plasma metabolomic and lipidomic profiling in salt-sensitive and salt-resistant individuals from the dietary approaches to stop hypertension sodium trial. J Hypertens. 2021;39(10):1972–81. 34001808 10.1097/HJH.0000000000002904 PMC8429079

[10] Jablonski KL, Klawitter J, Chonchol M, Bassett CJ, Racine ML, Seals DR. Effect of dietary sodium restriction on human urinary metabolomic profiles. Clin J Am Soc Nephrol. 2015;10(7):1227–34. 25901092 10.2215/CJN.11531114 PMC4491302

[11] Shi M, He J, Li C, Lu X, He WJ, Cao J, et al. Metabolomics study of blood pressure salt-sensitivity and hypertension. Nutr Metab Cardiovasc Dis. 2022;32(7):1681–92. 35599090 10.1016/j.numecd.2022.04.002 PMC9596959

[12] Chen L, He FJ, Dong Y, Huang Y, Harshfield GA, Zhu H. Sodium reduction, metabolomic profiling, and cardiovascular disease risk in untreated black hypertensives. Hypertension. 2019;74(1):194–200. 31079530 10.1161/HYPERTENSIONAHA.119.12880 PMC9116731

[13] Strauss-Kruger M, van Zyl T, Pieters M, Kruger R, Mokwatsi G, Gafane-Matemane L, et al. Urinary metabolomics, dietary salt intake and blood pressure: the African-PREDICT study. Hypertens Res. 2023;46(1):175–86. 36229536 10.1038/s41440-022-01071-3

[14] Cheng Y, Song H, Pan X, Xue H, Wan Y, Wang T, et al. Urinary metabolites associated with blood pressure on a Low- or High-Sodium diet. Theranostics. 2018;8(6):1468–80. 29556335 10.7150/thno.22018 PMC5858161

[15] Hamaya R, Sun Q, Li J, Yun H, Wang F, Curhan GC, et al. 24-h urinary sodium and potassium excretions, plasma metabolomic profiles, and cardiometabolic biomarkers in the united States adults: a cross-sectional study. Am J Clin Nutr. 2024;120(1):153–61. 38762185 10.1016/j.ajcnut.2024.05.010 PMC11251214

[16] Wuopio J, Ling YT, Orho-Melander M, Engström G, Ärnlöv J. The association between sodium intake and coronary and carotid atherosclerosis in the general Swedish population. Eur Heart J Open. 2023;3(2):oead024. 37006408 10.1093/ehjopen/oead024 PMC10063371

[17] Bergström G, Berglund G, Blomberg A, Brandberg J, Engström G, Engvall J, et al. The Swedish cardiopulmonary bioimage study: objectives and design. J Intern Med. 2015;278(6):645–59. 26096600 10.1111/joim.12384 PMC4744991

[18] Kawasaki T, Itoh K, Uezono K, Sasaki H. A simple method for estimating 24 h urinary sodium and potassium excretion from second morning voiding urine specimen in adults. Clin Exp Pharmacol Physiol. 1993;20(1):7–14. 8432042 10.1111/j.1440-1681.1993.tb01496.x

[19] Ghosh N, Lejonberg C, Czuba T, Dekkers K, Robinson R, Ärnlöv J, et al. Analysis of plasma metabolomes from 11 309 subjects in five population-based cohorts. Sci Rep. 2024;14(1):8933. 38637659 10.1038/s41598-024-59388-7 PMC11026396

[20] Nyman U, Grubb A, Larsson A, Hansson LO, Flodin M, Nordin G, et al. The revised Lund-Malmö GFR estimating equation outperforms MDRD and CKD-EPI across GFR, age and BMI intervals in a large Swedish population. Clin Chem Lab Med. 2014;52(6):815–24. 24334413 10.1515/cclm-2013-0741

[21] Pedersen AB, Mikkelsen EM, Cronin-Fenton D, Kristensen NR, Pham TM, Pedersen L, et al. Missing data and multiple imputation in clinical epidemiological research. Clin Epidemiol. 2017;9:157–66. 28352203 10.2147/CLEP.S129785 PMC5358992

[22] Owen OE, Kalhan SC, Hanson RW. The key role of anaplerosis and cataplerosis for citric acid cycle function. J Biol Chem. 2002;277(34):30409–12. 12087111 10.1074/jbc.R200006200

[23] Longo N, Frigeni M, Pasquali M. Carnitine transport and fatty acid oxidation. Biochim Biophys Acta. 2016;1863(10):2422–35. 26828774 10.1016/j.bbamcr.2016.01.023 PMC4967041

[24] Holeček M. Why are Branched-Chain amino acids increased in starvation and diabetes? Nutrients. 2020;12(10). 10.3390/nu12103087 PMC7600358 33050579

[25] Maurer J, Hoene M, Weigert C. Signals from the circle: Tricarboxylic acid cycle intermediates as myometabokines. Metabolites. 2021;11(8). 10.3390/metabo11080474 PMC8398969 34436415

[26] Owen OE, Smalley KJ, D’Alessio DA, Mozzoli MA, Dawson EK. Protein, fat, and carbohydrate requirements during starvation: anaplerosis and cataplerosis. Am J Clin Nutr. 1998;68(1):12–34. 9665093 10.1093/ajcn/68.1.12

[27] Steinhauser ML, Olenchock BA, O’Keefe J, Lun M, Pierce KA, Lee H et al. The Circulating metabolome of human starvation. JCI Insight. 2018;3(16). 10.1172/jci.insight.121434 PMC6141167 30135314

[28] Takase H, Machii M, Nonaka D, Ohno K, Takayama S, Sugiura T et al. Excessive salt intake is a significant predictor for future development of metabolic syndrome in the general population. Eur Heart J. 2020;41(Supplement_2).

[29] Larsen SC, Ängquist L, Sørensen TI, Heitmann BL. 24 h urinary sodium excretion and subsequent change in weight, waist circumference and body composition. PLoS ONE. 2013;8(7):e69689. 23936079 10.1371/journal.pone.0069689 PMC3723894

[30] Slijkhuis N, Towers M, Mirzaian M, Korteland SA, Heijs B, van Gaalen K, et al. Identifying lipid traces of atherogenic mechanisms in human carotid plaque. Atherosclerosis. 2023;385:117340. 37913561 10.1016/j.atherosclerosis.2023.117340

[31] Barranco-Altirriba M, Rossell J, Alonso N, Weber RJM, Ortega E, Lloyd GR, et al. Lipidomic analysis reveals metabolism alteration associated with subclinical carotid atherosclerosis in type 2 diabetes. Cardiovasc Diabetol. 2025;24(1):152. 40176064 10.1186/s12933-025-02701-z PMC11967040

[32] Simopoulos AP. The importance of the omega-6/omega-3 fatty acid ratio in cardiovascular disease and other chronic diseases. Exp Biol Med (Maywood). 2008;233(6):674–88. 18408140 10.3181/0711-MR-311

[33] Yamagata K. Docosahexaenoic acid regulates vascular endothelial cell function and prevents cardiovascular disease. Lipids Health Dis. 2017;16(1):118. 28619112 10.1186/s12944-017-0514-6 PMC5472966

[34] Law SH, Chan ML, Marathe GK, Parveen F, Chen CH, Ke LY. An updated review of lysophosphatidylcholine metabolism in human diseases. Int J Mol Sci. 2019;20(5). 10.3390/ijms20051149 PMC6429061 30845751

[35] Cheng X, Zhang R, Qi X, Wang H, Gao T, Zheng L, et al. Metabolomics and network Pharmacology exploration of the effects of bile acids on carotid atherosclerosis and potential underlying mechanisms. Front Endocrinol (Lausanne). 2024;15:1430720. 39076513 10.3389/fendo.2024.1430720 PMC11284041

[36] Alnouti Y. Bile acid sulfation: A pathway of bile acid elimination and detoxification. Toxicol Sci. 2009;108(2):225–46. 19131563 10.1093/toxsci/kfn268

[37] Yang HM. Mitochondrial dysfunction in cardiovascular diseases. Int J Mol Sci. 2025;26(5). 10.3390/ijms26051917 PMC11900462 40076543

[38] Lee M, Sorn SR, Lee Y, Kang I. Salt induces adipogenesis/lipogenesis and inflammatory adipocytokines secretion in adipocytes. Int J Mol Sci. 2019;20(1). 10.3390/ijms20010160 PMC6337705 30621146

[39] Lanaspa MA, Kuwabara M, Andres-Hernando A, Li N, Cicerchi C, Jensen T, et al. High salt intake causes leptin resistance and obesity in mice by stimulating endogenous Fructose production and metabolism. Proc Natl Acad Sci U S A. 2018;115(12):3138–43. 29507217 10.1073/pnas.1713837115 PMC5866545

[40] Dobrian AD, Schriver SD, Lynch T, Prewitt RL. Effect of salt on hypertension and oxidative stress in a rat model of diet-induced obesity. Am J Physiol Ren Physiol. 2003;285(4):F619–28. 10.1152/ajprenal.00388.2002 12799306

[41] Fonseca-Alaniz MH, Brito LC, Borges-Silva CN, Takada J, Andreotti S, Lima FB. High dietary sodium intake increases white adipose tissue mass and plasma leptin in rats. Obes (Silver Spring). 2007;15(9):2200–8. 10.1038/oby.2007.261 17890487

[42] Zhang Y, Li F, Liu FQ, Chu C, Wang Y, Wang D et al. Elevation of fasting Ghrelin in healthy human subjects consuming a High-Salt diet: A novel mechanism of obesity? Nutrients. 2016;8(6). 10.3390/nu8060323 PMC4924164 27240398

[43] Drucker DJ. Efficacy and safety of GLP-1 medicines for type 2 diabetes and obesity. Diabetes Care. 2024;47(11):1873–88. 38843460 10.2337/dci24-0003

[44] Zheng WL, Chu C, Lv YB, Wang Y, Hu JW, Ma Q, et al. Effect of salt intake on serum Glucagon-Like Peptide-1 levels in normotensive salt-Sensitive subjects. Kidney Blood Press Res. 2017;42(4):728–37. 29050005 10.1159/000484152

[45] Mente A, O’Donnell MJ, Dagenais G, Wielgosz A, Lear SA, McQueen MJ, et al. Validation and comparison of three formulae to estimate sodium and potassium excretion from a single morning fasting urine compared to 24-h measures in 11 countries. J Hypertens. 2014;32(5):1005–14. discussion 15. 24569420 10.1097/HJH.0000000000000122

[46] Tan M, He FJ, MacGregor GA. Salt and cardiovascular disease in PURE: A large sample size cannot make up for erroneous estimations. J Renin Angiotensin Aldosterone Syst. 2018;19(4):1470320318810015. 30404579 10.1177/1470320318810015 PMC6240978

[47] Campbell NRC. Estimated salt intake and risk of atrial fibrillation in a prospective community-based cohort. J Intern Med. 2021;289(4):591–2. 33277715 10.1111/joim.13221

[48] Wuopio J, Orho-Melander M, Engström G, Ärnlöv J. ‘No research without perfect methods’: a problematic approach in epidemiology. Eur Heart J Open. 2023;3(6). 10.1093/ehjopen/oead093 PMC10634624 37953823

