# Supplementary material for: The metabolic signature of salt intake: a cross-sectional analysis from the SCAPIS-study
Source: Nutr Metab (Lond). 2025 Sep 2;22:104. doi: 10.1186/s12986-025-00997-y (PMC12406461; doi:10.1186/s12986-025-00997-y)
Supplement: Supplementary file 1 — Supplementary Material 1 [file 12986_2025_997_MOESM1_ESM.pdf]

Additional file 2

Supplementary table 2: *Table of lipid metabolites with a significant (p_{bonf} < 0.05) association to est24hNa*

	CHEMICAL NAME	HMDB	SUB PATHWAY	β	p_{bonf}
1	2S,3R-dihydroxybutyrate	HMDB0002453	Fatty Acid, Dihydroxy	-0.108	1.23e-37
2	palmitate (16:0)	HMDB0000220	Long Chain Saturated Fatty Acid	-0.100	3.21e-28
3	oleate/vaccenate (18:1)	HMDB0003231	Long Chain Monounsaturated Fatty Acid	-0.099	2.49e-27
4	acetylcarnitine (C2)	HMDB0000201	Fatty Acid Metabolism (Acyl Carnitine, Short Chain)	-0.100	3.67e-25
5	linoleate (18:2n6)	HMDB0006270	Long Chain Polyunsaturated Fatty Acid (n3 and n6)	-0.093	5.12e-25
6	margarate (17:0)	HMDB0002259	Long Chain Saturated Fatty Acid	-0.091	1.82e-23
7	dihomo-linoleate (20:2n6)	HMDB0005060	Long Chain Polyunsaturated Fatty Acid (n3 and n6)	-0.092	2.67e-23
8	10-heptadecenoate (17:1n7)	HMDB0060038	Long Chain Monounsaturated Fatty Acid	-0.090	3.35e-23
9	eicosenoate (20:1)	HMDB0002231	Long Chain Monounsaturated Fatty Acid	-0.092	6.90e-23
10	10-nonadecenoate (19:1n9)	HMDB0013622	Long Chain Monounsaturated Fatty Acid	-0.089	4.55e-22
11	hexadecanedioate (C16-DC)	HMDB0000672	Fatty Acid, Dicarboxylate	-0.094	5.48e-22
12	(14 or 15)-methylpalmitate (a17:0 or i17:0)	HMDB0061859	Fatty Acid, Branched	-0.085	2.65e-21
13	myristate (14:0)	HMDB0000806	Long Chain Saturated Fatty Acid	-0.087	2.78e-21
14	pentadecanoate (15:0)	HMDB0000826	Long Chain Saturated Fatty Acid	-0.084	1.47e-20
15	palmitoleate (16:1n7)	HMDB0003229	Long Chain Monounsaturated Fatty Acid	-0.084	2.10e-20
16	stearate (18:0)	HMDB0000827	Long Chain Saturated Fatty Acid	-0.083	4.60e-20
17	decadienedioic acid (C10:2-DC)**	unknown	Fatty Acid, Dicarboxylate	-0.089	1.62e-19
18	hexanoylglutamine	unknown	Fatty Acid Metabolism (Acyl Glutamine)	-0.088	1.14e-18
19	3-hydroxydodecanedioate*	HMDB0000413	Fatty Acid, Dicarboxylate	-0.088	1.41e-18
20	dodecenedioate (C12:1-DC)*	HMDB0000933	Fatty Acid, Dicarboxylate	-0.083	7.98e-17
21	16-hydroxypalmitate	HMDB0006294	Fatty Acid, Monohydroxy	-0.082	1.28e-16
22	1-(1-enyl-palmitoyl)-2-oleoyl-GPE (P-16:0/18:1)*	HMDB0011342	Plasmalogen	0.081	1.53e-16
23	hexanoylglycine	HMDB0000701	Fatty Acid Metabolism (Acyl Glycine)	-0.083	2.03e-16
24	3-hydroxyhexanoate	HMDB0061652	Fatty Acid, Monohydroxy	-0.082	2.39e-16

	CHEMICAL NAME	HMDB	SUB PATHWAY	β	p_{bonf}
**25**	docosapentaenoate (n3 DPA; 22:5n3)	HMDB0006528	Long Chain Polyunsaturated Fatty Acid (n3 and n6)	-0.076	3.16e-16
**26**	docosadienoate (22:2n6)	HMDB0061714	Long Chain Polyunsaturated Fatty Acid (n3 and n6)	-0.074	1.08e-15
**27**	1-linolenoyl-GPC (18:3)*	HMDB0010388	Lysophospholipid	0.079	1.21e-15
**28**	adrenate (22:4n6)	HMDB0002226	Long Chain Polyunsaturated Fatty Acid (n3 and n6)	-0.075	3.80e-15
**29**	1,2-dilinoleoyl-GPC (18:2/18:2)	HMDB0008138	Phosphatidylcholine (PC)	0.070	3.98e-14
**30**	octadecanedioate (C18-DC)	HMDB0000782	Fatty Acid, Dicarboxylate	-0.074	1.86e-13
**31**	heptenedioate (C7:1-DC)*	unknown	Fatty Acid, Dicarboxylate	-0.075	2.14e-13
**32**	3-hydroxyoctanoate	HMDB0001954	Fatty Acid, Monohydroxy	-0.073	3.39e-13
**33**	3-hydroxydecanoate	HMDB0002203	Fatty Acid, Monohydroxy	-0.073	4.30e-13
**34**	nonadecanoate (19:0)	HMDB0000772	Long Chain Saturated Fatty Acid	-0.064	4.33e-13
**35**	3-hydroxysebacate	HMDB0000350	Fatty Acid, Monohydroxy	-0.078	5.99e-13
**36**	(R)-3-hydroxybutyrylcarnitine	HMDB0013127	Fatty Acid Metabolism (Acyl Carnitine, Hydroxy)	-0.074	6.04e-13
**37**	3-hydroxylaurate	HMDB0000387	Fatty Acid, Monohydroxy	-0.073	9.40e-13
**38**	1-linoleoyl-2-linolenoyl-GPC (18:2/18:3)*	HMDB0008141	Phosphatidylcholine (PC)	0.068	1.58e-12
**39**	dihomo-linolenate (20:3n3 or n6)	HMDB0002925	Long Chain Polyunsaturated Fatty Acid (n3 and n6)	-0.067	2.62e-12
**40**	tetradecanedioate (C14-DC)	HMDB0000872	Fatty Acid, Dicarboxylate	-0.072	2.69e-12
**41**	trans-2-hexenoylglycine	unknown	Fatty Acid Metabolism (Acyl Glycine)	-0.070	2.90e-12
**42**	1-(1-enyl-stearoyl)-2-oleoyl-GPE (P-18:0/18:1)	HMDB0011375	Plasmalogen	0.069	3.98e-12
**43**	N-oleoyltaurine	unknown	Endocannabinoid	-0.074	4.39e-12
**44**	3-hydroxymyristate	unknown	Fatty Acid, Monohydroxy	-0.070	7.75e-12
**45**	arachidate (20:0)	HMDB0002212	Long Chain Saturated Fatty Acid	-0.066	8.52e-12
**46**	1-(1-enyl-stearoyl)-2-linoleoyl-GPE (P-18:0/18:2)*	HMDB0011376	Plasmalogen	0.067	8.87e-12
**47**	docosatrienoate (22:3n3)	HMDB0002823	Long Chain Polyunsaturated Fatty Acid (n3 and n6)	-0.068	1.08e-11
**48**	1-stearoyl-2-linoleoyl-GPC (18:0/18:2)*	HMDB0008039	Phosphatidylcholine (PC)	0.063	1.50e-11
**49**	5-dodecenoylcarnitine (C12:1)	HMDB13326	Fatty Acid Metabolism (Acyl Carnitine, Monounsaturated)	-0.069	1.80e-11
**50**	myristoleoylcarnitine (C14:1)*	HMDB0240588	Fatty Acid Metabolism (Acyl Carnitine, Monounsaturated)	-0.069	2.63e-11

	CHEMICAL NAME	HMDB	SUB PATHWAY	β	p_{bonf}
**51**	1-(1-enyl-palmitoyl)-2-linoleoyl-GPE (P-16:0/18:2)*	HMDB0011343	Plasmalogen	0.068	2.80e-11
**52**	1-(1-enyl-palmitoyl)-2-arachidonoyl-GPE (P-16:0/20:4)*	HMDB0011352	Plasmalogen	0.068	3.64e-11
**53**	dodecadienoate (12:2)*	unknown	Fatty Acid, Dicarboxylate	-0.066	6.03e-11
**54**	hexadecadienoate (16:2n6)	HMDB0000477	Long Chain Polyunsaturated Fatty Acid (n3 and n6)	-0.064	9.09e-11
**55**	taurocholenate sulfate*	unknown	Secondary Bile Acid Metabolism	-0.067	9.27e-11
**56**	(16 or 17)-methylstearate (a19:0 or i19:0)	HMDB0037397	Fatty Acid, Branched	-0.060	1.73e-10
**57**	hyocholate	HMDB0000760	Secondary Bile Acid Metabolism	0.066	3.24e-10
**58**	1-stearoyl-GPC (18:0)	HMDB0010384	Lysophospholipid	0.051	3.83e-10
**59**	octadecenedioate (C18:1-DC)	unknown	Fatty Acid, Dicarboxylate	-0.065	4.00e-10
**60**	cortisone	HMDB0002802	Corticosteroids	-0.060	4.88e-10
**61**	(2 or 3)-decenoate (10:1n7 or n8)	unknown	Medium Chain Fatty Acid	-0.064	6.28e-10
**62**	myristoleate (14:1n5)	HMDB0002000	Long Chain Monounsaturated Fatty Acid	-0.058	4.84e-09
**63**	sphingomyelin (d18:1/18:1, d18:2/18:0)	HMDB0012101	Sphingomyelins	-0.053	9.92e-09
**64**	1-(1-enyl-stearoyl)-2-arachidonoyl-GPE (P-18:0/20:4)*	HMDB0005779	Plasmalogen	0.060	1.23e-08
**65**	stearoyl sphingomyelin (d18:1/18:0)	HMDB0001348	Sphingomyelins	-0.057	1.37e-08
**66**	linolenate [alpha or gamma; (18:3n3 or 6)]	HMDB0003073	Long Chain Polyunsaturated Fatty Acid (n3 and n6)	-0.059	1.44e-08
**67**	palmitoleoylcarnitine (C16:1)*	unknown	Fatty Acid Metabolism (Acyl Carnitine, Monounsaturated)	-0.057	3.11e-08
**68**	3-hydroxydecanoylcarnitine	HMDB0061636	Fatty Acid Metabolism (Acyl Carnitine, Hydroxy)	-0.058	3.11e-08
**69**	andro steroid monosulfate C19H28O6S (1)*	HMDB0002759	Androgenic Steroids	-0.059	3.27e-08
**70**	5-dodecenoate (12:1n7)	HMDB0000529	Medium Chain Fatty Acid	-0.058	3.53e-08
**71**	oleoyl ethanolamide	HMDB0002088	Endocannabinoid	-0.054	5.14e-08
**72**	octadecenedioylcarnitine (C18:1-DC)*	unknown	Fatty Acid Metabolism (Acyl Carnitine, Dicarboxylate)	-0.058	8.52e-08
**73**	5-hydroxyhexanoate	HMDB0000409	Fatty Acid, Monohydroxy	-0.061	1.06e-07

	CHEMICAL NAME	HMDB	SUB PATHWAY	β	p_{bonf}
**74**	myristoylcarnitine (C14)	HMDB000506	Fatty Acid Metabolism (Acyl Carnitine, Long Chain Saturated)	-0.057	1.39e-07
**75**	N-palmitoylglycine	HMDB0013034	Fatty Acid Metabolism (Acyl Glycine)	-0.052	1.74e-07
**76**	3-hydroxyadipate	HMDB0000345	Fatty Acid, Dicarboxylate	-0.057	2.11e-07
**77**	1-palmitoyl-2-docosahexaenoyl-GPC (16:0/22:6)	HMDB0007991	Phosphatidylcholine (PC)	-0.052	3.09e-07
**78**	docosahexaenoate (DHA; 22:6n3)	HMDB0002183	Long Chain Polyunsaturated Fatty Acid (n3 and n6)	-0.053	3.40e-07
**79**	sebacate (C10-DC)	HMDB0000792	Fatty Acid, Dicarboxylate	-0.057	3.67e-07
**80**	(S)-3-hydroxybutyrylcarnitine	HMDB0013127	Fatty Acid Metabolism (Acyl Carnitine, Hydroxy)	-0.056	4.49e-07
**81**	oleoylcarnitine (C18:1)	HMDB0005065	Fatty Acid Metabolism (Acyl Carnitine, Monounsaturated)	-0.054	4.74e-07
**82**	arachidonate (20:4n6)	HMDB0001043	Long Chain Polyunsaturated Fatty Acid (n3 and n6)	-0.052	5.59e-07
**83**	tetradecadienedioate (C14:2-DC)*	unknown	Fatty Acid, Dicarboxylate	-0.055	9.75e-07
**84**	1-myristoyl-2-arachidonoyl-GPC (14:0/20:4)*	HMDB0007883	Phosphatidylcholine (PC)	0.051	9.87e-07
**85**	sphingomyelin (d18:0/18:0, d19:0/17:0)*	HMDB0012087	Dihydrosphingomyelins	-0.047	1.90e-06
**86**	3-hydroxyoctanoylcarnitine (2)	unknown	Fatty Acid Metabolism (Acyl Carnitine, Hydroxy)	-0.052	2.23e-06
**87**	cis-4-decenoylcarnitine (C10:1)	HMDB0013205	Fatty Acid Metabolism (Acyl Carnitine, Monounsaturated)	-0.053	2.98e-06
**88**	3-hydroxyhexanoylcarnitine (1)	unknown	Fatty Acid Metabolism (Acyl Carnitine, Hydroxy)	-0.053	3.09e-06
**89**	linoleoyl ethanolamide	HMDB0012252	Endocannabinoid	-0.052	3.79e-06
**90**	2-hydroxypalmitate	HMDB0031057	Fatty Acid, Monohydroxy	-0.048	4.17e-06
**91**	3-hydroxyoctanoylcarnitine (1)	unknown	Fatty Acid Metabolism (Acyl Carnitine, Hydroxy)	-0.051	4.46e-06
**92**	myo-inositol	HMDB0000211	Inositol Metabolism	-0.053	5.41e-06
**93**	adipoylcarnitine (C6-DC)	HMDB0061677	Fatty Acid Metabolism (Acyl Carnitine, Dicarboxylate)	-0.052	7.33e-06
**94**	laurylcarnitine (C12)	HMDB000225	Fatty Acid Metabolism (Acyl Carnitine, Medium Chain)	-0.050	1.44e-05
**95**	1-palmitoyl-2-stearoyl-GPC (16:0/18:0)	HMDB0007970	Phosphatidylcholine (PC)	0.041	2.60e-05
**96**	tridecenedioate (C13:1-DC)*	unknown	Fatty Acid, Dicarboxylate	-0.049	2.81e-05

	CHEMICAL NAME	HMDB	SUB PATHWAY	β	p_{bonf}
**97**	1-(1-enyl-palmitoyl)-2-linoleoyl-GPC (P-16:0/18:2)*	HMDB0011211	Plasmalogen	0.044	3.64e-05
**98**	linoleoylcholine*	HMDB0013213	Fatty Acid Metabolism (Acyl Choline)	0.052	4.12e-05
**99**	cholate	HMDB0000619	Primary Bile Acid Metabolism	0.049	4.80e-05
**100**	pimeloylcarnitine/3-methyladipoylcarnitine (C7-DC)	unknown	Fatty Acid Metabolism (Acyl Carnitine, Dicarboxylate)	-0.046	9.63e-05
**101**	dodecanedioate (C12-DC)	HMDB0000623	Fatty Acid, Dicarboxylate	-0.047	1.15e-04
**102**	tetradecadienoate (14:2)*	HMDB0000560	Long Chain Polyunsaturated Fatty Acid (n3 and n6)	-0.047	1.21e-04
**103**	androstenediol (3beta,17beta) disulfate (2)	HMDB0240313	Androgenic Steroids	-0.041	1.65e-04
**104**	eicosanedioate (C20-DC)	unknown	Fatty Acid, Dicarboxylate	0.046	2.15e-04
**105**	pregnenetriol disulfate*	unknown	Pregnenolone Steroids	-0.042	2.28e-04
**106**	3-hydroxybutyrate (BHBA)	HMDB0000442	Ketone Bodies	-0.046	2.67e-04
**107**	13-HODE + 9-HODE	HMDB0004670	Fatty Acid, Monohydroxy	-0.045	2.77e-04
**108**	1-linoleoyl-GPC (18:2)	HMDB0010386	Lysophospholipid	0.041	3.03e-04
**109**	1-myristoyl-2-palmitoyl-GPC (14:0/16:0)	HMDB0007869	Phosphatidylcholine (PC)	0.045	3.11e-04
**110**	2R,3R-dihydroxybutyrate	HMDB0000498	Fatty Acid, Dihydroxy	0.045	3.43e-04
**111**	hexanoylcarnitine (C6)	HMDB0000756	Fatty Acid Metabolism (Acyl Carnitine, Medium Chain)	-0.046	3.53e-04
**112**	pregnenediol disulfate (C21H34O8S2)*	unknown	Pregnenolone Steroids	-0.041	4.85e-04
**113**	1-(1-enyl-palmitoyl)-GPC (P-16:0)*	HMDB0010407	Lysoplasmalogen	0.040	4.95e-04
**114**	3-hydroxy-3-methylglutarate	HMDB0000355	Mevalonate Metabolism	-0.043	5.85e-04
**115**	deoxycholic acid glucuronide	unknown	Secondary Bile Acid Metabolism	0.044	6.26e-04
**116**	1-oleoyl-GPC (18:1)	HMDB0002815	Lysophospholipid	0.040	6.77e-04
**117**	1-palmitoleoyl-2-linolenoyl-GPC (16:1/18:3)*	HMDB0008008	Phosphatidylcholine (PC)	0.041	7.66e-04
**118**	cholic acid glucuronide	HMDB0002577	Primary Bile Acid Metabolism	0.046	9.99e-04
**119**	epiandrosterone sulfate	HMDB0062657	Androgenic Steroids	-0.040	1.03e-03
**120**	1-(1-enyl-palmitoyl)-2-arachidonoyl-GPC (P-16:0/20:4)*	HMDB0011220	Plasmalogen	0.042	1.07e-03
**121**	10-undecenoate (11:1n1)	HMDB0033724	Medium Chain Fatty Acid	-0.043	1.37e-03

	CHEMICAL NAME	HMDB	SUB PATHWAY	β	p_{bonf}
**122**	androstenediol (3beta,17beta) disulfate (1)	HMDB0240313	Androgenic Steroids	-0.041	1.40e-03
**123**	androstenediol (3beta,17beta) monosulfate (1)	HMDB0240429	Androgenic Steroids	-0.039	1.43e-03
**124**	5alpha-androstan-3beta,17beta-diol monosulfate (2)	unknown	Androgenic Steroids	-0.039	1.67e-03
**125**	linoleoylcarnitine (C18:2)*	HMDB0006469	Fatty Acid Metabolism (Acyl Carnitine, Polyunsaturated)	-0.038	1.67e-03
**126**	palmitoylcholine	HMDB0240592	Fatty Acid Metabolism (Acyl Choline)	0.042	1.92e-03
**127**	hexadecenedioate (C16:1-DC)*	unknown	Fatty Acid, Dicarboxylate	-0.042	1.94e-03
**128**	taurolithocholate 3-sulfate	HMDB0002580	Secondary Bile Acid Metabolism	-0.042	2.19e-03
**129**	sphingomyelin (d18:1/17:0, d17:1/18:0, d19:1/16:0)	unknown	Sphingomyelins	-0.037	2.31e-03
**130**	docosatrienoate (22:3n6)*	unknown	Long Chain Polyunsaturated Fatty Acid (n3 and n6)	-0.044	2.42e-03
**131**	N-oleoylserine	unknown	Endocannabinoid	-0.041	2.65e-03
**132**	palmitoylcarnitine (C16)	HMDB0000222	Fatty Acid Metabolism (Acyl Carnitine, Long Chain Saturated)	-0.037	3.95e-03
**133**	cortisol	HMDB0000063	Corticosteroids	-0.040	4.19e-03
**134**	16a-hydroxy DHEA 3-sulfate	HMDB0062544	Androgenic Steroids	-0.040	4.30e-03
**135**	N-stearoyl-sphingosine (d18:1/18:0)*	HMDB0004950	Ceramides	-0.035	4.62e-03
**136**	1-stearoyl-2-oleoyl-GPC (18:0/18:1)	HMDB0008038	Phosphatidylcholine (PC)	0.037	5.35e-03
**137**	7-alpha-hydroxy-3-oxo-4-cholestenoate (7-Hoca)	HMDB0012458	Sterol	-0.039	6.15e-03
**138**	2-butenoylglycine	unknown	Fatty Acid Metabolism (Acyl Glycine)	-0.038	6.60e-03
**139**	chenodeoxycholate	HMDB0000518	Primary Bile Acid Metabolism	0.039	1.15e-02
**140**	malonylcarnitine	HMDB0002095	Fatty Acid Synthesis	-0.041	1.18e-02
**141**	21-hydroxypregnenolone disulfate	unknown	Pregnenolone Steroids	-0.036	1.20e-02
**142**	3beta-hydroxy-5-cholestenoate	unknown	Sterol	-0.033	1.58e-02
**143**	lactosyl-N-palmitoyl-sphingosine (d18:1/16:0)	unknown	Lactosylceramides (LCER)	-0.036	1.70e-02

	CHEMICAL NAME	HMDB	SUB PATHWAY	β	p_{bonf}
**144**	maleate	HMDB0000176	Fatty Acid, Dicarboxylate	0.038	1.79e-02
**145**	dehydroepiandrosterone sulfate (DHEA-S)	HMDB0001032	Androgenic Steroids	-0.034	2.09e-02
**146**	5alpha-androstan-3alpha,17beta-diol disulfate	HMDB0094682	Androgenic Steroids	-0.032	2.66e-02
**147**	5alpha-androstan-3beta,17beta-diol disulfate	HMDB00493	Androgenic Steroids	-0.033	2.69e-02
**148**	1-(1-enyl-oleoyl)-GPE (P-18:1)*	unknown	Lysoplasmalogen	0.035	3.37e-02
**149**	deoxycholate	HMDB0000626	Secondary Bile Acid Metabolism	0.036	4.86e-02
