# Supplementary material for: The metabolic signature of salt intake: a cross-sectional analysis from the SCAPIS-study
Source: Nutr Metab (Lond). 2025 Sep 2;22:104. doi: 10.1186/s12986-025-00997-y (PMC12406461; doi:10.1186/s12986-025-00997-y)
Supplement: Supplementary file 2 — Supplementary Material 2 [file 12986_2025_997_MOESM2_ESM.pdf]

Supplementary data 3.

Table of all metabolites included in the study with estimates (β) and Bonferroni-corrected p-values.

	CHEMICAL NAME	HMDB	CHEMICAL CLASS	SUB PATHWAY	β	P_{bonf}
**1**	2S,3R-dihydroxybutyrate	HMDB0002453	Lipid	Fatty Acid, Dihydroxy	-0.133	2.28e-37
**2**	homovanillate (HVA)	HMDB0000118	Amino Acid	Tyrosine Metabolism	-0.147	4.64e-37
**3**	N,N,N-trimethyl-alanylproline betaine (TMAP)	HMDB0240365	Amino Acid	Urea cycle; Arginine and Proline Metabolism	-0.124	4.35e-30
**4**	2-hydroxyphenylacetate	HMDB0000669	Amino Acid	Phenylalanine Metabolism	-0.127	4.82e-28
**5**	palmitate (16:0)	HMDB0000220	Lipid	Long Chain Saturated Fatty Acid	-0.123	6.71e-28
**6**	N,N-dimethyl-pro-pro	unknown	Peptide	Modified Peptides	-0.119	2.01e-27
**7**	oleate/vaccenate (18:1)	HMDB0003231	Lipid	Long Chain Monounsaturated Fatty Acid	-0.122	5.51e-27
**8**	acetylcarnitine (C2)	HMDB0000201	Lipid	Fatty Acid Metabolism (Acyl Carnitine, Short Chain)	-0.123	7.56e-25
**9**	linoleate (18:2n6)	HMDB0006270	Lipid	Long Chain Polyunsaturated Fatty Acid (n3 and n6)	-0.115	1.03e-24
**10**	alpha-hydroxyisocaproate	HMDB0000665	Amino Acid	Leucine, Isoleucine and Valine Metabolism	-0.107	1.23e-23
**11**	creatinine	HMDB0000562	Amino Acid	Creatine Metabolism	-0.100	1.78e-23
**12**	margarate (17:0)	HMDB0002259	Lipid	Long Chain Saturated Fatty Acid	-0.111	3.67e-23
**13**	dihomo-linoleate (20:2n6)	HMDB0005060	Lipid	Long Chain Polyunsaturated Fatty Acid (n3 and n6)	-0.113	5.76e-23
**14**	10-heptadecenoate (17:1n7)	HMDB0060038	Lipid	Long Chain Monounsaturated Fatty Acid	-0.111	7.01e-23
**15**	eicosenoate (20:1)	HMDB0002231	Lipid	Long Chain Monounsaturated Fatty Acid	-0.113	1.60e-22
**16**	3-hydroxy-2-ethylpropionate	HMDB0000396	Amino Acid	Leucine, Isoleucine and Valine Metabolism	-0.116	3.42e-22
**17**	10-nonadecenoate (19:1n9)	HMDB0013622	Lipid	Long Chain Monounsaturated Fatty Acid	-0.110	8.85e-22
**18**	hexadecanedioate (C16-DC)	HMDB0000672	Lipid	Fatty Acid, Dicarboxylate	-0.116	1.21e-21

	CHEMICAL NAME	HMDB	CHEMICAL CLASS	SUB PATHWAY	β	P_{bonf}
**19**	(14 or 15)-methylpalmitate (a17:0 or i17:0)	HMDB0061859	Lipid	Fatty Acid, Branched	-0.105	4.63e-21
**20**	myristate (14:0)	HMDB0000806	Lipid	Long Chain Saturated Fatty Acid	-0.107	5.04e-21
**21**	trans-4-hydroxyproline	HMDB0000725	Amino Acid	Urea cycle; Arginine and Proline Metabolism	0.113	6.74e-21
**22**	pentadecanoate (15:0)	HMDB0000826	Lipid	Long Chain Saturated Fatty Acid	-0.104	2.61e-20
**23**	palmitoleate (16:1n7)	HMDB0003229	Lipid	Long Chain Monounsaturated Fatty Acid	-0.104	4.54e-20
**24**	stearate (18:0)	HMDB0000827	Lipid	Long Chain Saturated Fatty Acid	-0.102	8.93e-20
**25**	decadienedioic acid (C10:2-DC)**	unknown	Lipid	Fatty Acid, Dicarboxylate	-0.109	4.00e-19
**26**	vanillylmandelate (VMA)	HMDB0000291	Amino Acid	Tyrosine Metabolism	-0.103	1.76e-18
**27**	hexanoylglutamine	unknown	Lipid	Fatty Acid Metabolism (Acyl Glutamine)	-0.108	2.44e-18
**28**	3-hydroxydodecanedioate*	HMDB0000413	Lipid	Fatty Acid, Dicarboxylate	-0.108	2.99e-18
**29**	urate	HMDB0000289	Nucleotide	Purine Metabolism, (Hypo)Xanthine/Inosine containing	-0.086	1.13e-17
**30**	dodecenedioate (C12:1-DC)*	HMDB0000933	Lipid	Fatty Acid, Dicarboxylate	-0.103	1.42e-16
**31**	hexanoylglycine	HMDB0000701	Lipid	Fatty Acid Metabolism (Acyl Glycine)	-0.103	2.72e-16
**32**	1-(1-enyl-palmitoyl)-2-oleoyl-GPE (P-16:0/18:1)*	HMDB0011342	Lipid	Plasmalogen	0.100	2.91e-16
**33**	16-hydroxypalmitate	HMDB0006294	Lipid	Fatty Acid, Monohydroxy	-0.101	2.98e-16
**34**	3-hydroxyhexanoate	HMDB0061652	Lipid	Fatty Acid, Monohydroxy	-0.101	5.38e-16
**35**	docosapentaenoate (n3 DPA; 22:5n3)	HMDB0006528	Lipid	Long Chain Polyunsaturated Fatty Acid (n3 and n6)	-0.093	6.52e-16
**36**	1-linolenoyl-GPC (18:3)*	HMDB0010388	Lipid	Lysophospholipid	0.098	2.42e-15

	CHEMICAL NAME	HMDB	CHEMICAL CLASS	SUB PATHWAY	β	P_{bonf}
**37**	docosadienoate (22:2n6)	HMDB0061714	Lipid	Long Chain Polyunsaturated Fatty Acid (n3 and n6)	-0.091	2.47e-15
**38**	adrenate (22:4n6)	HMDB0002226	Lipid	Long Chain Polyunsaturated Fatty Acid (n3 and n6)	-0.092	8.96e-15
**39**	2-hydroxybutyrate/2-hydroxyisobutyrate	HMDB0000729	Amino Acid	Glutathione Metabolism	-0.092	3.23e-14
**40**	3-hydroxyisobutyrate	HMDB0000023	Amino Acid	Leucine, Isoleucine and Valine Metabolism	-0.094	7.77e-14
**41**	1,2-dilinoleoyl-GPC (18:2/18:2)	HMDB0008138	Lipid	Phosphatidylcholine (PC)	0.086	9.20e-14
**42**	4-methyl-2-oxopentanoate	HMDB0000695	Amino Acid	Leucine, Isoleucine and Valine Metabolism	-0.078	1.06e-13
**43**	octadecanedioate (C18-DC)	HMDB0000782	Lipid	Fatty Acid, Dicarboxylate	-0.091	3.72e-13
**44**	N4-acetylcytidine	HMDB0005923	Nucleotide	Pyrimidine Metabolism, Cytidine containing	-0.092	4.03e-13
**45**	heptenedioate (C7:1-DC)*	unknown	Lipid	Fatty Acid, Dicarboxylate	-0.092	4.12e-13
**46**	3-amino-2-piperidone	HMDB0000323	Amino Acid	Urea cycle; Arginine and Proline Metabolism	0.091	4.70e-13
**47**	nonadecanoate (19:0)	HMDB0000772	Lipid	Long Chain Saturated Fatty Acid	-0.079	7.83e-13
**48**	3-hydroxyoctanoate	HMDB0001954	Lipid	Fatty Acid, Monohydroxy	-0.089	8.07e-13
**49**	3-hydroxydecanoate	HMDB0002203	Lipid	Fatty Acid, Monohydroxy	-0.090	8.65e-13
**50**	3-hydroxysebacate	HMDB0000350	Lipid	Fatty Acid, Monohydroxy	-0.096	1.37e-12
**51**	alpha-ketobutyrate	HMDB0000005	Amino Acid	Methionine, Cysteine, SAM and Taurine Metabolism	-0.088	1.46e-12
**52**	(R)-3-hydroxybutyrylcarnitine	HMDB0013127	Lipid	Fatty Acid Metabolism (Acyl Carnitine, Hydroxy)	-0.090	1.53e-12
**53**	3-hydroxylaurate	HMDB0000387	Lipid	Fatty Acid, Monohydroxy	-0.090	1.78e-12
**54**	1-linoleoyl-2-linolenoyl-GPC (18:2/18:3)*	HMDB0008141	Lipid	Phosphatidylcholine (PC)	0.084	3.52e-12

	CHEMICAL NAME	HMDB	CHEMICAL CLASS	SUB PATHWAY	β	P_{bonf}
**55**	trans-2-hexenoylglycine	unknown	Lipid	Fatty Acid Metabolism (Acyl Glycine)	-0.086	4.78e-12
**56**	dihomo-linolenate (20:3n3 or n6)	HMDB0002925	Lipid	Long Chain Polyunsaturated Fatty Acid (n3 and n6)	-0.082	5.29e-12
**57**	tetradecanedioate (C14-DC)	HMDB0000872	Lipid	Fatty Acid, Dicarboxylate	-0.088	5.31e-12
**58**	1-(1-enyl-stearoyl)-2-oleoyl-GPE (P-18:0/18:1)	HMDB0011375	Lipid	Plasmalogen	0.085	6.98e-12
**59**	N-oleoyltaurine	unknown	Lipid	Endocannabinoid	-0.091	9.18e-12
**60**	xanthosine	HMDB0000299	Nucleotide	Purine Metabolism, (Hypo)Xanthine/Inosine containing	-0.090	1.29e-11
**61**	3-hydroxymyristate	unknown	Lipid	Fatty Acid, Monohydroxy	-0.086	1.46e-11
**62**	arachidate (20:0)	HMDB0002212	Lipid	Long Chain Saturated Fatty Acid	-0.082	1.47e-11
**63**	1-(1-enyl-stearoyl)-2-linoleoyl-GPE (P-18:0/18:2)*	HMDB0011376	Lipid	Plasmalogen	0.082	2.24e-11
**64**	docosatrienoate (22:3n3)	HMDB0002823	Lipid	Long Chain Polyunsaturated Fatty Acid (n3 and n6)	-0.084	2.30e-11
**65**	citraconate/glutaconate	HMDB0000634	Energy	TCA Cycle	0.086	2.63e-11
**66**	1-stearoyl-2-linoleoyl-GPC (18:0/18:2)*	HMDB0008039	Lipid	Phosphatidylcholine (PC)	0.077	3.54e-11
**67**	5-dodecenoylcarnitine (C12:1)	HMDB13326	Lipid	Fatty Acid Metabolism (Acyl Carnitine, Monounsaturated)	-0.084	3.68e-11
**68**	ornithine	HMDB0000214	Amino Acid	Urea cycle; Arginine and Proline Metabolism	0.084	4.03e-11
**69**	myristoleoylcarnitine (C14:1)*	HMDB0240588	Lipid	Fatty Acid Metabolism (Acyl Carnitine, Monounsaturated)	-0.085	5.46e-11
**70**	3-(4-hydroxyphenyl)lactate	HMDB0000755	Amino Acid	Tyrosine Metabolism	-0.077	6.62e-11
**71**	1-(1-enyl-palmitoyl)-2-arachidonoyl-GPE (P-16:0/20:4)*	HMDB0011352	Lipid	Plasmalogen	0.083	6.64e-11

	CHEMICAL NAME	HMDB	CHEMICAL CLASS	SUB PATHWAY	β	P_{bonf}
**72**	1-(1-enyl-palmitoyl)-2-linoleoyl-GPE (P-16:0/18:2)*	HMDB0011343	Lipid	Plasmalogen	0.084	6.98e-11
**73**	dodecadienoate (12:2)*	unknown	Lipid	Fatty Acid, Dicarboxylate	-0.081	1.09e-10
**74**	branched-chain, straight-chain, or cyclopropyl 12:1 fatty acid*	unknown	Partially Characterized Molecules	Partially Characterized Molecules	-0.082	1.56e-10
**75**	hexadecadienoate (16:2n6)	HMDB0000477	Lipid	Long Chain Polyunsaturated Fatty Acid (n3 and n6)	-0.079	1.71e-10
**76**	N-acetylglycine	HMDB0000532	Amino Acid	Glycine, Serine and Threonine Metabolism	-0.079	1.93e-10
**77**	aconitate [cis or trans]	HMDB0000958	Energy	TCA Cycle	-0.078	2.29e-10
**78**	taurocholenate sulfate*	unknown	Lipid	Secondary Bile Acid Metabolism	-0.083	2.43e-10
**79**	(16 or 17)-methylstearate (a19:0 or i19:0)	HMDB0037397	Lipid	Fatty Acid, Branched	-0.074	3.28e-10
**80**	alpha-hydroxyisovalerate	HMDB0000407	Amino Acid	Leucine, Isoleucine and Valine Metabolism	-0.080	4.15e-10
**81**	hyocholate	HMDB0000760	Lipid	Secondary Bile Acid Metabolism	0.081	6.61e-10
**82**	octadecenedioate (C18:1-DC)	unknown	Lipid	Fatty Acid, Dicarboxylate	-0.080	7.78e-10
**83**	1-stearoyl-GPC (18:0)	HMDB0010384	Lipid	Lysophospholipid	0.063	8.44e-10
**84**	cortisone	HMDB0002802	Lipid	Corticosteroids	-0.074	9.14e-10
**85**	(2 or 3)-decenoate (10:1n7 or n8)	unknown	Lipid	Medium Chain Fatty Acid	-0.079	1.13e-09
**86**	acisoga	HMDB0061384	Amino Acid	Polyamine Metabolism	-0.079	2.32e-09
**87**	myristoleate (14:1n5)	HMDB0002000	Lipid	Long Chain Monounsaturated Fatty Acid	-0.071	9.05e-09
**88**	sphingomyelin (d18:1/18:1, d18:2/18:0)	HMDB0012101	Lipid	Sphingomyelins	-0.065	1.67e-08
**89**	3-methyl-2-oxovalerate	HMDB0000491	Amino Acid	Leucine, Isoleucine and Valine Metabolism	-0.064	1.69e-08
**90**	succinoyltaurine	unknown	Amino Acid	Methionine, Cysteine, SAM and Taurine Metabolism	-0.076	1.75e-08

	CHEMICAL NAME	HMDB	CHEMICAL CLASS	SUB PATHWAY	β	P_{bonf}
**91**	1-(1-enyl-stearoyl)-2-arachidonoyl-GPE (P-18:0/20:4)*	HMDB0005779	Lipid	Plasmalogen	0.074	1.80e-08
**92**	threonine	HMDB0000167	Amino Acid	Glycine, Serine and Threonine Metabolism	0.074	2.60e-08
**93**	stearoyl sphingomyelin (d18:1/18:0)	HMDB0001348	Lipid	Sphingomyelins	-0.070	2.64e-08
**94**	malate	HMDB0031518	Energy	TCA Cycle	-0.073	2.82e-08
**95**	linolenate [alpha or gamma; (18:3n3 or 6)]	HMDB0003073	Lipid	Long Chain Polyunsaturated Fatty Acid (n3 and n6)	-0.072	3.07e-08
**96**	3-hydroxydecanoylcarnitine	HMDB0061636	Lipid	Fatty Acid Metabolism (Acyl Carnitine, Hydroxy)	-0.071	5.26e-08
**97**	erythronate*	HMDB0000613	Carbohydrate	Aminosugar Metabolism	-0.070	5.76e-08
**98**	palmitoleoylcarnitine (C16:1)*	unknown	Lipid	Fatty Acid Metabolism (Acyl Carnitine, Monounsaturated)	-0.069	6.39e-08
**99**	andro steroid monosulfate C19H28O6S (1)*	HMDB0002759	Lipid	Androgenic Steroids	-0.072	6.54e-08
**100**	5-dodecenoate (12:1n7)	HMDB0000529	Lipid	Medium Chain Fatty Acid	-0.071	6.80e-08
**101**	2-hydroxy-3-methylvalerate	HMDB0000317	Amino Acid	Leucine, Isoleucine and Valine Metabolism	-0.070	8.60e-08
**102**	oleoyl ethanolamide	HMDB0002088	Lipid	Endocannabinoid	-0.067	9.64e-08
**103**	phenyllactate (PLA)	HMDB0000779	Amino Acid	Phenylalanine Metabolism	-0.069	1.37e-07
**104**	octadecenedioylcarnitine (C18:1-DC)*	unknown	Lipid	Fatty Acid Metabolism (Acyl Carnitine, Dicarboxylate)	-0.071	1.72e-07
**105**	5-hydroxyhexanoate	HMDB0000409	Lipid	Fatty Acid, Monohydroxy	-0.075	2.07e-07
**106**	N-palmitoylglycine	HMDB0013034	Lipid	Fatty Acid Metabolism (Acyl Glycine)	-0.064	2.75e-07

	CHEMICAL NAME	HMDB	CHEMICAL CLASS	SUB PATHWAY	β	P_{bonf}
**107**	myristoylcarnitine (C14)	HMDB000506	Lipid	Fatty Acid Metabolism (Acyl Carnitine, Long Chain Saturated)	-0.070	2.78e-07
**108**	N-acetylthreonine	HMDB0062557	Amino Acid	Glycine, Serine and Threonine Metabolism	-0.067	4.57e-07
**109**	5,6-dihydrothymine	HMDB0000079	Nucleotide	Pyrimidine Metabolism, Thymine containing	-0.070	4.78e-07
**110**	3-hydroxyadipate	HMDB0000345	Lipid	Fatty Acid, Dicarboxylate	-0.070	5.16e-07
**111**	docosahexaenoate (DHA; 22:6n3)	HMDB0002183	Lipid	Long Chain Polyunsaturated Fatty Acid (n3 and n6)	-0.065	6.34e-07
**112**	1-palmitoyl-2-docosahexaenoyl-GPC (16:0/22:6)	HMDB0007991	Lipid	Phosphatidylcholine (PC)	-0.064	6.36e-07
**113**	sebacate (C10-DC)	HMDB0000792	Lipid	Fatty Acid, Dicarboxylate	-0.070	7.01e-07
**114**	oleoylcarnitine (C18:1)	HMDB0005065	Lipid	Fatty Acid Metabolism (Acyl Carnitine, Monounsaturated)	-0.066	1.01e-06
**115**	(S)-3-hydroxybutyrylcarnitine	HMDB0013127	Lipid	Fatty Acid Metabolism (Acyl Carnitine, Hydroxy)	-0.068	1.15e-06
**116**	arachidonate (20:4n6)	HMDB0001043	Lipid	Long Chain Polyunsaturated Fatty Acid (n3 and n6)	-0.063	1.15e-06
**117**	1-myristoyl-2-arachidonoyl-GPC (14:0/20:4)*	HMDB0007883	Lipid	Phosphatidylcholine (PC)	0.064	1.19e-06
**118**	tetradecadienedioate (C14:2-DC)*	unknown	Lipid	Fatty Acid, Dicarboxylate	-0.068	1.79e-06
**119**	3-hydroxyoctanoylcarnitine (2)	unknown	Lipid	Fatty Acid Metabolism (Acyl Carnitine, Hydroxy)	-0.064	3.90e-06
**120**	2-aminobutyrate	HMDB0000452	Amino Acid	Glutathione Metabolism	-0.066	3.95e-06
**121**	sphingomyelin (d18:0/18:0, d19:0/17:0)*	HMDB0012087	Lipid	Dihydrosphingomyelins	-0.058	4.32e-06
**122**	linoleoyl ethanolamide	HMDB0012252	Lipid	Endocannabinoid	-0.065	6.02e-06

	CHEMICAL NAME	HMDB	CHEMICAL CLASS	SUB PATHWAY	β	P_{bonf}
123	cis-4-decenoylcarnitine (C10:1)	HMDB0013205	Lipid	Fatty Acid Metabolism (Acyl Carnitine, Monounsaturated)	-0.065	6.09e-06
124	3-hydroxyhexanoylcarnitine (1)	unknown	Lipid	Fatty Acid Metabolism (Acyl Carnitine, Hydroxy)	-0.065	7.38e-06
125	3-hydroxyoctanoylcarnitine (1)	unknown	Lipid	Fatty Acid Metabolism (Acyl Carnitine, Hydroxy)	-0.063	7.56e-06
126	xanthine	HMDB0000292	Nucleotide	Purine Metabolism, (Hypo)Xanthine/Inosine containing	-0.061	7.99e-06
127	2-hydroxypalmitate	HMDB0031057	Lipid	Fatty Acid, Monohydroxy	-0.059	8.17e-06
128	myo-inositol	HMDB0000211	Lipid	Inositol Metabolism	-0.065	9.74e-06
129	1,5-anhydroglucitol (1,5-AG)	HMDB0002712	Carbohydrate	Glycolysis, Gluconeogenesis, and Pyruvate Metabolism	0.061	9.93e-06
130	citramalate	HMDB0000426	Amino Acid	Glutamate Metabolism	-0.061	1.52e-05
131	adipoylcarnitine (C6-DC)	HMDB0061677	Lipid	Fatty Acid Metabolism (Acyl Carnitine, Dicarboxylate)	-0.064	1.52e-05
132	laurylcarnitine (C12)	HMDB000225	Lipid	Fatty Acid Metabolism (Acyl Carnitine, Medium Chain)	-0.062	2.79e-05
133	glutarylcarnitine (C5-DC)	HMDB0013130	Amino Acid	Lysine Metabolism	-0.061	2.80e-05
134	N-acetylarginine	HMDB0004620	Amino Acid	Urea cycle; Arginine and Proline Metabolism	0.060	3.33e-05
135	tridecenedioate (C13:1-DC)*	unknown	Lipid	Fatty Acid, Dicarboxylate	-0.061	4.67e-05
136	glutamine_degradant*	unknown	Partially Characterized Molecules	Partially Characterized Molecules	-0.051	5.18e-05
137	1-palmitoyl-2-stearoyl-GPC (16:0/18:0)	HMDB0007970	Lipid	Phosphatidylcholine (PC)	0.051	5.56e-05
138	N-acetylcarnosine	HMDB0012881	Amino Acid	Histidine Metabolism	-0.047	5.99e-05

	CHEMICAL NAME	HMDB	CHEMICAL CLASS	SUB PATHWAY	β	P_{bonf}
**139**	linoleoylcholine*	HMDB0013213	Lipid	Fatty Acid Metabolism (Acyl Choline)	0.063	8.95e-05
**140**	3-methyl-2-oxobutyrate	HMDB0000019	Amino Acid	Leucine, Isoleucine and Valine Metabolism	-0.055	9.05e-05
**141**	cholate	HMDB0000619	Lipid	Primary Bile Acid Metabolism	0.060	9.92e-05
**142**	1-(1-enyl-palmitoyl)-2-linoleoyl-GPC (P-16:0/18:2)*	HMDB0011211	Lipid	Plasmalogen	0.053	1.01e-04
**143**	N-lactoyl leucine	HMDB0062176	Amino Acid	Leucine, Isoleucine and Valine Metabolism	-0.054	1.19e-04
**144**	leucine	HMDB0000687	Amino Acid	Leucine, Isoleucine and Valine Metabolism	-0.048	1.27e-04
**145**	N-acetyl-aspartyl-glutamate (NAAG)	HMDB0001067	Amino Acid	Glutamate Metabolism	-0.058	1.64e-04
**146**	pimeloylcarnitine/3-methyladipoylcarnitine (C7-DC)	unknown	Lipid	Fatty Acid Metabolism (Acyl Carnitine, Dicarboxylate)	-0.057	1.65e-04
**147**	dodecanedioate (C12-DC)	HMDB0000623	Lipid	Fatty Acid, Dicarboxylate	-0.058	2.10e-04
**148**	fumarate	HMDB0000134	Energy	TCA Cycle	-0.058	2.35e-04
**149**	tetradecadienoate (14:2)*	HMDB0000560	Lipid	Long Chain Polyunsaturated Fatty Acid (n3 and n6)	-0.058	2.38e-04
**150**	glutamine conjugate of C6H10O2 (2)*	unknown	Partially Characterized Molecules	Partially Characterized Molecules	-0.058	2.54e-04
**151**	5,6-dihydrouridine	HMDB0000497	Nucleotide	Pyrimidine Metabolism, Uracil containing	-0.057	3.13e-04
**152**	eicosanedioate (C20-DC)	unknown	Lipid	Fatty Acid, Dicarboxylate	0.057	3.35e-04
**153**	androstenediol (3beta,17beta) disulfate (2)	HMDB0240313	Lipid	Androgenic Steroids	-0.051	3.42e-04
**154**	2,3-dihydroxy-2-methylbutyrate	HMDB0029576	Amino Acid	Leucine, Isoleucine and Valine Metabolism	-0.056	3.92e-04

	CHEMICAL NAME	HMDB	CHEMICAL CLASS	SUB PATHWAY	β	P_{bonf}
**155**	pregnenetriol disulfate*	unknown	Lipid	Pregnenolone Steroids	-0.051	4.62e-04
**156**	2R,3R-dihydroxybutyrate	HMDB0000498	Lipid	Fatty Acid, Dihydroxy	0.056	4.89e-04
**157**	3-hydroxybutyrate (BHBA)	HMDB0000442	Lipid	Ketone Bodies	-0.057	5.07e-04
**158**	13-HODE + 9-HODE	HMDB0004670	Lipid	Fatty Acid, Monohydroxy	-0.055	5.20e-04
**159**	1-myristoyl-2-palmitoyl-GPC (14:0/16:0)	HMDB0007869	Lipid	Phosphatidylcholine (PC)	0.055	6.47e-04
**160**	N-acetylglucosamine/N-acetylgalactosamine	HMDB0000212	Carbohydrate	Aminosugar Metabolism	-0.054	6.65e-04
**161**	1-linoleoyl-GPC (18:2)	HMDB0010386	Lipid	Lysophospholipid	0.049	7.16e-04
**162**	hexanoylcarnitine (C6)	HMDB0000756	Lipid	Fatty Acid Metabolism (Acyl Carnitine, Medium Chain)	-0.056	7.45e-04
**163**	3-hydroxy-3-methylglutarate	HMDB0000355	Lipid	Mevalonate Metabolism	-0.052	9.42e-04
**164**	pregnenediol disulfate (C21H34O8S2)*	unknown	Lipid	Pregnenolone Steroids	-0.050	9.63e-04
**165**	S-1-pyrroline-5-carboxylate	HMDB0001301	Amino Acid	Glutamate Metabolism	0.054	1.03e-03
**166**	alpha-ketoglutaramate*	HMDB0001552	Amino Acid	Glutamate Metabolism	-0.053	1.09e-03
**167**	beta-citrylglutamate	unknown	Amino Acid	Glutamate Metabolism	-0.054	1.14e-03
**168**	deoxycholic acid glucuronide	unknown	Lipid	Secondary Bile Acid Metabolism	0.054	1.14e-03
**169**	1-(1-enyl-palmitoyl)-GPC (P-16:0)*	HMDB0010407	Lipid	Lysoplasmalogen	0.048	1.17e-03
**170**	1-oleoyl-GPC (18:1)	HMDB0002815	Lipid	Lysophospholipid	0.049	1.27e-03
**171**	phosphate	HMDB0001429	Energy	Oxidative Phosphorylation	-0.050	1.29e-03
**172**	N-lactoyl phenylalanine	HMDB0062175	Amino Acid	Phenylalanine Metabolism	-0.050	1.35e-03
**173**	(N(1) + N(8))-acetylspermidine	HMDB0002189	Amino Acid	Polyamine Metabolism	-0.055	1.51e-03
**174**	1-palmitoleoyl-2-linolenoyl-GPC (16:1/18:3)*	HMDB0008008	Lipid	Phosphatidylcholine (PC)	0.050	1.64e-03

	CHEMICAL NAME	HMDB	CHEMICAL CLASS	SUB PATHWAY	β	P_{bonf}
**175**	1-(1-enyl-palmitoyl)-2-arachidonoyl-GPC (P-16:0/20:4)*	HMDB0011220	Lipid	Plasmalogen	0.051	1.95e-03
**176**	cholic acid glucuronide	HMDB0002577	Lipid	Primary Bile Acid Metabolism	0.057	2.01e-03
**177**	3-(3-amino-3-carboxypropyl)uridine*	unknown	Nucleotide	Pyrimidine Metabolism, Uracil containing	-0.051	2.07e-03
**178**	C-glycosyltryptophan	HMDB0240296	Amino Acid	Tryptophan Metabolism	-0.051	2.13e-03
**179**	epiandrosterone sulfate	HMDB0062657	Lipid	Androgenic Steroids	-0.049	2.28e-03
**180**	phenylalanine	HMDB0000159	Amino Acid	Phenylalanine Metabolism	-0.051	2.29e-03
**181**	N6-carbamoylthreonyladenosine	HMDB0041623	Nucleotide	Purine Metabolism, Adenine containing	-0.050	2.36e-03
**182**	10-undecenoate (11:1n1)	HMDB0033724	Lipid	Medium Chain Fatty Acid	-0.053	2.44e-03
**183**	androstenediol (3beta,17beta) monosulfate (1)	HMDB0240429	Lipid	Androgenic Steroids	-0.048	2.85e-03
**184**	linoleoylcarnitine (C18:2)*	HMDB0006469	Lipid	Fatty Acid Metabolism (Acyl Carnitine, Polyunsaturated)	-0.047	3.15e-03
**185**	indoleacetate	HMDB0000197	Amino Acid	Tryptophan Metabolism	-0.052	3.31e-03
**186**	androstenediol (3beta,17beta) disulfate (1)	HMDB0240313	Lipid	Androgenic Steroids	-0.050	3.43e-03
**187**	sphingomyelin (d18:1/17:0, d17:1/18:0, d19:1/16:0)	unknown	Lipid	Sphingomyelins	-0.046	3.55e-03
**188**	4-hydroxyphenylacetoylcarnitine	unknown	Amino Acid	Tyrosine Metabolism	-0.051	3.58e-03
**189**	5alpha-androstan-3beta,17beta-diol monosulfate (2)	unknown	Lipid	Androgenic Steroids	-0.048	3.82e-03
**190**	palmitoylcholine	HMDB0240592	Lipid	Fatty Acid Metabolism (Acyl Choline)	0.051	3.93e-03

	CHEMICAL NAME	HMDB	CHEMICAL CLASS	SUB PATHWAY	β	P_{bonf}
**191**	hexadecenedioate (C16:1-DC)*	unknown	Lipid	Fatty Acid, Dicarboxylate	-0.052	4.18e-03
**192**	taurolithocholate 3-sulfate	HMDB0002580	Lipid	Secondary Bile Acid Metabolism	-0.051	4.41e-03
**193**	N-oleoylserine	unknown	Lipid	Endocannabinoid	-0.051	4.46e-03
**194**	N-lactoyl tyrosine	HMDB0062177	Amino Acid	Tyrosine Metabolism	-0.047	5.51e-03
**195**	docosatrienoate (22:3n6)*	unknown	Lipid	Long Chain Polyunsaturated Fatty Acid (n3 and n6)	-0.053	5.79e-03
**196**	N-acetylserine	HMDB0002931	Amino Acid	Glycine, Serine and Threonine Metabolism	-0.050	7.14e-03
**197**	16a-hydroxy DHEA 3-sulfate	HMDB0062544	Lipid	Androgenic Steroids	-0.049	7.51e-03
**198**	palmitoylcarnitine (C16)	HMDB0000222	Lipid	Fatty Acid Metabolism (Acyl Carnitine, Long Chain Saturated)	-0.045	7.94e-03
**199**	imidazole lactate	HMDB0002320	Amino Acid	Histidine Metabolism	-0.049	8.32e-03
**200**	1-stearoyl-2-oleoyl-GPC (18:0/18:1)	HMDB0008038	Lipid	Phosphatidylcholine (PC)	0.046	8.48e-03
**201**	threonate	HMDB0062620	Cofactors and Vitamins	Ascorbate and Aldarate Metabolism	-0.049	8.57e-03
**202**	cortisol	HMDB0000063	Lipid	Corticosteroids	-0.049	9.09e-03
**203**	cytosine	HMDB0000630	Nucleotide	Pyrimidine Metabolism, Cytidine containing	0.049	9.32e-03
**204**	glycine conjugate of C10H14O2 (1)*	unknown	Partially Characterized Molecules	Partially Characterized Molecules	-0.049	1.02e-02
**205**	N-stearoyl-sphingosine (d18:1/18:0)*	HMDB0004950	Lipid	Ceramides	-0.043	1.03e-02
**206**	maleate	HMDB0000176	Lipid	Fatty Acid, Dicarboxylate	0.049	1.09e-02
**207**	guanidinoacetate	HMDB0000128	Amino Acid	Creatine Metabolism	-0.047	1.12e-02
**208**	indolelactate	HMDB0000671	Amino Acid	Tryptophan Metabolism	-0.046	1.14e-02
**209**	7-alpha-hydroxy-3-oxo-4-cholestenoate (7-Hoca)	HMDB0012458	Lipid	Sterol	-0.048	1.14e-02

	CHEMICAL NAME	HMDB	CHEMICAL CLASS	SUB PATHWAY	β	P_{bonf}
**210**	2-butenoylglycine	unknown	Lipid	Fatty Acid Metabolism (Acyl Glycine)	-0.047	1.23e-02
**211**	4-hydroxyphenylacetate	HMDB0000020	Amino Acid	Phenylalanine Metabolism	-0.050	1.31e-02
**212**	isoleucine	HMDB0000172	Amino Acid	Leucine, Isoleucine and Valine Metabolism	-0.039	1.37e-02
**213**	mannitol/sorbitol	HMDB0000247	Carbohydrate	Fructose, Mannose and Galactose Metabolism	0.049	1.47e-02
**214**	GlcNAc sulfate conjugate of C21H34O2 steroid**	unknown	Partially Characterized Molecules	Partially Characterized Molecules	-0.052	1.49e-02
**215**	citrate	HMDB0000094	Energy	TCA Cycle	-0.046	1.54e-02
**216**	pseudouridine	HMDB0000767	Nucleotide	Pyrimidine Metabolism, Uracil containing	-0.047	1.91e-02
**217**	glutamine conjugate of C7H12O2*	unknown	Partially Characterized Molecules	Partially Characterized Molecules	-0.048	1.97e-02
**218**	biliverdin	HMDB0001008	Cofactors and Vitamins	Hemoglobin and Porphyrin Metabolism	-0.046	2.04e-02
**219**	homocitrulline	HMDB0000679	Amino Acid	Urea cycle; Arginine and Proline Metabolism	-0.048	2.09e-02
**220**	lactosyl-N-palmitoyl-sphingosine (d18:1/16:0)	unknown	Lipid	Lactosylceramides (LCER)	-0.045	2.20e-02
**221**	chenodeoxycholate	HMDB0000518	Lipid	Primary Bile Acid Metabolism	0.048	2.23e-02
**222**	21-hydroxypregnenolone disulfate	unknown	Lipid	Pregnenolone Steroids	-0.045	2.24e-02
**223**	malonylcarnitine	HMDB0002095	Lipid	Fatty Acid Synthesis	-0.051	2.44e-02
**224**	beta-hydroxyisovalerate	HMDB0000754	Amino Acid	Leucine, Isoleucine and Valine Metabolism	-0.044	2.55e-02
**225**	N-formylmethionine	HMDB0001015	Amino Acid	Methionine, Cysteine, SAM and Taurine Metabolism	-0.047	2.69e-02
**226**	gamma-glutamylphenylalanine	HMDB0000594	Peptide	Gamma-glutamyl Amino Acid	-0.043	2.99e-02

	CHEMICAL NAME	HMDB	CHEMICAL CLASS	SUB PATHWAY	β	P_{bonf}
227	methionine	HMDB0000696	Amino Acid	Methionine, Cysteine, SAM and Taurine Metabolism	-0.044	3.16e-02
228	bilirubin (Z,Z)	HMDB0000054	Cofactors and Vitamins	Hemoglobin and Porphyrin Metabolism	-0.043	3.18e-02
229	gamma-glutamylleucine	HMDB0011171	Peptide	Gamma-glutamyl Amino Acid	-0.038	3.47e-02
230	3beta-hydroxy-5-cholestenoate	unknown	Lipid	Sterol	-0.041	3.48e-02
231	dehydroepiandrosterone sulfate (DHEA-S)	HMDB0001032	Lipid	Androgenic Steroids	-0.042	3.78e-02
232	branched-chain, straight-chain, or cyclopropyl 10:1 fatty acid (1)*	unknown	Partially Characterized Molecules	Partially Characterized Molecules	-0.043	5.06e-02
233	1-(1-enyl-oleoyl)-GPE (P-18:1)*	unknown	Lipid	Lysoplasmalogen	0.043	6.53e-02
234	5alpha-androstan-3beta,17beta-diol disulfate	HMDB00493	Lipid	Androgenic Steroids	-0.040	6.59e-02
235	5alpha-androstan-3alpha,17beta-diol disulfate	HMDB0094682	Lipid	Androgenic Steroids	-0.039	6.60e-02
236	N-acetylalanine	HMDB0000766	Amino Acid	Alanine and Aspartate Metabolism	-0.044	6.83e-02
237	pentose acid*	HMDB0000892	Partially Characterized Molecules	Partially Characterized Molecules	-0.044	9.31e-02
238	deoxycholate	HMDB0000626	Lipid	Secondary Bile Acid Metabolism	0.044	9.62e-02
239	tryptophan betaine	HMDB0061115	Amino Acid	Tryptophan Metabolism	-0.043	1.19e-01
240	ascorbic acid 3-sulfate*	unknown	Cofactors and Vitamins	Ascorbate and Aldarate Metabolism	-0.042	1.23e-01
241	dimethylarginine (SDMA + ADMA)	HMDB0003334	Amino Acid	Urea cycle; Arginine and Proline Metabolism	-0.042	1.26e-01
242	8-methoxykynurenate	HMDB0060426	Amino Acid	Tryptophan Metabolism	-0.042	1.28e-01
243	glycerophosphorylcholine (GPC)	HMDB0000086	Lipid	Phospholipid Metabolism	0.040	1.48e-01
244	octanoylcarnitine (C8)	HMDB0000791	Lipid	Fatty Acid Metabolism (Acyl Carnitine, Medium Chain)	-0.042	1.52e-01

	CHEMICAL NAME	HMDB	CHEMICAL CLASS	SUB PATHWAY	β	P_{bonf}
**245**	sphingomyelin (d18:1/20:2, d18:2/20:1, d16:1/22:2)*	unknown	Lipid	Sphingomyelins	-0.037	1.57e-01
**246**	eicosapentaenoate (EPA; 20:5n3)	HMDB0001999	Lipid	Long Chain Polyunsaturated Fatty Acid (n3 and n6)	-0.040	1.92e-01
**247**	1-stearoyl-2-oleoyl-GPI (18:0/18:1)*	unknown	Lipid	Phosphatidylinositol (PI)	0.038	1.96e-01
**248**	decanoylcarnitine (C10)	HMDB0000651	Lipid	Fatty Acid Metabolism (Acyl Carnitine, Medium Chain)	-0.041	2.01e-01
**249**	1-oleoyl-GPE (18:1)	HMDB0011506	Lipid	Lysophospholipid	0.040	2.02e-01
**250**	11beta-hydroxyetiocholanolone glucuronide*	unknown	Lipid	Androgenic Steroids	-0.041	2.05e-01
**251**	5-(galactosylhydroxy)-L-lysine	unknown	Amino Acid	Lysine Metabolism	-0.041	2.24e-01
**252**	N-acetylputrescine	HMDB0002064	Amino Acid	Polyamine Metabolism	-0.040	2.37e-01
**253**	creatine	HMDB0000064	Amino Acid	Creatine Metabolism	0.036	2.44e-01
**254**	gamma-glutamylthreonine	HMDB0029159	Peptide	Gamma-glutamyl Amino Acid	0.040	2.50e-01
**255**	histidine	HMDB0000177	Amino Acid	Histidine Metabolism	0.040	2.63e-01
**256**	N-acetylneuraminate	HMDB0000230	Carbohydrate	Aminosugar Metabolism	-0.039	3.37e-01
**257**	3-carboxy-4-methyl-5-pentyl-2-furanpropionate (3-CMPFP)**	HMDB0061643	Lipid	Fatty Acid, Dicarboxylate	-0.039	3.39e-01
**258**	2-hydroxysebacate	HMDB0000424	Lipid	Fatty Acid, Dicarboxylate	-0.042	3.41e-01
**259**	2-hydroxyglutarate	HMDB0059655	Lipid	Fatty Acid, Dicarboxylate	-0.038	3.57e-01
**260**	pregnenolone sulfate	HMDB0000774	Lipid	Pregnenolone Steroids	-0.037	3.59e-01
**261**	etiocholanolone glucuronide	HMDB0004484	Lipid	Androgenic Steroids	-0.039	3.64e-01
**262**	cis-4-decenoate (10:1n6)*	HMDB0004980	Lipid	Medium Chain Fatty Acid	-0.040	3.77e-01

	CHEMICAL NAME	HMDB	CHEMICAL CLASS	SUB PATHWAY	β	P_{bonf}
**263**	bilirubin degradation product, C16H18N2O5 (2)**	unknown	Partially Characterized Molecules	Partially Characterized Molecules	-0.038	3.94e-01
**264**	proline	HMDB0000162	Amino Acid	Urea cycle; Arginine and Proline Metabolism	0.038	4.05e-01
**265**	cystathionine	HMDB0000099	Amino Acid	Methionine, Cysteine, SAM and Taurine Metabolism	0.039	4.11e-01
**266**	sphingomyelin (d18:1/22:2, d18:2/22:1, d16:1/24:2)*	unknown	Lipid	Sphingomyelins	-0.033	4.12e-01
**267**	2-aminoheptanoate	HMDB0094649	Lipid	Fatty Acid, Amino	0.039	4.56e-01
**268**	N-formylphenylalanine	HMDB0240317	Amino Acid	Tyrosine Metabolism	-0.039	5.10e-01
**269**	4-methoxyphenol sulfate	unknown	Amino Acid	Tyrosine Metabolism	0.038	5.46e-01
**270**	gamma-glutamylhistidine	HMDB0029151	Peptide	Gamma-glutamyl Amino Acid	-0.036	5.60e-01
**271**	2-hydroxy-4-(methylthio)butanoic acid	HMDB0037115	Amino Acid	Methionine, Cysteine, SAM and Taurine Metabolism	-0.036	5.96e-01
**272**	N6,N6,N6-trimethyllysine	HMDB0001325	Amino Acid	Lysine Metabolism	0.037	6.10e-01
**273**	octadecanedioylcarnitine (C18-DC)*	unknown	Lipid	Fatty Acid Metabolism (Acyl Carnitine, Dicarboxylate)	-0.038	6.11e-01
**274**	N-stearoyl-sphinganine (d18:0/18:0)*	unknown	Lipid	Dihydroceramides	-0.038	6.26e-01
**275**	bilirubin degradation product, C17H20N2O5 (1)**	unknown	Partially Characterized Molecules	Partially Characterized Molecules	-0.037	6.91e-01
**276**	laurate (12:0)	HMDB0000638	Lipid	Medium Chain Fatty Acid	-0.037	6.92e-01
**277**	cysteinylglycine disulfide*	HMDB0000709	Amino Acid	Glutathione Metabolism	-0.033	6.95e-01
**278**	trigonelline (N'-methylnicotinate)	HMDB0000875	Cofactors and Vitamins	Nicotinate and Nicotinamide Metabolism	-0.037	7.53e-01
**279**	bilirubin degradation product, C16H18N2O5 (1)**	unknown	Partially Characterized Molecules	Partially Characterized Molecules	-0.036	7.83e-01

	CHEMICAL NAME	HMDB	CHEMICAL CLASS	SUB PATHWAY	β	P_{bonf}
280	tyrosine	HMDB0000158	Amino Acid	Tyrosine Metabolism	-0.033	7.83e-01
281	ethylmalonate	HMDB0000622	Amino Acid	Leucine, Isoleucine and Valine Metabolism	-0.038	7.89e-01
282	N-methylproline	HMDB0094696	Amino Acid	Urea cycle; Arginine and Proline Metabolism	-0.037	8.48e-01
283	methionine sulfone	HMDB0062174	Amino Acid	Methionine, Cysteine, SAM and Taurine Metabolism	-0.036	8.89e-01
284	butyrylcarnitine (C4)	HMDB0002013	Lipid	Fatty Acid Metabolism (also BCAA Metabolism)	-0.037	9.01e-01
285	N-alpha-acetylornithine	HMDB0003357	Amino Acid	Urea cycle; Arginine and Proline Metabolism	0.033	9.30e-01
286	gamma-glutamyl-2-aminobutyrate	unknown	Peptide	Gamma-glutamyl Amino Acid	-0.035	1.12e+00
287	bilirubin degradation product, C17H20N2O5 (2)**	unknown	Partially Characterized Molecules	Partially Characterized Molecules	-0.035	1.17e+00
288	docosadioate (C22-DC)	HMDB0061714	Lipid	Fatty Acid, Dicarboxylate	0.036	1.23e+00
289	glyco-beta-muricholate**	unknown	Lipid	Primary Bile Acid Metabolism	0.036	1.30e+00
290	lysine	HMDB0003405	Amino Acid	Lysine Metabolism	0.036	1.30e+00
291	isobutyrylcarnitine (C4)	HMDB0000736	Amino Acid	Leucine, Isoleucine and Valine Metabolism	0.036	1.36e+00
292	sphingomyelin (d18:2/18:1)*	HMDB0001348	Lipid	Sphingomyelins	-0.031	1.39e+00
293	bilirubin (E,E)*	unknown	Cofactors and Vitamins	Hemoglobin and Porphyrin Metabolism	-0.035	1.39e+00
294	5alpha-androstan-3alpha,17beta-diol monosulfate (1)	unknown	Lipid	Androgenic Steroids	-0.034	1.43e+00
295	1-arachidonoyl-GPC (20:4n6)*	HMDB0010395	Lipid	Lysophospholipid	0.032	1.50e+00
296	N-acetylcitrulline	HMDB0000856	Amino Acid	Urea cycle; Arginine and Proline Metabolism	0.035	1.50e+00

	CHEMICAL NAME	HMDB	CHEMICAL CLASS	SUB PATHWAY	β	P_{bonf}
**297**	glycolithocholate sulfate*	HMDB0002639	Lipid	Secondary Bile Acid Metabolism	-0.035	1.51e+00
**298**	sphingomyelin (d18:1/19:0, d19:1/18:0)*	unknown	Lipid	Sphingomyelins	-0.029	1.63e+00
**299**	N-stearoyl-sphingadienine (d18:2/18:0)*	unknown	Lipid	Ceramides	-0.034	1.64e+00
**300**	isovalerate (i5:0)	HMDB0000718	Amino Acid	Leucine, Isoleucine and Valine Metabolism	-0.034	1.69e+00
**301**	phosphoethanolamine	HMDB0000224	Lipid	Phospholipid Metabolism	-0.035	1.71e+00
**302**	succinylcarnitine (C4-DC)	HMDB0061717	Energy	TCA Cycle	0.033	1.77e+00
**303**	lactate	HMDB0000190	Carbohydrate	Glycolysis, Gluconeogenesis, and Pyruvate Metabolism	-0.033	1.82e+00
**304**	6-oxopiperidine-2-carboxylate	HMDB0061705	Amino Acid	Lysine Metabolism	0.034	1.82e+00
**305**	4-acetamidobutanoate	HMDB0003681	Amino Acid	Polyamine Metabolism	-0.034	1.87e+00
**306**	1-linoleoyl-GPE (18:2)*	HMDB0011507	Lipid	Lysophospholipid	0.033	1.94e+00
**307**	androsterone sulfate	HMDB0002759	Lipid	Androgenic Steroids	-0.033	1.95e+00
**308**	sphingomyelin (d18:1/25:0, d19:0/24:1, d20:1/23:0, d19:1/24:0)*	unknown	Lipid	Sphingomyelins	-0.031	1.95e+00
**309**	tetrahydrocortisol glucuronide	unknown	Lipid	Corticosteroids	-0.031	2.29e+00
**310**	cis-3,4-methyleneheptanoylcarnitine	unknown	Lipid	Fatty Acid Metabolism (Acyl Carnitine, Medium Chain)	0.033	2.32e+00
**311**	1-(1-enyl-stearoyl)-GPE (P-18:0)*	unknown	Lipid	Lysoplasmalogen	0.031	2.32e+00
**312**	sphinganine-1-phosphate	HMDB0001383	Lipid	Sphingolipid Synthesis	-0.034	2.39e+00
**313**	1-(1-enyl-palmitoyl)-2-oleoyl-GPC (P-16:0/18:1)*	HMDB0007996	Lipid	Plasmalogen	0.028	2.56e+00
**314**	4-hydroxyglutamate	HMDB0001344	Amino Acid	Glutamate Metabolism	0.032	2.56e+00

	CHEMICAL NAME	HMDB	CHEMICAL CLASS	SUB PATHWAY	β	P_{bonf}
**315**	1-palmitoyl-2-docosahexaenoyl-GPE (16:0/22:6)*	HMDB0008946	Lipid	Phosphatidylethanolamine (PE)	-0.031	2.87e+00
**316**	N-acetyl-2-aminoadipate	unknown	Amino Acid	Lysine Metabolism	0.032	2.92e+00
**317**	gamma-glutamylisoleucine*	HMDB0011170	Peptide	Gamma-glutamyl Amino Acid	-0.028	3.05e+00
**318**	nicotinamide riboside	HMDB0000855	Cofactors and Vitamins	Nicotinate and Nicotinamide Metabolism	-0.030	3.27e+00
**319**	2-O-methylascorbic acid	HMDB0240294	Cofactors and Vitamins	Ascorbate and Aldarate Metabolism	-0.030	3.33e+00
**320**	N-formylanthranilic acid	HMDB0004089	Amino Acid	Tryptophan Metabolism	-0.032	3.39e+00
**321**	sphingomyelin (d18:2/24:2)*	unknown	Lipid	Sphingomyelins	-0.027	3.60e+00
**322**	phenol sulfate	HMDB0060015	Amino Acid	Tyrosine Metabolism	0.031	3.93e+00
**323**	beta-cryptoxanthin	HMDB0033844	Cofactors and Vitamins	Vitamin A Metabolism	-0.030	4.18e+00
**324**	pregnenetriol sulfate*	unknown	Lipid	Pregnenolone Steroids	-0.027	4.35e+00
**325**	eicosenoylcarnitine (C20:1)*	unknown	Lipid	Fatty Acid Metabolism (Acyl Carnitine, Monounsaturated)	-0.031	4.36e+00
**326**	pro-hydroxy-pro	HMDB0006695	Amino Acid	Urea cycle; Arginine and Proline Metabolism	0.031	4.53e+00
**327**	branched chain 14:0 dicarboxylic acid**	unknown	Lipid	Fatty Acid, Dicarboxylate	-0.030	4.62e+00
**328**	3-aminoisobutyrate	HMDB0002166	Nucleotide	Pyrimidine Metabolism, Thymine containing	-0.031	4.69e+00
**329**	trans-urocanate	HMDB0000301	Amino Acid	Histidine Metabolism	0.030	4.78e+00
**330**	2'-O-methylcytidine	unknown	Nucleotide	Pyrimidine Metabolism, Cytidine containing	-0.031	4.78e+00
**331**	3-methoxytyrosine	HMDB0001434	Amino Acid	Tyrosine Metabolism	0.031	5.01e+00
**332**	1-(1-enyl-palmitoyl)-GPE (P-16:0)*	HMDB0011152	Lipid	Lysoplasmalogen	0.029	5.14e+00

	CHEMICAL NAME	HMDB	CHEMICAL CLASS	SUB PATHWAY	β	P_{bonf}
**333**	vanillactate	HMDB0000913	Amino Acid	Tyrosine Metabolism	0.031	5.30e+00
**334**	4-hydroxyphenylacetylglutamine	unknown	Peptide	Acetylated Peptides	-0.030	5.53e+00
**335**	dihydroorotate	HMDB03349	Nucleotide	Pyrimidine Metabolism, Orotate containing	0.030	5.59e+00
**336**	gamma-tocopherol/beta-tocopherol	HMDB0006335	Cofactors and Vitamins	Tocopherol Metabolism	0.030	5.62e+00
**337**	N-acetyl-isoputreanine	unknown	Amino Acid	Polyamine Metabolism	-0.030	5.67e+00
**338**	9,10-DiHOME	HMDB0004704	Lipid	Fatty Acid, Dihydroxy	-0.030	5.80e+00
**339**	bilirubin degradation product, C17H18N2O4 (3)**	unknown	Partially Characterized Molecules	Partially Characterized Molecules	-0.028	6.16e+00
**340**	pantothenate	HMDB0000210	Cofactors and Vitamins	Pantothenate and CoA Metabolism	-0.030	6.28e+00
**341**	hydroxy-N6,N6,N6-trimethyllysine*	unknown	Amino Acid	Lysine Metabolism	0.029	6.63e+00
**342**	5-methylthioadenosine (MTA)	HMDB0001173	Amino Acid	Polyamine Metabolism	-0.028	7.02e+00
**343**	sphingomyelin (d18:1/20:0, d16:1/22:0)*	HMDB0012102	Lipid	Sphingomyelins	-0.026	7.13e+00
**344**	inosine	HMDB0000195	Nucleotide	Purine Metabolism, (Hypo)Xanthine/Inosine containing	-0.029	7.68e+00
**345**	gamma-glutamylvaline	HMDB0011172	Peptide	Gamma-glutamyl Amino Acid	-0.025	7.69e+00
**346**	androstenediol (3beta,17beta) monosulfate (2)	HMDB0240429	Lipid	Androgenic Steroids	-0.029	7.75e+00
**347**	N-acetyltaurine	HMDB0240253	Amino Acid	Methionine, Cysteine, SAM and Taurine Metabolism	-0.028	8.11e+00
**348**	cys-gly, oxidized	unknown	Amino Acid	Glutathione Metabolism	-0.028	8.82e+00
**349**	1-lignoceroyl-GPC (24:0)	HMDB0010405	Lipid	Lysophospholipid	0.024	8.94e+00
**350**	hydroxyasparagine**	HMDB32332	Amino Acid	Alanine and Aspartate Metabolism	-0.026	9.03e+00

	CHEMICAL NAME	HMDB	CHEMICAL CLASS	SUB PATHWAY	β	P_{bonf}
**351**	fructose	HMDB0000660	Carbohydrate	Fructose, Mannose and Galactose Metabolism	0.028	9.76e+00
**352**	1-oleoyl-GPI (18:1)	HMDB0061693	Lipid	Lysophospholipid	0.027	9.89e+00
**353**	cis-3,4-methyleneheptanoate	unknown	Lipid	Fatty Acid, Branched	0.028	1.02e+01
**354**	2-oxoarginine*	HMDB0004225	Amino Acid	Urea cycle; Arginine and Proline Metabolism	0.026	1.04e+01
**355**	bilirubin degradation product, C17H18N2O4 (2)**	unknown	Partially Characterized Molecules	Partially Characterized Molecules	-0.026	1.08e+01
**356**	1-methyl-5-imidazolelactate	unknown	Amino Acid	Histidine Metabolism	-0.027	1.12e+01
**357**	glycerophosphoethanolamine	HMDB0000114	Lipid	Phospholipid Metabolism	0.026	1.13e+01
**358**	1-palmitoyl-GPC (16:0)	HMDB0010382	Lipid	Lysophospholipid	0.023	1.14e+01
**359**	methylsuccinoylcarnitine	unknown	Amino Acid	Leucine, Isoleucine and Valine Metabolism	-0.028	1.24e+01
**360**	5alpha-androstan-3beta,17alpha-diol disulfate	HMDB0094682	Lipid	Androgenic Steroids	-0.026	1.24e+01
**361**	5alpha-androstan-3alpha,17alpha-diol monosulfate	HMDB0000412	Lipid	Androgenic Steroids	-0.026	1.29e+01
**362**	androstenediol (3alpha, 17alpha) monosulfate (3)	unknown	Lipid	Androgenic Steroids	-0.022	1.32e+01
**363**	N,N,N-trimethyl-5-aminovalerate	unknown	Amino Acid	Lysine Metabolism	-0.026	1.40e+01
**364**	taurochenodeoxycholic acid 3-sulfate	HMDB0002486	Lipid	Secondary Bile Acid Metabolism	-0.027	1.43e+01
**365**	carnitine	HMDB0000062	Lipid	Carnitine Metabolism	0.026	1.44e+01
**366**	3,4-dihydroxybutyrate	HMDB0000337	Lipid	Fatty Acid, Dihydroxy	-0.026	1.51e+01
**367**	N6-methyllysine	HMDB0002038	Amino Acid	Lysine Metabolism	-0.026	1.52e+01
**368**	sphingomyelin (d18:0/20:0, d16:0/22:0)*	unknown	Lipid	Dihydrosphingomyelins	-0.023	1.52e+01
**369**	glycochenodeoxycholate 3-sulfate	HMDB0002409	Lipid	Primary Bile Acid Metabolism	-0.026	1.59e+01

	CHEMICAL NAME	HMDB	CHEMICAL CLASS	SUB PATHWAY	β	P_{bonf}
**370**	1-stearoyl-2-docosahexaenoyl-GPE (18:0/22:6)*	HMDB0009012	Lipid	Phosphatidylethanolamine (PE)	-0.024	1.63e+01
**371**	lignoceroyl sphingomyelin (d18:1/24:0)	unknown	Lipid	Sphingomyelins	-0.020	1.68e+01
**372**	pyruvate	HMDB0000243	Carbohydrate	Glycolysis, Gluconeogenesis, and Pyruvate Metabolism	-0.024	1.75e+01
**373**	dopamine 3-O-sulfate	HMDB0006275	Amino Acid	Tyrosine Metabolism	0.026	1.86e+01
**374**	N2-acetyl,N6,N6-dimethyllysine	unknown	Amino Acid	Lysine Metabolism	-0.025	1.87e+01
**375**	1-palmitoyl-2-arachidonoyl-GPE (16:0/20:4)*	HMDB0005323	Lipid	Phosphatidylethanolamine (PE)	-0.024	1.88e+01
**376**	alpha-ketoglutarate	HMDB0000208	Energy	TCA Cycle	-0.024	1.88e+01
**377**	N6,N6-dimethyllysine	HMDB0013287	Amino Acid	Lysine Metabolism	-0.025	1.91e+01
**378**	orotate	HMDB0000226	Nucleotide	Pyrimidine Metabolism, Orotate containing	0.023	1.94e+01
**379**	taurodeoxycholic acid 3-sulfate	unknown	Lipid	Secondary Bile Acid Metabolism	-0.025	1.99e+01
**380**	bilirubin (E,Z or Z,E)*	HMDB0000488	Cofactors and Vitamins	Hemoglobin and Porphyrin Metabolism	-0.024	2.03e+01
**381**	chiro-inositol	HMDB0240209	Lipid	Inositol Metabolism	-0.025	2.07e+01
**382**	pregnenediol sulfate (C21H34O5S)*	HMDB0000774	Lipid	Pregnenolone Steroids	-0.022	2.09e+01
**383**	kynurenate	HMDB0000715	Amino Acid	Tryptophan Metabolism	-0.021	2.09e+01
**384**	1-linoleoyl-GPG (18:2)*	unknown	Lipid	Lysophospholipid	-0.023	2.10e+01
**385**	1-ribosyl-imidazoleacetate*	HMDB0002331	Amino Acid	Histidine Metabolism	-0.025	2.11e+01
**386**	bilirubin degradation product, C17H18N2O4 (1)**	unknown	Partially Characterized Molecules	Partially Characterized Molecules	-0.024	2.19e+01
**387**	S-adenosylhomocysteine (SAH)	HMDB00939	Amino Acid	Methionine, Cysteine, SAM and Taurine Metabolism	-0.024	2.22e+01

	CHEMICAL NAME	HMDB	CHEMICAL CLASS	SUB PATHWAY	β	P_{bonf}
**388**	carboxyethyl-GABA	HMDB0002201	Amino Acid	Glutamate Metabolism	-0.024	2.31e+01
**389**	1-palmitoyl-2-arachidonoyl-GPI (16:0/20:4)*	HMDB0009789	Lipid	Phosphatidylinositol (PI)	0.023	2.36e+01
**390**	caprate (10:0)	HMDB0000511	Lipid	Medium Chain Fatty Acid	-0.024	2.53e+01
**391**	arabinose	HMDB0029942	Carbohydrate	Pentose Metabolism	0.026	2.56e+01
**392**	glutamine conjugate of C6H10O2 (1)*	unknown	Partially Characterized Molecules	Partially Characterized Molecules	-0.024	2.73e+01
**393**	N-acetylkynurenine (2)	unknown	Amino Acid	Tryptophan Metabolism	0.022	2.75e+01
**394**	succinate	HMDB0000254	Energy	TCA Cycle	-0.021	2.83e+01
**395**	pyridoxate	HMDB0000017	Cofactors and Vitamins	Vitamin B6 Metabolism	-0.024	2.88e+01
**396**	1-stearoyl-2-oleoyl-GPE (18:0/18:1)	HMDB0008993	Lipid	Phosphatidylethanolamine (PE)	0.022	2.92e+01
**397**	cortolone glucuronide (1)	unknown	Lipid	Corticosteroids	0.020	3.16e+01
**398**	glycodeoxycholate 3-sulfate	unknown	Lipid	Secondary Bile Acid Metabolism	-0.023	3.24e+01
**399**	17alpha-hydroxypregnenolone 3-sulfate	HMDB00416	Lipid	Pregnenolone Steroids	-0.022	3.25e+01
**400**	glycerate	HMDB0000139	Carbohydrate	Glycolysis, Gluconeogenesis, and Pyruvate Metabolism	-0.021	3.31e+01
**401**	tricosanoyl sphingomyelin (d18:1/23:0)*	HMDB0012105	Lipid	Sphingomyelins	-0.016	3.32e+01
**402**	1-palmitoyl-2-dihomo-linolenoyl-GPC (16:0/20:3n3 or 6)*	unknown	Lipid	Phosphatidylcholine (PC)	0.021	3.32e+01
**403**	sarcosine	HMDB0000271	Amino Acid	Glycine, Serine and Threonine Metabolism	0.020	3.35e+01
**404**	N1-methylinosine	HMDB0002721	Nucleotide	Purine Metabolism, (Hypo)Xanthine/Inosine containing	-0.020	3.44e+01
**405**	riboflavin (Vitamin B2)	HMDB0000244	Cofactors and Vitamins	Riboflavin Metabolism	-0.023	3.47e+01

	CHEMICAL NAME	HMDB	CHEMICAL CLASS	SUB PATHWAY	β	P_{bonf}
**406**	glycosyl-N-behenoyl-sphingadienine (d18:2/22:0)*	unknown	Lipid	Hexosylceramides (HCER)	0.020	3.56e+01
**407**	alanine	HMDB0000161	Amino Acid	Alanine and Aspartate Metabolism	-0.022	3.59e+01
**408**	2,3-dihydroxy-5-methylthio-4-pentenoate (DMTPA)*	HMDB0240388	Amino Acid	Methionine, Cysteine, SAM and Taurine Metabolism	-0.020	3.65e+01
**409**	2-hydroxydecanoate	HMDB0094656	Lipid	Fatty Acid, Monohydroxy	-0.022	3.72e+01
**410**	ursodeoxycholate	HMDB0000946	Lipid	Secondary Bile Acid Metabolism	0.022	3.73e+01
**411**	2'-deoxyuridine	HMDB0000012	Nucleotide	Pyrimidine Metabolism, Uracil containing	0.020	3.89e+01
**412**	methylsuccinate	HMDB0001844	Amino Acid	Leucine, Isoleucine and Valine Metabolism	-0.022	3.98e+01
**413**	oleoyl-linoleoyl-glycerol (18:1/18:2) [1]	HMDB0007219	Lipid	Diacylglycerol	-0.021	4.00e+01
**414**	phenylacetylglutamate	HMDB0059772	Peptide	Acetylated Peptides	-0.022	4.27e+01
**415**	cis-3,4-methyleneheptanoylglycine	unknown	Lipid	Fatty Acid Metabolism (Acyl Glycine)	-0.021	4.39e+01
**416**	sphingomyelin (d17:1/14:0, d16:1/15:0)*	unknown	Lipid	Sphingomyelins	0.017	4.43e+01
**417**	citrulline	HMDB0000904	Amino Acid	Urea cycle; Arginine and Proline Metabolism	0.021	4.44e+01
**418**	oleoyl-linoleoyl-glycerol (18:1/18:2) [2]	HMDB0007219	Lipid	Diacylglycerol	-0.020	4.53e+01
**419**	N2,N2-dimethylguanosine	HMDB0004824	Nucleotide	Purine Metabolism, Guanine containing	-0.017	4.59e+01
**420**	2-ketocaprylate	HMDB13211	Amino Acid	Leucine, Isoleucine and Valine Metabolism	-0.021	4.73e+01
**421**	6-bromotryptophan	unknown	Amino Acid	Tryptophan Metabolism	0.020	4.77e+01
**422**	mannose	HMDB0000169	Carbohydrate	Fructose, Mannose and Galactose Metabolism	-0.018	4.79e+01
**423**	caprylate (8:0)	HMDB0000482	Lipid	Medium Chain Fatty Acid	-0.021	4.87e+01

	CHEMICAL NAME	HMDB	CHEMICAL CLASS	SUB PATHWAY	β	P_{bonf}
424	cysteine	HMDB0000574	Amino Acid	Methionine, Cysteine, SAM and Taurine Metabolism	-0.021	4.88e+01
425	oxalate (ethanedioate)	HMDB0002329	Cofactors and Vitamins	Ascorbate and Aldarate Metabolism	-0.018	5.02e+01
426	4-guanidinobutanoate	HMDB0003464	Amino Acid	Guanidino and Acetamido Metabolism	0.020	5.04e+01
427	gamma-CEHC	HMDB0001931	Cofactors and Vitamins	Tocopherol Metabolism	0.021	5.04e+01
428	3-carboxy-4-methyl-5-propyl-2-furanpropanoate (CMPF)	HMDB0061112	Lipid	Fatty Acid, Dicarboxylate	-0.020	5.10e+01
429	1-linolenoylglycerol (18:3)	HMDB0011569	Lipid	Monoacylglycerol	0.020	5.18e+01
430	1-palmitoyl-2-linoleoyl-GPE (16:0/18:2)	HMDB0005322	Lipid	Phosphatidylethanolamine (PE)	-0.020	5.23e+01
431	1-palmitoyl-2-arachidonoyl-GPC (16:0/20:4n6)	HMDB0007982	Lipid	Phosphatidylcholine (PC)	-0.018	5.54e+01
432	indoleacetylglutamine	HMDB0013240	Amino Acid	Tryptophan Metabolism	-0.019	5.63e+01
433	picolinoylglycine	HMDB0059766	Lipid	Fatty Acid Metabolism (Acyl Glycine)	-0.020	5.79e+01
434	methionine sulfoxide	HMDB0002005	Amino Acid	Methionine, Cysteine, SAM and Taurine Metabolism	-0.017	6.00e+01
435	margaroylcarnitine (C17)*	HMDB0006210	Lipid	Fatty Acid Metabolism (Acyl Carnitine, Long Chain Saturated)	-0.019	6.07e+01
436	behenoyl dihydrosphingomyelin (d18:0/22:0)*	HMDB0012091	Lipid	Dihydrosphingomyelins	-0.017	6.17e+01
437	sphinganine	HMDB0000269	Lipid	Sphingolipid Synthesis	-0.019	6.27e+01
438	gamma-glutamylglutamine	HMDB0011738	Peptide	Gamma-glutamyl Amino Acid	0.007	6.29e+01
439	1-methyl-4-imidazoleacetate	HMDB0002820	Amino Acid	Histidine Metabolism	0.019	6.32e+01
440	2-hydroxyoctanoate	HMDB0002264	Lipid	Fatty Acid, Monohydroxy	-0.019	6.35e+01

	CHEMICAL NAME	HMDB	CHEMICAL CLASS	SUB PATHWAY	β	P_{bonf}
**441**	nervonoylcarnitine (C24:1)*	unknown	Lipid	Fatty Acid Metabolism (Acyl Carnitine, Monounsaturated)	0.020	6.53e+01
**442**	picolinate	HMDB0002243	Amino Acid	Tryptophan Metabolism	-0.019	6.60e+01
**443**	1-stearoyl-2-arachidonoyl-GPC (18:0/20:4)	HMDB0008048	Lipid	Phosphatidylcholine (PC)	0.017	6.71e+01
**444**	cytidine	HMDB0000089	Nucleotide	Pyrimidine Metabolism, Cytidine containing	-0.020	6.92e+01
**445**	1-arachidonoyl-GPE (20:4n6)*	HMDB0011517	Lipid	Lysophospholipid	0.018	6.97e+01
**446**	undecenoylcarnitine (C11:1)	unknown	Lipid	Fatty Acid Metabolism (Acyl Carnitine, Monounsaturated)	-0.018	7.01e+01
**447**	imidazole propionate	HMDB0002271	Amino Acid	Histidine Metabolism	0.019	7.02e+01
**448**	nonanoylcarnitine (C9)	HMDB0013288	Lipid	Fatty Acid Metabolism (Acyl Carnitine, Medium Chain)	-0.019	7.02e+01
**449**	1-stearoyl-2-docosahexaenoyl-GPC (18:0/22:6)	HMDB0008057	Lipid	Phosphatidylcholine (PC)	-0.018	7.10e+01
**450**	sphingomyelin (d18:2/23:1)*	unknown	Lipid	Sphingomyelins	-0.015	7.22e+01
**451**	argininate*	HMDB0003148	Amino Acid	Urea cycle; Arginine and Proline Metabolism	0.019	7.22e+01
**452**	N-acetylvaline	HMDB0011757	Amino Acid	Leucine, Isoleucine and Valine Metabolism	-0.013	7.23e+01
**453**	erucate (22:1n9)	HMDB0002068	Lipid	Long Chain Monounsaturated Fatty Acid	-0.018	7.26e+01
**454**	N-palmitoyl-sphinganine (d18:0/16:0)	HMDB11760	Lipid	Dihydroceramides	0.016	7.37e+01
**455**	1-palmitoyl-GPI (16:0)	HMDB0061695	Lipid	Lysophospholipid	0.018	7.52e+01
**456**	glutarate (C5-DC)	HMDB0000661	Lipid	Fatty Acid, Dicarboxylate	0.018	7.68e+01

	CHEMICAL NAME	HMDB	CHEMICAL CLASS	SUB PATHWAY	β	P_{bonf}
**457**	myristoyl dihydrosphingomyelin (d18:0/14:0)*	HMDB0012085	Lipid	Dihydrosphingomyelins	0.016	7.92e+01
**458**	1-stearoyl-2-linoleoyl-GPI (18:0/18:2)	HMDB0009809	Lipid	Phosphatidylinositol (PI)	0.016	8.03e+01
**459**	sphingomyelin (d18:1/20:1, d18:2/20:0)*	unknown	Lipid	Sphingomyelins	-0.016	8.18e+01
**460**	1-palmitoyl-2-oleoyl-GPC (16:0/18:1)	HMDB0007972	Lipid	Phosphatidylcholine (PC)	-0.016	8.35e+01
**461**	carotene diol (3)	unknown	Cofactors and Vitamins	Vitamin A Metabolism	-0.017	8.35e+01
**462**	1-linoleoyl-GPI (18:2)*	unknown	Lipid	Lysophospholipid	-0.017	8.58e+01
**463**	homoarginine	HMDB0000670	Amino Acid	Urea cycle; Arginine and Proline Metabolism	-0.017	8.66e+01
**464**	butyrate/isobutyrate (4:0)	HMDB0000039	Lipid	Short Chain Fatty Acid	0.018	8.73e+01
**465**	azelate (C9-DC)	HMDB0000784	Lipid	Fatty Acid, Dicarboxylate	-0.018	8.73e+01
**466**	N-acetylaspartate (NAA)	HMDB0000812	Amino Acid	Alanine and Aspartate Metabolism	0.016	9.25e+01
**467**	5alpha-androstan-3alpha,17beta-diol monosulfate (2)	unknown	Lipid	Androgenic Steroids	-0.015	9.44e+01
**468**	3-methylglutarylcarnitine (2)	HMDB0000552	Amino Acid	Leucine, Isoleucine and Valine Metabolism	-0.016	9.67e+01
**469**	androstenediol (3alpha, 17alpha) monosulfate (2)	unknown	Lipid	Androgenic Steroids	-0.016	9.92e+01
**470**	palmitoyl-sphingosine-phosphoethanolamine (d18:1/16:0)	unknown	Lipid	Ceramide PEs	0.014	1.00e+02
**471**	N-acetylhistidine	HMDB0032055	Amino Acid	Histidine Metabolism	-0.017	1.01e+02
**472**	gamma-glutamylglutamate	HMDB0011737	Peptide	Gamma-glutamyl Amino Acid	-0.016	1.03e+02
**473**	1-stearoyl-2-oleoyl-GPS (18:0/18:1)	HMDB0010163	Lipid	Phosphatidylserine (PS)	-0.019	1.03e+02
**474**	glycocholenate sulfate*	unknown	Lipid	Secondary Bile Acid Metabolism	-0.016	1.04e+02
**475**	cholesterol	HMDB0000067	Lipid	Sterol	-0.010	1.05e+02

	CHEMICAL NAME	HMDB	CHEMICAL CLASS	SUB PATHWAY	β	P_{bonf}
**476**	glycodeoxycholate	HMDB00631	Lipid	Secondary Bile Acid Metabolism	0.017	1.06e+02
**477**	N2,N5-diacetylornithine	unknown	Amino Acid	Urea cycle; Arginine and Proline Metabolism	-0.016	1.06e+02
**478**	5-oxoproline	HMDB0000267	Amino Acid	Glutathione Metabolism	0.016	1.08e+02
**479**	pantoate	unknown	Cofactors and Vitamins	Pantothenate and CoA Metabolism	-0.017	1.08e+02
**480**	1-stearoyl-GPG (18:0)	unknown	Lipid	Lysophospholipid	-0.014	1.10e+02
**481**	caproate (6:0)	HMDB0000535	Lipid	Medium Chain Fatty Acid	-0.016	1.10e+02
**482**	N-lactoyl valine	HMDB0062181	Amino Acid	Leucine, Isoleucine and Valine Metabolism	-0.015	1.12e+02
**483**	arachidonoylcarnitine (C20:4)	HMDB0006455	Lipid	Fatty Acid Metabolism (Acyl Carnitine, Polyunsaturated)	-0.015	1.12e+02
**484**	xylose	HMDB0000098	Carbohydrate	Pentose Metabolism	0.016	1.14e+02
**485**	glycosyl-N-palmitoyl-sphingosine (d18:1/16:0)	unknown	Lipid	Hexosylceramides (HCER)	-0.014	1.23e+02
**486**	phenylacetylglutamine	HMDB0006344	Peptide	Acetylated Peptides	-0.015	1.24e+02
**487**	1-stearoyl-2-arachidonoyl-GPE (18:0/20:4)	HMDB0009003	Lipid	Phosphatidylethanolamine (PE)	-0.014	1.24e+02
**488**	1-dihomo-linolenylglycerol (20:3)	unknown	Lipid	Monoacylglycerol	-0.015	1.26e+02
**489**	lignoceroylcarnitine (C24)*	unknown	Lipid	Fatty Acid Metabolism (Acyl Carnitine, Long Chain Saturated)	0.015	1.27e+02
**490**	behenoylcarnitine (C22)*	HMDB0062468	Lipid	Fatty Acid Metabolism (Acyl Carnitine, Long Chain Saturated)	0.015	1.30e+02
**491**	behenoyl sphingomyelin (d18:1/22:0)*	HMDB0012103	Lipid	Sphingomyelins	-0.011	1.34e+02
**492**	1-palmitoyl-2-oleoyl-GPI (16:0/18:1)*	HMDB0009783	Lipid	Phosphatidylinositol (PI)	0.014	1.35e+02

	CHEMICAL NAME	HMDB	CHEMICAL CLASS	SUB PATHWAY	β	P_{bonf}
**493**	gamma-glutamylmethionine	HMDB0029155	Peptide	Gamma-glutamyl Amino Acid	-0.011	1.38e+02
**494**	glucose	HMDB0000122	Carbohydrate	Glycolysis, Gluconeogenesis, and Pyruvate Metabolism	-0.012	1.41e+02
**495**	1-stearoyl-GPE (18:0)	HMDB0011130	Lipid	Lysophospholipid	0.013	1.41e+02
**496**	N-acetylglucosaminylasparagine	HMDB0000489	Carbohydrate	Aminosugar Metabolism	-0.014	1.42e+02
**497**	1-palmitoyl-2-linoleoyl-GPI (16:0/18:2)	HMDB0009784	Lipid	Phosphatidylinositol (PI)	0.014	1.42e+02
**498**	2-palmitoyl-GPC (16:0)*	HMDB0061702	Lipid	Lysophospholipid	-0.013	1.43e+02
**499**	stearidonate (18:4n3)	HMDB0006547	Lipid	Long Chain Polyunsaturated Fatty Acid (n3 and n6)	-0.014	1.51e+02
**500**	1-arachidonylglycerol (20:4)	HMDB11578	Lipid	Monoacylglycerol	-0.014	1.52e+02
**501**	glycolithocholate	HMDB00698	Lipid	Secondary Bile Acid Metabolism	0.014	1.53e+02
**502**	deoxycarnitine	HMDB0001161	Lipid	Carnitine Metabolism	0.013	1.54e+02
**503**	sphingomyelin (d18:2/16:0, d18:1/16:1)*	unknown	Lipid	Sphingomyelins	-0.011	1.54e+02
**504**	1,2-dipalmitoyl-GPC (16:0/16:0)	HMDB0000564	Lipid	Phosphatidylcholine (PC)	0.013	1.54e+02
**505**	ascorbic acid 2-sulfate	HMDB0060649	Cofactors and Vitamins	Ascorbate and Aldarate Metabolism	-0.014	1.55e+02
**506**	glycosyl ceramide (d18:1/20:0, d16:1/22:0)*	unknown	Lipid	Hexosylceramides (HCER)	0.012	1.57e+02
**507**	1-methylhistidine	HMDB0000001	Amino Acid	Histidine Metabolism	-0.013	1.60e+02
**508**	N-acetyltryptophan	HMDB0013713	Amino Acid	Tryptophan Metabolism	-0.013	1.60e+02
**509**	lyxonate	unknown	Carbohydrate	Pentose Metabolism	-0.014	1.63e+02
**510**	stearoylcarnitine (C18)	HMDB0000848	Lipid	Fatty Acid Metabolism (Acyl Carnitine, Long Chain Saturated)	-0.012	1.65e+02
**511**	glycine	HMDB0000123	Amino Acid	Glycine, Serine and Threonine Metabolism	0.012	1.67e+02

	CHEMICAL NAME	HMDB	CHEMICAL CLASS	SUB PATHWAY	β	P_{bonf}
**512**	1-palmitoyl-2-palmitoleoyl-GPC (16:0/16:1)*	HMDB0007969	Lipid	Phosphatidylcholine (PC)	-0.013	1.72e+02
**513**	N-methyltaurine	unknown	Amino Acid	Methionine, Cysteine, SAM and Taurine Metabolism	0.014	1.73e+02
**514**	1-methyl-5-imidazoleacetate	HMDB04988	Amino Acid	Histidine Metabolism	-0.013	1.73e+02
**515**	ceramide (d18:2/24:1, d18:1/24:2)*	unknown	Lipid	Ceramides	0.013	1.73e+02
**516**	sphingomyelin (d17:1/16:0, d18:1/15:0, d16:1/17:0)*	unknown	Lipid	Sphingomyelins	-0.011	1.74e+02
**517**	3-methoxytyramine sulfate	unknown	Amino Acid	Tyrosine Metabolism	0.014	1.78e+02
**518**	cysteinylglycine	HMDB0000078	Amino Acid	Glutathione Metabolism	-0.012	1.82e+02
**519**	arginine	HMDB0000517	Amino Acid	Urea cycle; Arginine and Proline Metabolism	0.013	1.86e+02
**520**	quinolinate	HMDB0000232	Cofactors and Vitamins	Nicotinate and Nicotinamide Metabolism	0.012	1.93e+02
**521**	tryptophan	HMDB0000929	Amino Acid	Tryptophan Metabolism	-0.012	1.94e+02
**522**	carotene diol (2)	unknown	Cofactors and Vitamins	Vitamin A Metabolism	-0.012	1.96e+02
**523**	p-cresol glucuronide*	HMDB0011686	Amino Acid	Tyrosine Metabolism	-0.012	1.96e+02
**524**	oxindolylalanine	unknown	Amino Acid	Tryptophan Metabolism	-0.012	1.97e+02
**525**	glycerol 3-phosphate	HMDB0000126	Lipid	Glycerolipid Metabolism	-0.011	1.99e+02
**526**	N1-methyladenosine	HMDB0003331	Nucleotide	Purine Metabolism, Adenine containing	0.011	2.01e+02
**527**	sphingomyelin (d18:1/21:0, d17:1/22:0, d16:1/23:0)*	unknown	Lipid	Sphingomyelins	-0.010	2.03e+02
**528**	N-acetyltyrosine	HMDB0000866	Amino Acid	Tyrosine Metabolism	-0.011	2.08e+02
**529**	3-methylglutaconate	HMDB0000522	Amino Acid	Leucine, Isoleucine and Valine Metabolism	-0.011	2.10e+02

	CHEMICAL NAME	HMDB	CHEMICAL CLASS	SUB PATHWAY	β	P_{bonf}
**530**	1-oleoyl-2-linoleoyl-GPE (18:1/18:2)*	HMDB0005349	Lipid	Phosphatidylethanolamine (PE)	0.012	2.13e+02
**531**	2-hydroxystearate	HMDB0062549	Lipid	Fatty Acid, Monohydroxy	-0.011	2.15e+02
**532**	lactosyl-N-nervonoyl-sphingosine (d18:1/24:1)*	unknown	Lipid	Lactosylceramides (LCER)	-0.011	2.19e+02
**533**	glucuronate	HMDB0000127	Carbohydrate	Aminosugar Metabolism	-0.011	2.22e+02
**534**	hypotaurine	HMDB0000965	Amino Acid	Methionine, Cysteine, SAM and Taurine Metabolism	-0.011	2.24e+02
**535**	choline phosphate	HMDB0001565	Lipid	Phospholipid Metabolism	-0.011	2.24e+02
**536**	sphingomyelin (d18:2/21:0, d16:2/23:0)*	unknown	Lipid	Sphingomyelins	-0.009	2.26e+02
**537**	betaine	HMDB0000043	Amino Acid	Glycine, Serine and Threonine Metabolism	0.011	2.28e+02
**538**	7-methylguanine	HMDB0000897	Nucleotide	Purine Metabolism, Guanine containing	-0.011	2.30e+02
**539**	2-aminooctanoate	HMDB0000991	Lipid	Fatty Acid, Amino	-0.011	2.32e+02
**540**	3-indoxyl sulfate	HMDB0000682	Amino Acid	Tryptophan Metabolism	0.011	2.34e+02
**541**	isovalerylcarnitine (C5)	HMDB0000688	Amino Acid	Leucine, Isoleucine and Valine Metabolism	-0.011	2.35e+02
**542**	vanillic alcohol sulfate	HMDB0041788	Amino Acid	Tyrosine Metabolism	0.011	2.35e+02
**543**	11beta-hydroxyandrosterone glucuronide	unknown	Lipid	Androgenic Steroids	0.010	2.46e+02
**544**	3-phosphoglycerate	HMDB0000807	Carbohydrate	Glycolysis, Gluconeogenesis, and Pyruvate Metabolism	-0.010	2.50e+02
**545**	2'-O-methyluridine	unknown	Nucleotide	Pyrimidine Metabolism, Uracil containing	-0.010	2.60e+02
**546**	4-hydroxy-2-oxoglutaric acid	HMDB0002070	Lipid	Fatty Acid, Dicarboxylate	0.010	2.62e+02
**547**	1-arachidonoyl-GPI (20:4)*	HMDB0061690	Lipid	Lysophospholipid	-0.010	2.64e+02

	CHEMICAL NAME	HMDB	CHEMICAL CLASS	SUB PATHWAY	β	P_{bonf}
548	tetrahydrocortisone glucuronide (5)	unknown	Lipid	Corticosteroids	-0.009	2.64e+02
549	carnosine	HMDB0000033	Amino Acid	Histidine Metabolism	0.010	2.72e+02
550	trimethylamine N-oxide	HMDB0000925	Lipid	Phospholipid Metabolism	0.010	2.73e+02
551	carotene diol (1)	unknown	Cofactors and Vitamins	Vitamin A Metabolism	-0.009	2.74e+02
552	3beta,7alpha-dihydroxy-5-cholestenoate	HMDB0012454	Lipid	Sterol	-0.010	2.75e+02
553	propionylcarnitine (C3)	HMDB0000824	Lipid	Fatty Acid Metabolism (also BCAA Metabolism)	-0.009	2.77e+02
554	ximenoylcarnitine (C26:1)*	unknown	Lipid	Fatty Acid Metabolism (Acyl Carnitine, Monounsaturated)	0.009	2.78e+02
555	9-hydroxystearate	HMDB0061661	Lipid	Fatty Acid, Monohydroxy	-0.009	2.79e+02
556	cerotoylcarnitine (C26)*	HMDB0006347	Lipid	Fatty Acid Metabolism (Acyl Carnitine, Long Chain Saturated)	-0.008	2.80e+02
557	N-acetyl-beta-alanine	HMDB0061880	Nucleotide	Pyrimidine Metabolism, Uracil containing	-0.007	2.83e+02
558	sphingosine	HMDB0000252	Lipid	Sphingosines	-0.009	2.86e+02
559	N-acetylasparagine	HMDB0006028	Amino Acid	Alanine and Aspartate Metabolism	0.010	2.89e+02
560	3-ureidopropionate	HMDB0000026	Nucleotide	Pyrimidine Metabolism, Uracil containing	-0.010	2.89e+02
561	gamma-glutamyl-alpha-lysine	unknown	Peptide	Gamma-glutamyl Amino Acid	0.008	2.90e+02
562	glutamine	HMDB0000641	Amino Acid	Glutamate Metabolism	-0.008	2.93e+02
563	N1-Methyl-2-pyridone-5-carboxamide	HMDB0004193	Cofactors and Vitamins	Nicotinate and Nicotinamide Metabolism	-0.009	2.93e+02
564	glycoursodeoxycholate	HMDB0000708	Lipid	Secondary Bile Acid Metabolism	0.009	2.97e+02
565	gamma-glutamylglycine	HMDB0011667	Peptide	Gamma-glutamyl Amino Acid	-0.008	3.04e+02

	CHEMICAL NAME	HMDB	CHEMICAL CLASS	SUB PATHWAY	β	P_{bonf}
566	lithocholate sulfate (1)	unknown	Lipid	Secondary Bile Acid Metabolism	0.009	3.11e+02
567	isoleucylglycine	HMDB0028907	Peptide	Dipeptide	-0.009	3.17e+02
568	thymine	HMDB0000262	Nucleotide	Pyrimidine Metabolism, Thymine containing	-0.010	3.18e+02
569	glycosyl-N-stearoyl-sphingosine (d18:1/18:0)	unknown	Lipid	Hexosylceramides (HCER)	-0.008	3.18e+02
570	beta-hydroxyisovaleroylcarnitine	unknown	Amino Acid	Leucine, Isoleucine and Valine Metabolism	-0.009	3.19e+02
571	2-stearoyl-GPE (18:0)*	HMDB0011129	Lipid	Lysophospholipid	-0.008	3.20e+02
572	1-stearoyl-2-arachidonoyl-GPI (18:0/20:4)	HMDB0009815	Lipid	Phosphatidylinositol (PI)	-0.008	3.24e+02
573	N-delta-acetylornithine	HMDB0003357	Amino Acid	Urea cycle; Arginine and Proline Metabolism	-0.008	3.27e+02
574	1-palmitoleoyl-GPC (16:1)*	HMDB0010383	Lipid	Lysophospholipid	0.008	3.29e+02
575	4-oxo-retinoic acid	HMDB0006285	Cofactors and Vitamins	Vitamin A Metabolism	0.009	3.33e+02
576	isoursodeoxycholate	HMDB0000686	Lipid	Secondary Bile Acid Metabolism	0.008	3.33e+02
577	2-aminoadipate	HMDB0000510	Amino Acid	Lysine Metabolism	-0.008	3.34e+02
578	taurine	HMDB0000251	Amino Acid	Methionine, Cysteine, SAM and Taurine Metabolism	-0.008	3.36e+02
579	5-hydroxylysine	HMDB0000450	Amino Acid	Lysine Metabolism	0.008	3.36e+02
580	isobutyrylglycine	HMDB0000730	Amino Acid	Leucine, Isoleucine and Valine Metabolism	-0.008	3.37e+02
581	N-acetyl-1-methylhistidine*	unknown	Amino Acid	Histidine Metabolism	-0.008	3.38e+02
582	palmitoyl sphingomyelin (d18:1/16:0)	HMDB0010169	Lipid	Sphingomyelins	-0.006	3.39e+02
583	propionylglycine	HMDB0000783	Lipid	Fatty Acid Metabolism (also BCAA Metabolism)	0.008	3.43e+02

	CHEMICAL NAME	HMDB	CHEMICAL CLASS	SUB PATHWAY	β	P_{bonf}
584	1-(1-enyl-palmitoyl)-2-palmitoleoyl-GPC (P-16:0/16:1)*	HMDB0011207	Lipid	Plasmalogen	0.007	3.44e+02
585	sphingomyelin (d18:2/23:0, d18:1/23:1, d17:1/24:1)*	unknown	Lipid	Sphingomyelins	-0.006	3.49e+02
586	N2-acetyl,N6-methyllysine	unknown	Amino Acid	Lysine Metabolism	-0.008	3.52e+02
587	tauroursodeoxycholate	HMDB0000874	Lipid	Secondary Bile Acid Metabolism	-0.009	3.53e+02
588	dihomo-linoleoylcarnitine (C20:2)*	unknown	Lipid	Fatty Acid Metabolism (Acyl Carnitine, Polyunsaturated)	-0.007	3.57e+02
589	gulonate*	HMDB0003290	Cofactors and Vitamins	Ascorbate and Aldarate Metabolism	-0.008	3.59e+02
590	gamma-glutamylcitrulline*	unknown	Peptide	Gamma-glutamyl Amino Acid	-0.005	3.59e+02
591	kynurenine	HMDB0000684	Amino Acid	Tryptophan Metabolism	0.007	3.59e+02
592	arabitol/xylitol	HMDB0001851	Carbohydrate	Pentose Metabolism	-0.008	3.60e+02
593	N-palmitoyl-sphingadienine (d18:2/16:0)*	unknown	Lipid	Ceramides	-0.007	3.61e+02
594	S-methylcysteine sulfoxide	HMDB0029432	Amino Acid	Methionine, Cysteine, SAM and Taurine Metabolism	-0.007	3.69e+02
595	N-acetylleucine	HMDB0011756	Amino Acid	Leucine, Isoleucine and Valine Metabolism	-0.006	3.70e+02
596	5-hydroxyindole sulfate	unknown	Amino Acid	Tryptophan Metabolism	0.008	3.70e+02
597	3-methylhistidine	HMDB0000479	Amino Acid	Histidine Metabolism	-0.007	3.71e+02
598	4-hydroxyphenylpyruvate	HMDB0000707	Amino Acid	Tyrosine Metabolism	-0.007	3.73e+02
599	1-stearoyl-GPI (18:0)	HMDB0240261	Lipid	Lysophospholipid	-0.007	3.78e+02
600	tyramine O-sulfate	HMDB0006409	Amino Acid	Tyrosine Metabolism	0.007	3.78e+02
601	lactosyl-N-behenoyl-sphingosine (d18:1/22:0)*	HMDB0011594	Lipid	Lactosylceramides (LCER)	-0.007	3.80e+02

	CHEMICAL NAME	HMDB	CHEMICAL CLASS	SUB PATHWAY	β	P_{bonf}
**602**	5alpha-pregnan-3beta,20alpha-diol disulfate	HMDB0094650	Lipid	Progestin Steroids	-0.007	3.81e+02
**603**	uridine	HMDB0000296	Nucleotide	Pyrimidine Metabolism, Uracil containing	-0.007	3.81e+02
**604**	N6-methyladenosine	HMDB0004044	Nucleotide	Purine Metabolism, Adenine containing	0.007	3.85e+02
**605**	1-methylnicotinamide	HMDB0000699	Cofactors and Vitamins	Nicotinate and Nicotinamide Metabolism	-0.007	3.85e+02
**606**	N-acetylphenylalanine	HMDB0000512	Amino Acid	Phenylalanine Metabolism	-0.007	3.97e+02
**607**	ribitol	HMDB0002917	Carbohydrate	Pentose Metabolism	-0.007	3.97e+02
**608**	phenylpyruvate	HMDB0000205	Amino Acid	Phenylalanine Metabolism	-0.005	4.03e+02
**609**	1-palmitoylglycerol (16:0)	HMDB0011564	Lipid	Monoacylglycerol	0.007	4.04e+02
**610**	dihomo-linolenoylcarnitine (C20:3n3 or 6)*	unknown	Lipid	Fatty Acid Metabolism (Acyl Carnitine, Polyunsaturated)	0.006	4.05e+02
**611**	1-(1-enyl-palmitoyl)-2-palmitoyl-GPC (P-16:0/16:0)*	HMDB0011206	Lipid	Plasmalogen	0.006	4.07e+02
**612**	N-acetyl-3-methylhistidine*	unknown	Amino Acid	Histidine Metabolism	-0.006	4.10e+02
**613**	ceramide (d18:1/14:0, d16:1/16:0)*	unknown	Lipid	Ceramides	-0.006	4.14e+02
**614**	N,N-dimethylalanine	unknown	Amino Acid	Alanine and Aspartate Metabolism	-0.006	4.20e+02
**615**	2-methylserine	unknown	Amino Acid	Glycine, Serine and Threonine Metabolism	0.006	4.26e+02
**616**	beta-alanine	HMDB0000056	Nucleotide	Pyrimidine Metabolism, Uracil containing	0.006	4.27e+02
**617**	3b-hydroxy-5-cholenoic acid	HMDB0000308	Lipid	Secondary Bile Acid Metabolism	0.006	4.30e+02
**618**	1-oleoylglycerol (18:1)	HMDB0011567	Lipid	Monoacylglycerol	-0.006	4.31e+02

	CHEMICAL NAME	HMDB	CHEMICAL CLASS	SUB PATHWAY	β	P_{bonf}
**619**	urea	HMDB0000294	Amino Acid	Urea cycle; Arginine and Proline Metabolism	0.006	4.33e+02
**620**	2-linoleoylglycerol (18:2)	HMDB0011538	Lipid	Monoacylglycerol	0.006	4.38e+02
**621**	linolenoylcarnitine (C18:3)*	unknown	Lipid	Fatty Acid Metabolism (Acyl Carnitine, Polyunsaturated)	-0.006	4.43e+02
**622**	1-linoleoylglycerol (18:2)	HMDB0011568	Lipid	Monoacylglycerol	-0.006	4.44e+02
**623**	cysteine sulfinic acid	HMDB0000996	Amino Acid	Methionine, Cysteine, SAM and Taurine Metabolism	0.005	4.45e+02
**624**	glycochenodeoxycholate	HMDB0000637	Lipid	Primary Bile Acid Metabolism	-0.006	4.46e+02
**625**	Fibrinopeptide A, des-ala(1)*	unknown	Peptide	Fibrinogen Cleavage Peptide	0.006	4.49e+02
**626**	uracil	HMDB0000300	Nucleotide	Pyrimidine Metabolism, Uracil containing	-0.005	4.50e+02
**627**	N6-acetyllysine	HMDB0000206	Amino Acid	Lysine Metabolism	-0.005	4.51e+02
**628**	Fibrinopeptide A*	unknown	Peptide	Fibrinogen Cleavage Peptide	0.005	4.60e+02
**629**	valine	HMDB0000883	Amino Acid	Leucine, Isoleucine and Valine Metabolism	-0.005	4.60e+02
**630**	cysteine-glutathione disulfide	HMDB0000656	Amino Acid	Glutathione Metabolism	-0.006	4.61e+02
**631**	1-palmitoyl-GPE (16:0)	HMDB0011503	Lipid	Lysophospholipid	0.005	4.71e+02
**632**	4-imidazoleacetate	HMDB0002024	Amino Acid	Histidine Metabolism	0.005	4.72e+02
**633**	spermidine	HMDB0001257	Amino Acid	Polyamine Metabolism	-0.005	4.74e+02
**634**	asparagine	HMDB0000168	Amino Acid	Alanine and Aspartate Metabolism	-0.003	4.85e+02
**635**	palmitoyl dihydrosphingomyelin (d18:0/16:0)*	HMDB0010168	Lipid	Dihydrosphingomyelins	0.004	4.92e+02
**636**	choline	HMDB0000097	Lipid	Phospholipid Metabolism	0.004	5.00e+02

	CHEMICAL NAME	HMDB	CHEMICAL CLASS	SUB PATHWAY	β	P_{bonf}
**637**	lanthionine	unknown	Amino Acid	Methionine, Cysteine, SAM and Taurine Metabolism	0.004	5.01e+02
**638**	arachidoylcarnitine (C20)*	HMDB0006460	Lipid	Fatty Acid Metabolism (Acyl Carnitine, Long Chain Saturated)	-0.004	5.02e+02
**639**	1-oleoyl-2-arachidonoyl-GPE (18:1/20:4)*	HMDB0009069	Lipid	Phosphatidylethanolamine (PE)	-0.004	5.06e+02
**640**	5alpha-pregnan-3beta,20alpha-diol monosulfate (2)	unknown	Lipid	Progestin Steroids	-0.004	5.10e+02
**641**	1-palmitoyl-2-linoleoyl-GPC (16:0/18:2)	HMDB0007973	Lipid	Phosphatidylcholine (PC)	-0.004	5.17e+02
**642**	metabolonic lactone sulfate	unknown	Partially Characterized Molecules	Partially Characterized Molecules	0.004	5.17e+02
**643**	glutamate	HMDB0000148	Amino Acid	Glutamate Metabolism	-0.004	5.21e+02
**644**	sphingomyelin (d18:1/14:0, d16:1/16:0)*	HMDB0012097	Lipid	Sphingomyelins	-0.003	5.22e+02
**645**	1-palmitoyl-2-oleoyl-GPE (16:0/18:1)	HMDB0005320	Lipid	Phosphatidylethanolamine (PE)	0.004	5.23e+02
**646**	xanthurenate	HMDB0000881	Amino Acid	Tryptophan Metabolism	-0.004	5.25e+02
**647**	androsterone glucuronide	HMDB0002829	Lipid	Androgenic Steroids	0.003	5.29e+02
**648**	phenylacetate	HMDB0000209	Amino Acid	Phenylalanine Metabolism	0.004	5.30e+02
**649**	retinol (Vitamin A)	HMDB0000305	Cofactors and Vitamins	Vitamin A Metabolism	-0.003	5.31e+02
**650**	1-stearoyl-2-linoleoyl-GPE (18:0/18:2)*	HMDB0008994	Lipid	Phosphatidylethanolamine (PE)	0.004	5.32e+02
**651**	glucuronide of C10H18O2 (8)*	unknown	Partially Characterized Molecules	Partially Characterized Molecules	-0.004	5.34e+02
**652**	leucylglycine	HMDB0028929	Peptide	Dipeptide	-0.003	5.34e+02
**653**	ribonate	HMDB0000867	Carbohydrate	Pentose Metabolism	-0.003	5.37e+02
**654**	deoxycholic acid 12-sulfate*	unknown	Lipid	Secondary Bile Acid Metabolism	0.003	5.38e+02
**655**	gamma-glutamyltyrosine	HMDB0011741	Peptide	Gamma-glutamyl Amino Acid	-0.003	5.39e+02

	CHEMICAL NAME	HMDB	CHEMICAL CLASS	SUB PATHWAY	β	P_{bonf}
**656**	formiminoglutamate	HMDB0000854	Amino Acid	Histidine Metabolism	-0.003	5.40e+02
**657**	glucuronide of C10H18O2 (7)*	unknown	Partially Characterized Molecules	Partially Characterized Molecules	-0.003	5.43e+02
**658**	serine	HMDB0000187	Amino Acid	Glycine, Serine and Threonine Metabolism	0.003	5.45e+02
**659**	thyroxine	HMDB0000248	Amino Acid	Tyrosine Metabolism	-0.003	5.45e+02
**660**	glycosyl ceramide (d18:2/24:1, d18:1/24:2)*	unknown	Lipid	Hexosylceramides (HCER)	-0.003	5.48e+02
**661**	dimethylglycine	HMDB0000092	Amino Acid	Glycine, Serine and Threonine Metabolism	0.003	5.51e+02
**662**	fructosyllysine	HMDB0034879	Amino Acid	Lysine Metabolism	0.003	5.53e+02
**663**	indolepropionate	HMDB0002302	Amino Acid	Tryptophan Metabolism	0.003	5.55e+02
**664**	hydroxy-CMPF*	unknown	Lipid	Fatty Acid, Dicarboxylate	0.003	5.61e+02
**665**	sphingomyelin (d18:1/24:1, d18:2/24:0)*	HMDB0012107	Lipid	Sphingomyelins	0.002	5.70e+02
**666**	adenosine 5'-monophosphate (AMP)	HMDB0000045	Nucleotide	Purine Metabolism, Adenine containing	-0.003	5.73e+02
**667**	galactonate	HMDB0000565	Carbohydrate	Fructose, Mannose and Galactose Metabolism	0.003	5.76e+02
**668**	N-acetyl-2-aminooctanoate*	HMDB0059745	Lipid	Fatty Acid, Amino	-0.003	5.78e+02
**669**	sphingomyelin (d18:1/22:1, d18:2/22:0, d16:1/24:1)*	HMDB0012104	Lipid	Sphingomyelins	-0.002	5.86e+02
**670**	glycocholate	HMDB0000138	Lipid	Primary Bile Acid Metabolism	0.003	5.87e+02
**671**	S-methylcysteine	HMDB0002108	Amino Acid	Methionine, Cysteine, SAM and Taurine Metabolism	-0.003	5.87e+02
**672**	N-acetylglutamine	HMDB0006029	Amino Acid	Glutamate Metabolism	-0.003	5.89e+02
**673**	glycohyocholate	HMDB0000138	Lipid	Secondary Bile Acid Metabolism	-0.002	5.90e+02

	CHEMICAL NAME	HMDB	CHEMICAL CLASS	SUB PATHWAY	β	P_{bonf}
**674**	5alpha-pregnan-3beta,20beta-diol monosulfate (1)	HMDB0240580	Lipid	Progestin Steroids	0.002	5.94e+02
**675**	tiglylcarnitine (C5:1-DC)	HMDB0002366	Amino Acid	Leucine, Isoleucine and Valine Metabolism	-0.002	5.95e+02
**676**	hydantoin-5-propionate	HMDB0001212	Amino Acid	Histidine Metabolism	0.002	6.03e+02
**677**	5-methyluridine (ribothymidine)	HMDB0000884	Nucleotide	Pyrimidine Metabolism, Uracil containing	-0.002	6.10e+02
**678**	2-oleoylglycerol (18:1)	HMDB0011537	Lipid	Monoacylglycerol	0.002	6.10e+02
**679**	octadecadienedioate (C18:2-DC)*	unknown	Lipid	Fatty Acid, Dicarboxylate	-0.002	6.15e+02
**680**	N-acetylmethionine	HMDB0011745	Amino Acid	Methionine, Cysteine, SAM and Taurine Metabolism	-0.002	6.16e+02
**681**	1-palmitoleoylglycerol (16:1)*	unknown	Lipid	Monoacylglycerol	-0.002	6.17e+02
**682**	taurochenodeoxycholate	HMDB0000951	Lipid	Primary Bile Acid Metabolism	-0.002	6.17e+02
**683**	isovalerylglycine	HMDB00678	Amino Acid	Leucine, Isoleucine and Valine Metabolism	0.002	6.18e+02
**684**	heptanoate (7:0)	HMDB0000666	Lipid	Medium Chain Fatty Acid	0.002	6.20e+02
**685**	glucuronide of C10H18O2 (1)*	unknown	Partially Characterized Molecules	Partially Characterized Molecules	-0.002	6.21e+02
**686**	N-palmitoyl-sphingosine (d18:1/16:0)	HMDB0004949	Lipid	Ceramides	-0.001	6.24e+02
**687**	sphingosine 1-phosphate	HMDB0000277	Lipid	Sphingosines	0.002	6.25e+02
**688**	1-oleoyl-2-docosahexaenoyl-GPC (18:1/22:6)*	HMDB0008123	Lipid	Phosphatidylcholine (PC)	0.002	6.25e+02
**689**	allantoin	HMDB0000462	Nucleotide	Purine Metabolism, (Hypo)Xanthine/Inosine containing	0.002	6.27e+02
**690**	sphingomyelin (d17:2/16:0, d18:2/15:0)*	unknown	Lipid	Sphingomyelins	-0.001	6.28e+02

	CHEMICAL NAME	HMDB	CHEMICAL CLASS	SUB PATHWAY	β	P_{bonf}
**691**	cysteine s-sulfate	HMDB0000731	Amino Acid	Methionine, Cysteine, SAM and Taurine Metabolism	0.001	6.30e+02
**692**	oleoyl-arachidonoyl-glycerol (18:1/20:4) [2]*	HMDB0007228	Lipid	Diacylglycerol	-0.002	6.31e+02
**693**	alpha-tocopherol	HMDB0001893	Cofactors and Vitamins	Tocopherol Metabolism	-0.001	6.32e+02
**694**	sphingomyelin (d18:2/24:1, d18:1/24:2)*	unknown	Lipid	Sphingomyelins	0.001	6.38e+02
**695**	prolylglycine	HMDB0011178	Peptide	Dipeptide	-0.002	6.38e+02
**696**	Fibrinopeptide A, phosphono-ser(3)*	unknown	Peptide	Fibrinogen Cleavage Peptide	0.001	6.44e+02
**697**	pipecolate	unknown	Amino Acid	Lysine Metabolism	0.001	6.49e+02
**698**	1-palmitoyl-GPG (16:0)*	unknown	Lipid	Lysophospholipid	0.001	6.51e+02
**699**	aspartate	HMDB0000191	Amino Acid	Alanine and Aspartate Metabolism	-0.001	6.51e+02
**700**	linoleoyl-arachidonoyl-glycerol (18:2/20:4) [2]*	HMDB0007257	Lipid	Diacylglycerol	0.001	6.55e+02
**701**	nisinate (24:6n3)	HMDB0013025	Lipid	Long Chain Polyunsaturated Fatty Acid (n3 and n6)	-0.001	6.58e+02
**702**	N-lactoyl isoleucine	HMDB0062180	Amino Acid	Leucine, Isoleucine and Valine Metabolism	0.001	6.58e+02
**703**	sphingomyelin (d18:2/14:0, d18:1/14:1)*	unknown	Lipid	Sphingomyelins	-0.001	6.60e+02
**704**	glycochenodeoxycholate glucuronide (1)	HMDB0002579	Lipid	Primary Bile Acid Metabolism	-0.001	6.81e+02
**705**	taurodeoxycholate	HMDB0000896	Lipid	Secondary Bile Acid Metabolism	0.001	6.82e+02
**706**	phenylacetylcarnitine	unknown	Peptide	Acetylated Peptides	-0.001	6.86e+02
**707**	N-acetylglutamate	HMDB0001138	Amino Acid	Glutamate Metabolism	0.000	6.88e+02
**708**	glycoursodeoxycholic acid sulfate (1)	unknown	Lipid	Secondary Bile Acid Metabolism	0.000	6.91e+02
**709**	hydroxypalmitoyl sphingomyelin (d18:1/16:0(OH))**	unknown	Lipid	Sphingomyelins	0.000	6.96e+02

	CHEMICAL NAME	HMDB	CHEMICAL CLASS	SUB PATHWAY	β	P_{bonf}
**710**	nicotinamide	HMDB0001406	Cofactors and Vitamins	Nicotinate and Nicotinamide Metabolism	0.000	7.01e+02
**711**	5alpha-pregnan-diol disulfate	HMDB0240581	Lipid	Progestin Steroids	0.000	7.05e+02
**712**	arabonate/xylonate	HMDB0000539	Carbohydrate	Pentose Metabolism	0.000	7.06e+02
**713**	orotidine	HMDB0000788	Nucleotide	Pyrimidine Metabolism, Orotate containing	0.000	7.07e+02
