# Supplementary material for: The metabolic signature of salt intake: a cross-sectional analysis from the SCAPIS-study
Source: Nutr Metab (Lond). 2025 Sep 2;22:104. doi: 10.1186/s12986-025-00997-y (PMC12406461; doi:10.1186/s12986-025-00997-y)
Supplement: Supplementary file 3 — Supplementary Material 3 [file 12986_2025_997_MOESM3_ESM.pdf]

Supplementary data 4

Original results adjusted for total red meat (processed and unprocessed), poultry and macronutrients (fat, protein, carbohydrates) in g/day. Data from questionnaires.

1. Table 1 – Association between PC1 and CC
2. Figure 1 – Restricted cubic spline for energy CC
3. Table 2 – Associated energy metabolites
4. Figure 2 – Restricted cubic spline for lipid CC
5. Table 3 – Top ten associated lipid metabolites
6. Table 4 – Significant associated metabolites

Table 1: *Association between first principal components of Chemical Classes and est24hNa with Bonferroni-corrected p-values and p for non-linearity (ANOVA)*

Chemical Class	p_{bonf}	$p_{non\text{-}linearity}$
**Amino Acids**	1	0.066
**Carbohydrates**	0.890	0.055
**Cofactors and vitamins**	1	0.918
**Energy**	<0.0001	<0.0001
**Lipids**	<0.0001	<0.0001
**Nucleotides**	0.304	0.0172
**Peptides**	1	0.135
**Partially Characterized Molecules**	0.008	0.491

Figure 1: *Restricted cubic spline for the association between est24hNa and PC1 for energy (p < 0.0001,* $p_{non\text{-}linear}$ *= 0.0001)*

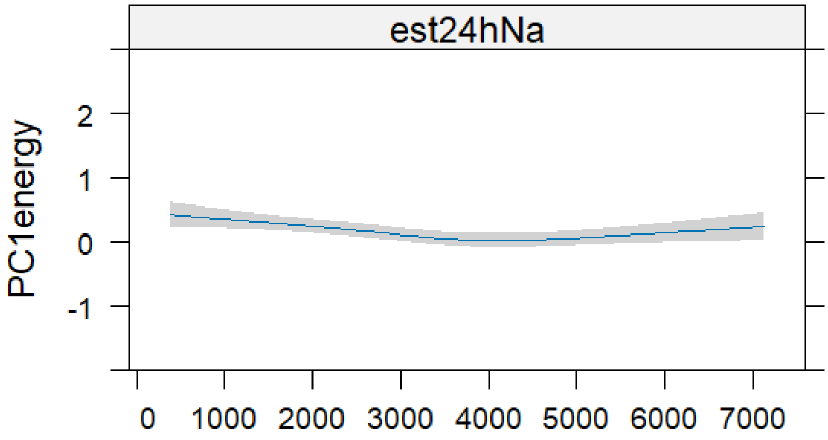

Table 2. *Energy metabolites with Bonferroni-corrected p-values.*

	Metabolite	HMDB	β	p_{bonf}
**1.**	citraconate/glutaconate	HMDB0000634	0.069	9.96×10^{-13}
**2.**	aconitate [cis or trans]	HMDB0000958	-0.064	1.73×10^{-12}
**3.**	malate	HMDB0031518	-0.060	2.44×10^{-10}
**4.**	fumarate	HMDB0000134	-0.046	5.26×10^{-6}
**5.**	phosphate	HMDB0001429	-0.039	4.31×10^{-5}
**6.**	citrate	HMDB0000094	-0.036	3.72×10^{-4}
**7.**	succinylcarnitine (C4-DC)	HMDB0061717	0.028	0.014
**8.**	alpha-ketoglutarate	HMDB0000208	-0.022	0.127
**9.**	succinate	HMDB0000254	-0.018	0.28

Figure 2: *Restricted cubic spline for the association between est24hNa and PC1 for lipids*

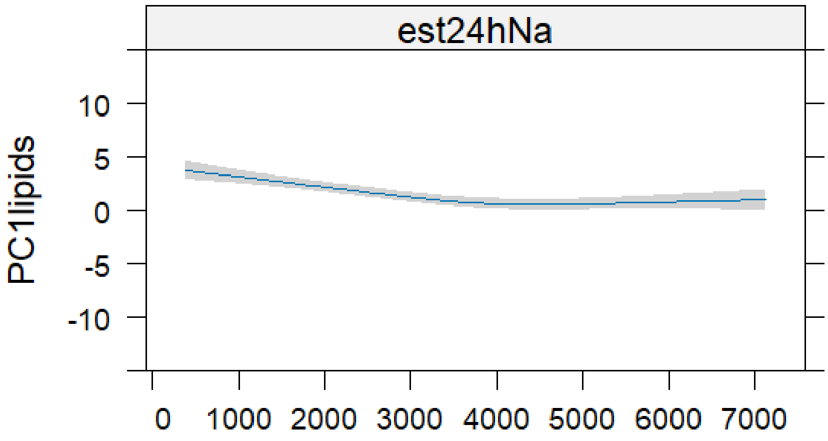

Table 3. *The 10 highest ranked lipid metabolites with Bonferroni-corrected p-values.*

	Metabolite	HMDB	β	p_{bonf}
**1.**	2S,3R-dihydroxybutyrate	HMDB0002453	-0.11	2.97×10^{-38}
**2.**	palmitate (16:0)	HMDB0000220	-0.10	2.08×10^{-29}
**3.**	oleate/vaccenate (18:1)	HMDB0003231	-0.10	2.30×10^{-28}
**4.**	acetylcarnitine (C2)	HMDB0000201	-0.10	6.19×10^{-27}
**5.**	10-heptadecenoate (17:1n7)	HMDB0060038	-0.094	2.98×10^{-24}
**6.**	margarate (17:0)	HMDB0002259	-0.092	2.98×10^{-24}
**7.**	dihomo-linoleate (20:2n6)	HMDB0005060	-0.093	4.48×10^{-24}
**8.**	linoleate	HMDB0006270	-0.091	5.46×10^{-24}
**9.**	eicosenoate (20:1)	HMDB0002231	-0.094	5.75×10^{-24}
**10.**	10-nonadecenoate (19:1n9)	HMDB0013622	-0.092	8.62×10^{-24}

Table 4. *Metabolites with significant associations to est24hNa in meat and macronutrient adjusted model. Estimates and bonferroni-corrected p-values.*

	CHEMICAL NAME	HMDB	CHEMICAL CLASS	SUB PATHWAY	β	p_{bonf}
1	2S,3R-dihydroxybutyrate	HMDB0002453	Lipid	Fatty Acid, Dihydroxy	-0.133	6.34e-38
2	homovanillate (HVA)	HMDB0000118	Amino Acid	Tyrosine Metabolism	-0.144	1.72e-35
3	N,N,N-trimethyl-alanylproline betaine (TMAP)	HMDB0240365	Amino Acid	Urea cycle; Arginine and Proline Metabolism	-0.124	5.43e-30
4	palmitate (16:0)	HMDB0000220	Lipid	Long Chain Saturated Fatty Acid	-0.125	9.70e-29
5	oleate/vaccenate (18:1)	HMDB0003231	Lipid	Long Chain Monounsaturated Fatty Acid	-0.124	8.99e-28
6	N,N-dimethyl-pro-pro	unknown	Peptide	Modified Peptides	-0.118	3.81e-27
7	2-hydroxyphenylacetate	HMDB0000669	Amino Acid	Phenylalanine Metabolism	-0.125	5.04e-27
8	acetylcarnitine (C2)	HMDB0000201	Lipid	Fatty Acid Metabolism (Acyl Carnitine, Short Chain)	-0.127	1.69e-26
9	10-heptadecenoate (17:1n7)	HMDB0060038	Lipid	Long Chain Monounsaturated Fatty Acid	-0.115	8.54e-25
10	creatinine	HMDB0000562	Amino Acid	Creatine Metabolism	-0.102	1.32e-24
11	alpha-hydroxyisocaproate	HMDB0000665	Amino Acid	Leucine, Isoleucine and Valine Metabolism	-0.107	1.37e-23
12	margarate (17:0)	HMDB0002259	Lipid	Long Chain Saturated Fatty Acid	-0.112	1.61e-23
13	linoleate (18:2n6)	HMDB0006270	Lipid	Long Chain Polyunsaturated Fatty Acid (n3 and n6)	-0.112	1.61e-23
14	dihomo-linoleate (20:2n6)	HMDB0005060	Lipid	Long Chain Polyunsaturated Fatty Acid (n3 and n6)	-0.114	2.02e-23
15	eicosenoate (20:1)	HMDB0002231	Lipid	Long Chain Monounsaturated Fatty Acid	-0.115	2.21e-23
16	10-nonadecenoate (19:1n9)	HMDB0013622	Lipid	Long Chain Monounsaturated Fatty Acid	-0.113	3.76e-23
17	hexadecanedioate (C16-DC)	HMDB0000672	Lipid	Fatty Acid, Dicarboxylate	-0.117	6.27e-22
18	3-hydroxy-2-ethylpropionate	HMDB0000396	Amino Acid	Leucine, Isoleucine and Valine Metabolism	-0.114	1.03e-21
19	palmitoleate (16:1n7)	HMDB0003229	Lipid	Long Chain Monounsaturated Fatty Acid	-0.108	1.08e-21
20	decadienedioic acid (C10:2-DC)**	unknown	Lipid	Fatty Acid, Dicarboxylate	-0.114	2.14e-21
21	(14 or 15)-methylpalmitate (a17:0 or i17:0)	HMDB0061859	Lipid	Fatty Acid, Branched	-0.105	4.25e-21
22	myristate (14:0)	HMDB0000806	Lipid	Long Chain Saturated Fatty Acid	-0.106	1.19e-20
23	stearate (18:0)	HMDB0000827	Lipid	Long Chain Saturated Fatty Acid	-0.104	2.59e-20
24	pentadecanoate (15:0)	HMDB0000826	Lipid	Long Chain Saturated Fatty Acid	-0.102	1.80e-19
25	vanillylmandelate (VMA)	HMDB0000291	Amino Acid	Tyrosine Metabolism	-0.103	1.35e-18

	CHEMICAL NAME	HMDB	CHEMICAL CLASS	SUB PATHWAY	β	p_{bonf}
**26**	hexanoylglutamine	unknown	Lipid	Fatty Acid Metabolism (Acyl Glutamine)	-0.108	2.12e-18
**27**	urate	HMDB0000289	Nucleotide	Purine Metabolism, (Hypo)Xanthine/Inosine containing	-0.088	3.18e-18
**28**	3-hydroxydodecanedioate*	HMDB0000413	Lipid	Fatty Acid, Dicarboxylate	-0.108	3.19e-18
**29**	trans-4-hydroxyproline	HMDB0000725	Amino Acid	Urea cycle; Arginine and Proline Metabolism	0.104	1.09e-17
**30**	16-hydroxypalmitate	HMDB0006294	Lipid	Fatty Acid, Monohydroxy	-0.103	4.18e-17
**31**	hexanoylglycine	HMDB0000701	Lipid	Fatty Acid Metabolism (Acyl Glycine)	-0.104	8.99e-17
**32**	adrenate (22:4n6)	HMDB0002226	Lipid	Long Chain Polyunsaturated Fatty Acid (n3 and n6)	-0.096	4.32e-16
**33**	docosapentaenoate (n3 DPA; 22:5n3)	HMDB0006528	Lipid	Long Chain Polyunsaturated Fatty Acid (n3 and n6)	-0.093	4.89e-16
**34**	dodecenedioate (C12:1-DC)*	HMDB0000933	Lipid	Fatty Acid, Dicarboxylate	-0.101	5.00e-16
**35**	3-hydroxyhexanoate	HMDB0061652	Lipid	Fatty Acid, Monohydroxy	-0.101	5.58e-16
**36**	docosadienoate (22:2n6)	HMDB0061714	Lipid	Long Chain Polyunsaturated Fatty Acid (n3 and n6)	-0.092	6.59e-16
**37**	1,2-dilinoleoyl-GPC (18:2/18:2)	HMDB0008138	Lipid	Phosphatidylcholine (PC)	0.090	1.44e-15
**38**	2-hydroxybutyrate/2-hydroxyisobutyrate	HMDB0000729	Amino Acid	Glutathione Metabolism	-0.094	1.64e-15
**39**	1-linolenoyl-GPC (18:3)*	HMDB0010388	Lipid	Lysophospholipid	0.095	1.79e-14
**40**	1-(1-enyl-palmitoyl)-2-oleoyl-GPE (P-16:0/18:1)*	HMDB0011342	Lipid	Plasmalogen	0.094	2.00e-14
**41**	3-hydroxyisobutyrate	HMDB0000023	Amino Acid	Leucine, Isoleucine and Valine Metabolism	-0.094	5.40e-14
**42**	heptenedioate (C7:1-DC)*	unknown	Lipid	Fatty Acid, Dicarboxylate	-0.094	6.73e-14
**43**	(R)-3-hydroxybutyrylcarnitine	HMDB0013127	Lipid	Fatty Acid Metabolism (Acyl Carnitine, Hydroxy)	-0.094	9.43e-14
**44**	3-amino-2-piperidone	HMDB0000323	Amino Acid	Urea cycle; Arginine and Proline Metabolism	0.093	1.16e-13
**45**	1-linoleoyl-2-linolenoyl-GPC (18:2/18:3)*	HMDB0008141	Lipid	Phosphatidylcholine (PC)	0.088	1.57e-13
**46**	4-methyl-2-oxopentanoate	HMDB0000695	Amino Acid	Leucine, Isoleucine and Valine Metabolism	-0.077	1.68e-13
**47**	octadecanedioate (C18-DC)	HMDB0000782	Lipid	Fatty Acid, Dicarboxylate	-0.092	2.37e-13
**48**	alpha-ketobutyrate	HMDB0000005	Amino Acid	Methionine, Cysteine, SAM and Taurine Metabolism	-0.089	2.72e-13
**49**	3-hydroxyoctanoate	HMDB0001954	Lipid	Fatty Acid, Monohydroxy	-0.089	8.35e-13
**50**	5-dodecenoylcarnitine (C12:1)	HMDB13326	Lipid	Fatty Acid Metabolism (Acyl Carnitine, Monounsaturated)	-0.090	8.56e-13
**51**	N4-acetylcytidine	HMDB0005923	Nucleotide	Pyrimidine Metabolism, Cytidine containing	-0.091	8.89e-13
**52**	3-hydroxydecanoate	HMDB0002203	Lipid	Fatty Acid, Monohydroxy	-0.090	9.27e-13
**53**	3-hydroxysebacate	HMDB0000350	Lipid	Fatty Acid, Monohydroxy	-0.096	9.78e-13

	CHEMICAL NAME	HMDB	CHEMICAL CLASS	SUB PATHWAY	β	p_{bonf}
**54**	nonadecanoate (19:0)	HMDB0000772	Lipid	Long Chain Saturated Fatty Acid	-0.079	9.90e-13
**55**	3-hydroxylaurate	HMDB0000387	Lipid	Fatty Acid, Monohydroxy	-0.091	1.01e-12
**56**	1-stearoyl-2-linoleoyl-GPC (18:0/18:2)*	HMDB0008039	Lipid	Phosphatidylcholine (PC)	0.081	1.25e-12
**57**	dihomo-linolenate (20:3n3 or n6)	HMDB0002925	Lipid	Long Chain Polyunsaturated Fatty Acid (n3 and n6)	-0.083	2.46e-12
**58**	tetradecanedioate (C14-DC)	HMDB0000872	Lipid	Fatty Acid, Dicarboxylate	-0.089	3.71e-12
**59**	trans-2-hexenoylglycine	unknown	Lipid	Fatty Acid Metabolism (Acyl Glycine)	-0.086	4.24e-12
**60**	N-oleoyltaurine	unknown	Lipid	Endocannabinoid	-0.092	4.58e-12
**61**	myristoleoylcarnitine (C14:1)*	HMDB0240588	Lipid	Fatty Acid Metabolism (Acyl Carnitine, Monounsaturated)	-0.088	5.53e-12
**62**	3-hydroxymyristate	unknown	Lipid	Fatty Acid, Monohydroxy	-0.086	1.10e-11
**63**	arachidate (20:0)	HMDB0002212	Lipid	Long Chain Saturated Fatty Acid	-0.081	2.31e-11
**64**	xanthosine	HMDB0000299	Nucleotide	Purine Metabolism, (Hypo)Xanthine/Inosine containing	-0.089	3.09e-11
**65**	docosatrienoate (22:3n3)	HMDB0002823	Lipid	Long Chain Polyunsaturated Fatty Acid (n3 and n6)	-0.083	4.51e-11
**66**	ornithine	HMDB0000214	Amino Acid	Urea cycle; Arginine and Proline Metabolism	0.083	5.60e-11
**67**	(16 or 17)-methylstearate (a19:0 or i19:0)	HMDB0037397	Lipid	Fatty Acid, Branched	-0.076	6.31e-11
**68**	N-acetylglycine	HMDB0000532	Amino Acid	Glycine, Serine and Threonine Metabolism	-0.080	6.94e-11
**69**	citraconate/glutaconate	HMDB0000634	Energy	TCA Cycle	0.085	7.21e-11
**70**	taurocholenate sulfate*	unknown	Lipid	Secondary Bile Acid Metabolism	-0.084	8.76e-11
**71**	3-(4-hydroxyphenyl)lactate	HMDB0000755	Amino Acid	Tyrosine Metabolism	-0.077	1.01e-10
**72**	branched-chain, straight-chain, or cyclopropyl 12:1 fatty acid*	unknown	Partially Characterized Molecules	Partially Characterized Molecules	-0.083	1.10e-10
**73**	alpha-hydroxyisovalerate	HMDB0000407	Amino Acid	Leucine, Isoleucine and Valine Metabolism	-0.082	1.31e-10
**74**	1-(1-enyl-stearoyl)-2-linoleoyl-GPE (P-18:0/18:2)*	HMDB0011376	Lipid	Plasmalogen	0.078	1.41e-10
**75**	aconitate [cis or trans]	HMDB0000958	Energy	TCA Cycle	-0.078	2.56e-10
**76**	1-(1-enyl-palmitoyl)-2-linoleoyl-GPE (P-16:0/18:2)*	HMDB0011343	Lipid	Plasmalogen	0.081	3.02e-10
**77**	hyocholate	HMDB0000760	Lipid	Secondary Bile Acid Metabolism	0.081	7.15e-10
**78**	cortisone	HMDB0002802	Lipid	Corticosteroids	-0.075	7.38e-10
**79**	sphingomyelin (d18:1/18:1, d18:2/18:0)	HMDB0012101	Lipid	Sphingomyelins	-0.069	7.96e-10

	CHEMICAL NAME	HMDB	CHEMICAL CLASS	SUB PATHWAY	β	p_{bonf}
**80**	1-(1-enyl-stearoyl)-2-oleoyl-GPE (P-18:0/18:1)	HMDB0011375	Lipid	Plasmalogen	0.077	8.21e-10
**81**	1-stearoyl-GPC (18:0)	HMDB0010384	Lipid	Lysophospholipid	0.063	8.64e-10
**82**	hexadecadienoate (16:2n6)	HMDB0000477	Lipid	Long Chain Polyunsaturated Fatty Acid (n3 and n6)	-0.076	1.11e-09
**83**	dodecadienoate (12:2)*	unknown	Lipid	Fatty Acid, Dicarboxylate	-0.077	1.50e-09
**84**	1-(1-enyl-palmitoyl)-2-arachidonoyl-GPE (P-16:0/20:4)*	HMDB0011352	Lipid	Plasmalogen	0.076	2.50e-09
**85**	octadecenedioate (C18:1-DC)	unknown	Lipid	Fatty Acid, Dicarboxylate	-0.078	2.51e-09
**86**	myristoleate (14:1n5)	HMDB0002000	Lipid	Long Chain Monounsaturated Fatty Acid	-0.073	3.06e-09
**87**	(2 or 3)-decenoate (10:1n7 or n8)	unknown	Lipid	Medium Chain Fatty Acid	-0.078	3.79e-09
**88**	palmitoleoylcarnitine (C16:1)*	unknown	Lipid	Fatty Acid Metabolism (Acyl Carnitine, Monounsaturated)	-0.073	6.71e-09
**89**	andro steroid monosulfate C19H28O6S (1)*	HMDB0002759	Lipid	Androgenic Steroids	-0.075	9.97e-09
**90**	acisoga	HMDB0061384	Amino Acid	Polyamine Metabolism	-0.076	1.07e-08
**91**	succinoyltaurine	unknown	Amino Acid	Methionine, Cysteine, SAM and Taurine Metabolism	-0.077	1.35e-08
**92**	stearoyl sphingomyelin (d18:1/18:0)	HMDB0001348	Lipid	Sphingomyelins	-0.071	1.37e-08
**93**	5-dodecenoate (12:1n7)	HMDB0000529	Lipid	Medium Chain Fatty Acid	-0.073	1.58e-08
**94**	threonine	HMDB0000167	Amino Acid	Glycine, Serine and Threonine Metabolism	0.075	1.81e-08
**95**	malate	HMDB0031518	Energy	TCA Cycle	-0.074	2.64e-08
**96**	3-hydroxydecanoylcarnitine	HMDB0061636	Lipid	Fatty Acid Metabolism (Acyl Carnitine, Hydroxy)	-0.073	2.80e-08
**97**	3-methyl-2-oxovalerate	HMDB0000491	Amino Acid	Leucine, Isoleucine and Valine Metabolism	-0.063	2.84e-08
**98**	linolenate [alpha or gamma; (18:3n3 or 6)]	HMDB0003073	Lipid	Long Chain Polyunsaturated Fatty Acid (n3 and n6)	-0.073	2.88e-08
**99**	oleoyl ethanolamide	HMDB0002088	Lipid	Endocannabinoid	-0.068	4.01e-08
**100**	2-hydroxy-3-methylvalerate	HMDB0000317	Amino Acid	Leucine, Isoleucine and Valine Metabolism	-0.071	4.80e-08
**101**	erythronate*	HMDB0000613	Carbohydrate	Aminosugar Metabolism	-0.070	7.83e-08
**102**	oleoylcarnitine (C18:1)	HMDB0005065	Lipid	Fatty Acid Metabolism (Acyl Carnitine, Monounsaturated)	-0.070	1.06e-07
**103**	tetradecadienedioate (C14:2-DC)*	unknown	Lipid	Fatty Acid, Dicarboxylate	-0.072	1.48e-07
**104**	myristoylcarnitine (C14)	HMDB000506	Lipid	Fatty Acid Metabolism (Acyl Carnitine, Long Chain Saturated)	-0.071	1.62e-07
**105**	octadecenedioylcarnitine (C18:1-DC)*	unknown	Lipid	Fatty Acid Metabolism (Acyl Carnitine, Dicarboxylate)	-0.071	1.72e-07

	CHEMICAL NAME	HMDB	CHEMICAL CLASS	SUB PATHWAY	β	p_{bonf}
**106**	5-hydroxyhexanoate	HMDB0000409	Lipid	Fatty Acid, Monohydroxy	-0.075	2.11e-07
**107**	sphingomyelin (d18:0/18:0, d19:0/17:0)*	HMDB0012087	Lipid	Dihydrosphingomyelins	-0.063	2.23e-07
**108**	N-palmitoylglycine	HMDB0013034	Lipid	Fatty Acid Metabolism (Acyl Glycine)	-0.064	2.55e-07
**109**	arachidonate (20:4n6)	HMDB0001043	Lipid	Long Chain Polyunsaturated Fatty Acid (n3 and n6)	-0.065	2.75e-07
**110**	N-acetylthreonine	HMDB0062557	Amino Acid	Glycine, Serine and Threonine Metabolism	-0.068	3.19e-07
**111**	3-hydroxyadipate	HMDB0000345	Lipid	Fatty Acid, Dicarboxylate	-0.071	3.77e-07
**112**	phenyllactate (PLA)	HMDB0000779	Amino Acid	Phenylalanine Metabolism	-0.067	3.90e-07
**113**	5,6-dihydrothymine	HMDB0000079	Nucleotide	Pyrimidine Metabolism, Thymine containing	-0.070	5.58e-07
**114**	sebacate (C10-DC)	HMDB0000792	Lipid	Fatty Acid, Dicarboxylate	-0.070	6.94e-07
**115**	2-aminobutyrate	HMDB0000452	Amino Acid	Glutathione Metabolism	-0.068	7.09e-07
**116**	3-hydroxyoctanoylcarnitine (2)	unknown	Lipid	Fatty Acid Metabolism (Acyl Carnitine, Hydroxy)	-0.066	1.36e-06
**117**	1-myristoyl-2-arachidonoyl-GPC (14:0/20:4)*	HMDB0007883	Lipid	Phosphatidylcholine (PC)	0.064	1.40e-06
**118**	1-palmitoyl-2-docosahexaenoyl-GPC (16:0/22:6)	HMDB0007991	Lipid	Phosphatidylcholine (PC)	-0.062	1.82e-06
**119**	(S)-3-hydroxybutyrylcarnitine	HMDB0013127	Lipid	Fatty Acid Metabolism (Acyl Carnitine, Hydroxy)	-0.067	2.21e-06
**120**	1-(1-enyl-stearoyl)-2-arachidonoyl-GPE (P-18:0/20:4)*	HMDB0005779	Lipid	Plasmalogen	0.063	2.43e-06
**121**	docosahexaenoate (DHA; 22:6n3)	HMDB0002183	Lipid	Long Chain Polyunsaturated Fatty Acid (n3 and n6)	-0.062	3.70e-06
**122**	3-hydroxyoctanoylcarnitine (1)	unknown	Lipid	Fatty Acid Metabolism (Acyl Carnitine, Hydroxy)	-0.064	3.81e-06
**123**	xanthine	HMDB0000292	Nucleotide	Purine Metabolism, (Hypo)Xanthine/Inosine containing	-0.063	4.06e-06
**124**	cis-4-decenoylcarnitine (C10:1)	HMDB0013205	Lipid	Fatty Acid Metabolism (Acyl Carnitine, Monounsaturated)	-0.066	5.10e-06
**125**	3-hydroxyhexanoylcarnitine (1)	unknown	Lipid	Fatty Acid Metabolism (Acyl Carnitine, Hydroxy)	-0.065	5.36e-06
**126**	laurylcarnitine (C12)	HMDB000225	Lipid	Fatty Acid Metabolism (Acyl Carnitine, Medium Chain)	-0.064	6.95e-06
**127**	2-hydroxypalmitate	HMDB0031057	Lipid	Fatty Acid, Monohydroxy	-0.059	9.72e-06
**128**	adipoylcarnitine (C6-DC)	HMDB0061677	Lipid	Fatty Acid Metabolism (Acyl Carnitine, Dicarboxylate)	-0.064	1.13e-05
**129**	citramalate	HMDB0000426	Amino Acid	Glutamate Metabolism	-0.062	1.18e-05
**130**	myo-inositol	HMDB0000211	Lipid	Inositol Metabolism	-0.064	1.81e-05
**131**	N-acetylcarnosine	HMDB0012881	Amino Acid	Histidine Metabolism	-0.048	2.67e-05
**132**	N-acetylarginine	HMDB0004620	Amino Acid	Urea cycle; Arginine and Proline Metabolism	0.060	2.95e-05
**133**	glutarylcarnitine (C5-DC)	HMDB0013130	Amino Acid	Lysine Metabolism	-0.061	3.24e-05

	CHEMICAL NAME	HMDB	CHEMICAL CLASS	SUB PATHWAY	β	p_{bonf}
**134**	linoleoyl ethanolamide	HMDB0012252	Lipid	Endocannabinoid	-0.061	3.58e-05
**135**	linoleoylcholine*	HMDB0013213	Lipid	Fatty Acid Metabolism (Acyl Choline)	0.065	4.72e-05
**136**	cholate	HMDB0000619	Lipid	Primary Bile Acid Metabolism	0.062	5.03e-05
**137**	tridecenedioate (C13:1-DC)*	unknown	Lipid	Fatty Acid, Dicarboxylate	-0.061	5.08e-05
**138**	leucine	HMDB0000687	Amino Acid	Leucine, Isoleucine and Valine Metabolism	-0.049	9.38e-05
**139**	1,5-anhydroglucitol (1,5-AG)	HMDB0002712	Carbohydrate	Glycolysis, Gluconeogenesis, and Pyruvate Metabolism	0.056	9.39e-05
**140**	1-palmitoyl-2-stearoyl-GPC (16:0/18:0)	HMDB0007970	Lipid	Phosphatidylcholine (PC)	0.050	1.05e-04
**141**	androstenediol (3beta,17beta) disulfate (2)	HMDB0240313	Lipid	Androgenic Steroids	-0.053	1.16e-04
**142**	1-myristoyl-2-palmitoyl-GPC (14:0/16:0)	HMDB0007869	Lipid	Phosphatidylcholine (PC)	0.058	1.26e-04
**143**	1-linoleoyl-GPC (18:2)	HMDB0010386	Lipid	Lysophospholipid	0.052	1.53e-04
**144**	pregnenetriol disulfate*	unknown	Lipid	Pregnenolone Steroids	-0.053	1.62e-04
**145**	N-lactoyl leucine	HMDB0062176	Amino Acid	Leucine, Isoleucine and Valine Metabolism	-0.053	1.64e-04
**146**	3-methyl-2-oxobutyrate	HMDB0000019	Amino Acid	Leucine, Isoleucine and Valine Metabolism	-0.054	1.82e-04
**147**	tetradecadienoate (14:2)*	HMDB0000560	Lipid	Long Chain Polyunsaturated Fatty Acid (n3 and n6)	-0.059	1.88e-04
**148**	hexanoylcarnitine (C6)	HMDB0000756	Lipid	Fatty Acid Metabolism (Acyl Carnitine, Medium Chain)	-0.059	2.00e-04
**149**	1-(1-enyl-palmitoyl)-2-linoleoyl-GPC (P-16:0/18:2)*	HMDB0011211	Lipid	Plasmalogen	0.051	2.12e-04
**150**	N-acetyl-aspartyl-glutamate (NAAG)	HMDB0001067	Amino Acid	Glutamate Metabolism	-0.057	2.39e-04
**151**	dodecanedioate (C12-DC)	HMDB0000623	Lipid	Fatty Acid, Dicarboxylate	-0.058	2.71e-04
**152**	N-acetylglucosamine/N-acetylgalactosamine	HMDB0000212	Carbohydrate	Aminosugar Metabolism	-0.055	2.90e-04
**153**	docosatrienoate (22:3n6)*	unknown	Lipid	Long Chain Polyunsaturated Fatty Acid (n3 and n6)	-0.060	2.96e-04
**154**	S-1-pyrroline-5-carboxylate	HMDB0001301	Amino Acid	Glutamate Metabolism	0.056	3.10e-04
**155**	N-stearoyl-sphingosine (d18:1/18:0)*	HMDB0004950	Lipid	Ceramides	-0.049	3.65e-04
**156**	3-hydroxybutyrate (BHBA)	HMDB0000442	Lipid	Ketone Bodies	-0.058	3.95e-04
**157**	beta-citrylglutamate	unknown	Amino Acid	Glutamate Metabolism	-0.056	4.06e-04
**158**	pregnenediol disulfate (C21H34O8S2)*	unknown	Lipid	Pregnenolone Steroids	-0.052	4.13e-04
**159**	fumarate	HMDB0000134	Energy	TCA Cycle	-0.057	4.16e-04
**160**	13-HODE + 9-HODE	HMDB0004670	Lipid	Fatty Acid, Monohydroxy	-0.055	4.37e-04
**161**	glutamine_degradant*	unknown	Partially Characterized Molecules	Partially Characterized Molecules	-0.046	5.19e-04

	CHEMICAL NAME	HMDB	CHEMICAL CLASS	SUB PATHWAY	β	p_{bonf}
**162**	glutamine conjugate of C6H10O2 (2)*	unknown	Partially Characterized Molecules	Partially Characterized Molecules	-0.056	5.47e-04
**163**	eicosanedioate (C20-DC)	unknown	Lipid	Fatty Acid, Dicarboxylate	0.056	5.58e-04
**164**	5,6-dihydrouridine	HMDB0000497	Nucleotide	Pyrimidine Metabolism, Uracil containing	-0.055	6.18e-04
**165**	2R,3R-dihydroxybutyrate	HMDB0000498	Lipid	Fatty Acid, Dihydroxy	0.055	6.27e-04
**166**	3-hydroxy-3-methylglutarate	HMDB0000355	Lipid	Mevalonate Metabolism	-0.053	6.56e-04
**167**	cholic acid glucuronide	HMDB0002577	Lipid	Primary Bile Acid Metabolism	0.059	7.37e-04
**168**	androstenediol (3beta,17beta) monosulfate (1)	HMDB0240429	Lipid	Androgenic Steroids	-0.051	8.08e-04
**169**	androstenediol (3beta,17beta) disulfate (1)	HMDB0240313	Lipid	Androgenic Steroids	-0.053	9.49e-04
**170**	deoxycholic acid glucuronide	unknown	Lipid	Secondary Bile Acid Metabolism	0.055	9.83e-04
**171**	pimeloylcarnitine/3-methyladipoylcarnitine (C7-DC)	unknown	Lipid	Fatty Acid Metabolism (Acyl Carnitine, Dicarboxylate)	-0.052	1.16e-03
**172**	hexadecenedioate (C16:1-DC)*	unknown	Lipid	Fatty Acid, Dicarboxylate	-0.055	1.29e-03
**173**	1-palmitoleoyl-2-linolenoyl-GPC (16:1/18:3)*	HMDB0008008	Lipid	Phosphatidylcholine (PC)	0.050	1.34e-03
**174**	palmitoylcarnitine (C16)	HMDB0000222	Lipid	Fatty Acid Metabolism (Acyl Carnitine, Long Chain Saturated)	-0.049	1.37e-03
**175**	N6-carbamoylthreonyladenosine	HMDB0041623	Nucleotide	Purine Metabolism, Adenine containing	-0.051	1.49e-03
**176**	3-(3-amino-3-carboxypropyl)uridine*	unknown	Nucleotide	Pyrimidine Metabolism, Uracil containing	-0.052	1.59e-03
**177**	epiandrosterone sulfate	HMDB0062657	Lipid	Androgenic Steroids	-0.050	1.65e-03
**178**	C-glycosyltryptophan	HMDB0240296	Amino Acid	Tryptophan Metabolism	-0.052	1.66e-03
**179**	2,3-dihydroxy-2-methylbutyrate	HMDB0029576	Amino Acid	Leucine, Isoleucine and Valine Metabolism	-0.053	1.69e-03
**180**	5alpha-androstan-3beta,17beta-diol monosulfate (2)	unknown	Lipid	Androgenic Steroids	-0.050	1.74e-03
**181**	(N(1) + N(8))-acetylspermidine	HMDB0002189	Amino Acid	Polyamine Metabolism	-0.054	2.12e-03
**182**	N-lactoyl phenylalanine	HMDB0062175	Amino Acid	Phenylalanine Metabolism	-0.049	2.21e-03
**183**	4-hydroxyphenylacetoylcarnitine	unknown	Amino Acid	Tyrosine Metabolism	-0.052	2.50e-03
**184**	alpha-ketoglutaramate*	HMDB0001552	Amino Acid	Glutamate Metabolism	-0.050	2.81e-03
**185**	1-oleoyl-GPC (18:1)	HMDB0002815	Lipid	Lysophospholipid	0.047	3.13e-03
**186**	palmitoylcholine	HMDB0240592	Lipid	Fatty Acid Metabolism (Acyl Choline)	0.052	3.14e-03
**187**	taurolithocholate 3-sulfate	HMDB0002580	Lipid	Secondary Bile Acid Metabolism	-0.052	3.34e-03
**188**	10-undecenoate (11:1n1)	HMDB0033724	Lipid	Medium Chain Fatty Acid	-0.052	3.37e-03
**189**	cytosine	HMDB0000630	Nucleotide	Pyrimidine Metabolism, Cytidine containing	0.051	3.52e-03
**190**	16a-hydroxy DHEA 3-sulfate	HMDB0062544	Lipid	Androgenic Steroids	-0.051	3.71e-03

	CHEMICAL NAME	HMDB	CHEMICAL CLASS	SUB PATHWAY	β	p_{bonf}
**191**	glycine conjugate of C10H14O2 (1)*	unknown	Partially Characterized Molecules	Partially Characterized Molecules	-0.051	4.04e-03
**192**	N-oleoylserine	unknown	Lipid	Endocannabinoid	-0.051	4.26e-03
**193**	1-(1-enyl-palmitoyl)-GPC (P-16:0)*	HMDB0010407	Lipid	Lysoplasmalogen	0.046	4.32e-03
**194**	phosphate	HMDB0001429	Energy	Oxidative Phosphorylation	-0.047	4.34e-03
**195**	phenylalanine	HMDB0000159	Amino Acid	Phenylalanine Metabolism	-0.050	4.43e-03
**196**	indoleacetate	HMDB0000197	Amino Acid	Tryptophan Metabolism	-0.052	4.66e-03
**197**	21-hydroxypregnenolone disulfate	unknown	Lipid	Pregnenolone Steroids	-0.048	5.46e-03
**198**	GlcNAc sulfate conjugate of C21H34O2 steroid**	unknown	Partially Characterized Molecules	Partially Characterized Molecules	-0.054	6.58e-03
**199**	sphingomyelin (d18:1/17:0, d17:1/18:0, d19:1/16:0)	unknown	Lipid	Sphingomyelins	-0.044	6.88e-03
**200**	imidazole lactate	HMDB0002320	Amino Acid	Histidine Metabolism	-0.050	6.99e-03
**201**	N-lactoyl tyrosine	HMDB0062177	Amino Acid	Tyrosine Metabolism	-0.046	8.33e-03
**202**	cortisol	HMDB0000063	Lipid	Corticosteroids	-0.049	9.12e-03
**203**	beta-hydroxyisovalerate	HMDB0000754	Amino Acid	Leucine, Isoleucine and Valine Metabolism	-0.047	9.35e-03
**204**	N-acetylserine	HMDB0002931	Amino Acid	Glycine, Serine and Threonine Metabolism	-0.049	1.04e-02
**205**	linoleoylcarnitine (C18:2)*	HMDB0006469	Lipid	Fatty Acid Metabolism (Acyl Carnitine, Polyunsaturated)	-0.044	1.15e-02
**206**	maleate	HMDB0000176	Lipid	Fatty Acid, Dicarboxylate	0.049	1.20e-02
**207**	mannitol/sorbitol	HMDB0000247	Carbohydrate	Fructose, Mannose and Galactose Metabolism	0.049	1.52e-02
**208**	7-alpha-hydroxy-3-oxo-4-cholestenoate (7-Hoca)	HMDB0012458	Lipid	Sterol	-0.047	1.62e-02
**209**	malonylcarnitine	HMDB0002095	Lipid	Fatty Acid Synthesis	-0.052	1.64e-02
**210**	4-hydroxyphenylacetate	HMDB0000020	Amino Acid	Phenylalanine Metabolism	-0.049	1.65e-02
**211**	chenodeoxycholate	HMDB0000518	Lipid	Primary Bile Acid Metabolism	0.049	1.69e-02
**212**	dehydroepiandrosterone sulfate (DHEA-S)	HMDB0001032	Lipid	Androgenic Steroids	-0.044	1.74e-02
**213**	biliverdin	HMDB0001008	Cofactors and Vitamins	Hemoglobin and Porphyrin Metabolism	-0.046	1.77e-02
**214**	isoleucine	HMDB0000172	Amino Acid	Leucine, Isoleucine and Valine Metabolism	-0.039	1.81e-02
**215**	gamma-glutamylleucine	HMDB0011171	Peptide	Gamma-glutamyl Amino Acid	-0.040	1.89e-02
**216**	homocitrulline	HMDB0000679	Amino Acid	Urea cycle; Arginine and Proline Metabolism	-0.048	1.95e-02
**217**	indolelactate	HMDB0000671	Amino Acid	Tryptophan Metabolism	-0.044	2.07e-02
**218**	threonate	HMDB0062620	Cofactors and Vitamins	Ascorbate and Aldarate Metabolism	-0.046	2.12e-02

	CHEMICAL NAME	HMDB	CHEMICAL CLASS	SUB PATHWAY	β	p_{bonf}
**219**	guanidinoacetate	HMDB0000128	Amino Acid	Creatine Metabolism	-0.045	2.36e-02
**220**	gamma-glutamylphenylalanine	HMDB0000594	Peptide	Gamma-glutamyl Amino Acid	-0.043	2.49e-02
**221**	2-butenoylglycine	unknown	Lipid	Fatty Acid Metabolism (Acyl Glycine)	-0.045	2.78e-02
**222**	citrate	HMDB0000094	Energy	TCA Cycle	-0.045	2.83e-02
**223**	5alpha-androstan-3beta,17beta-diol disulfate	HMDB00493	Lipid	Androgenic Steroids	-0.042	2.93e-02
**224**	pseudouridine	HMDB0000767	Nucleotide	Pyrimidine Metabolism, Uracil containing	-0.046	3.00e-02
**225**	3beta-hydroxy-5-cholestenoate	unknown	Lipid	Sterol	-0.041	3.14e-02
**226**	lactosyl-N-palmitoyl-sphingosine (d18:1/16:0)	unknown	Lipid	Lactosylceramides (LCER)	-0.044	3.86e-02
**227**	N-formylmethionine	HMDB0001015	Amino Acid	Methionine, Cysteine, SAM and Taurine Metabolism	-0.046	3.99e-02
**228**	bilirubin (Z,Z)	HMDB0000054	Cofactors and Vitamins	Hemoglobin and Porphyrin Metabolism	-0.042	4.42e-02
**229**	5alpha-androstan-3alpha,17beta-diol disulfate	HMDB0094682	Lipid	Androgenic Steroids	-0.039	4.90e-02
**230**	methionine	HMDB0000696	Amino Acid	Methionine, Cysteine, SAM and Taurine Metabolism	-0.043	4.92e-02
