# Supplementary material for: The metabolic signature of salt intake: a cross-sectional analysis from the SCAPIS-study
Source: Nutr Metab (Lond). 2025 Sep 2;22:104. doi: 10.1186/s12986-025-00997-y (PMC12406461; doi:10.1186/s12986-025-00997-y)

Additional file 1

1. **Distribution of est24hNa**

2. **Missing data**
   a. Missing data for included metabolites
   b. Missing data for covariates

3. **Restricted cubic splines and ANOVA analyses of the eight CC**
   a. Amino acids
   b. Carbohydrates
   c. Cofactors and Vitamins
   d. Energy
   e. Lipids
   f. Nucleotides
   g. Peptides
   h. Partially characterized molecules

4. **Minimal adjusted restricted cubic splines for selected covariates**
   a. Systolic blood pressure
   b. Diastolic blood pressure
   c. BMI
   d. Cholesterol

5. **Restricted cubic splines and ANOVA analysis for the add-on analysis of piperine and proline-betaine**
   a. Piperine
   b. Proline-Betaine

1. Distribution of est24hNa

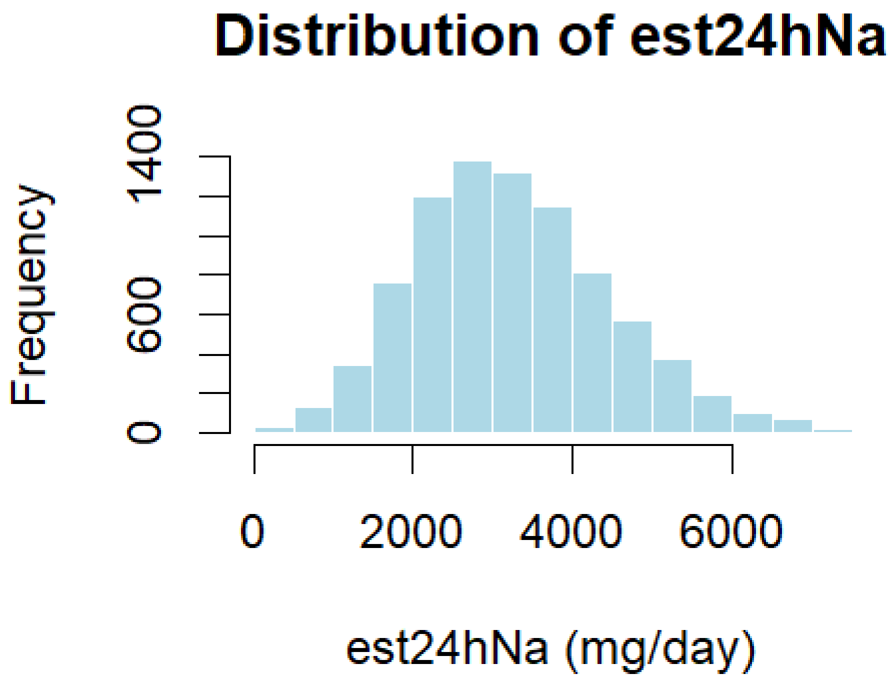

2a.

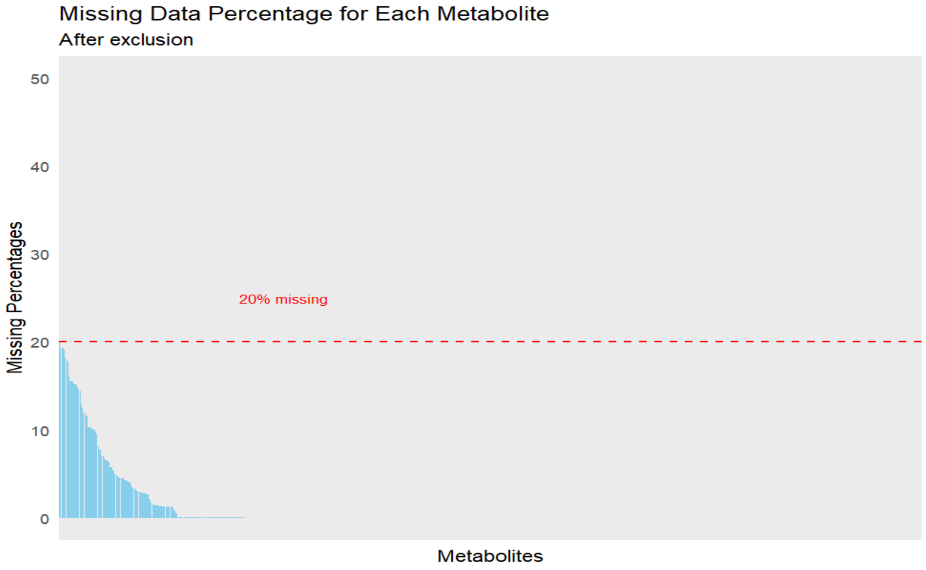

2b. Missing data for covariates

Variable	% missing
**Systolic blood pressure**	0.6
**Diastolic blood pressure**	0.6
**Sex**	0
**Age**	0
**BMI**	0
**Smoking status**	3.9
**Diabetes Mellitus**	3.9
**Cholesterol**	0.1
**eGFR**	0.1
**Hypertension medication**	2.8
**Lipid medication**	2.8
**Energy intake**	1.5

3a. Amino Acids

ANOVA PC1 amino acids vs est24hNa

Analysis of Variance Response: PC1aa

**Factor**	**p**
**est24hNa**	0.1364
**Nonlinear**	0.0628

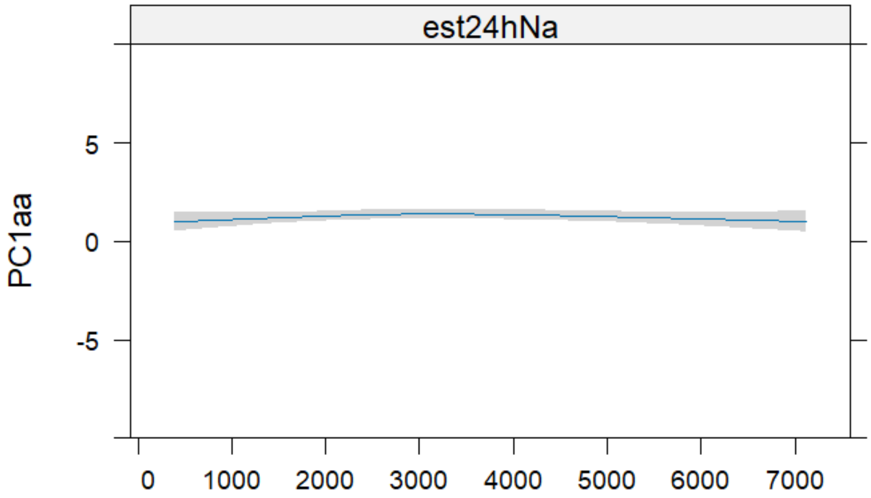

3b. Carbohydrates

ANOVA of PC1 carbohydrates vs est24hNa

Analysis of Variance Response: PC1carbohydrates

**Factor**	**p**
**est24hNa**	0.0733
**Nonlinear**	0.0348

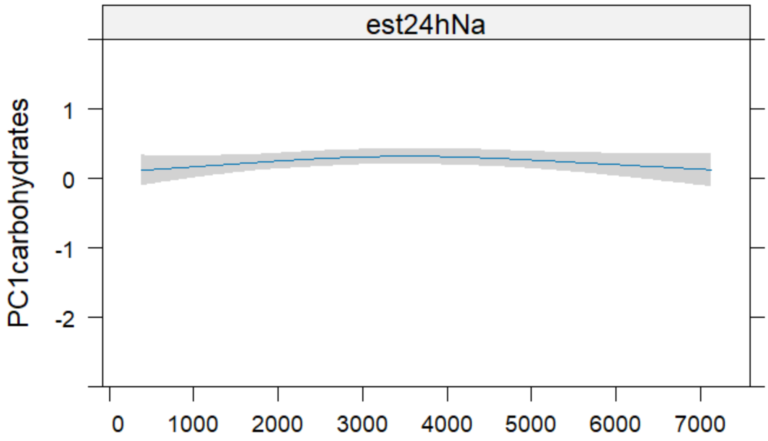

3c. Cofactors and Vitamins

ANOVA of PC1 cofactors and vitamins vs est24hNa

Analysis of Variance   Response: PC1cv

Factor	P
est24hNa	0.3626
Nonlinear	0.9798

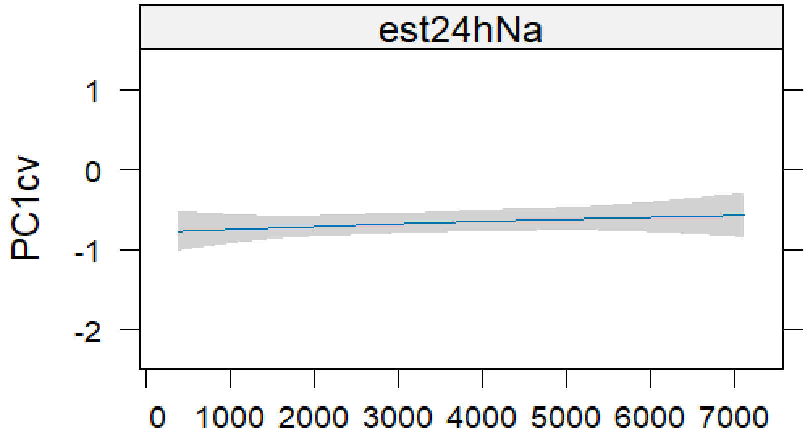

3d. Energy

ANOVA of PC1 energy vs est24hNa

Analysis of Variance   Response: PC1energy

Factor	p
est24hNa	<.0001
Nonlinear	<.0001

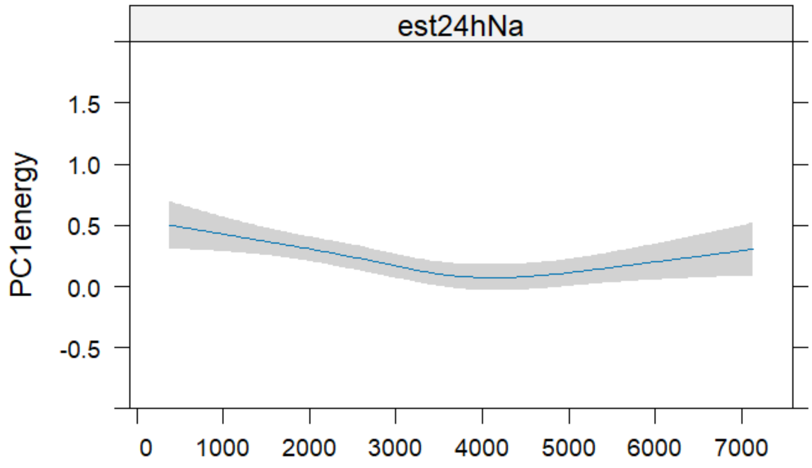

3e. Lipids

ANOVA of PC1 lipids vs est24hNa

Analysis of Variance Response: PC1lipids

Factor	P
est24hNa	<.0001
Nonlinear	<.0001

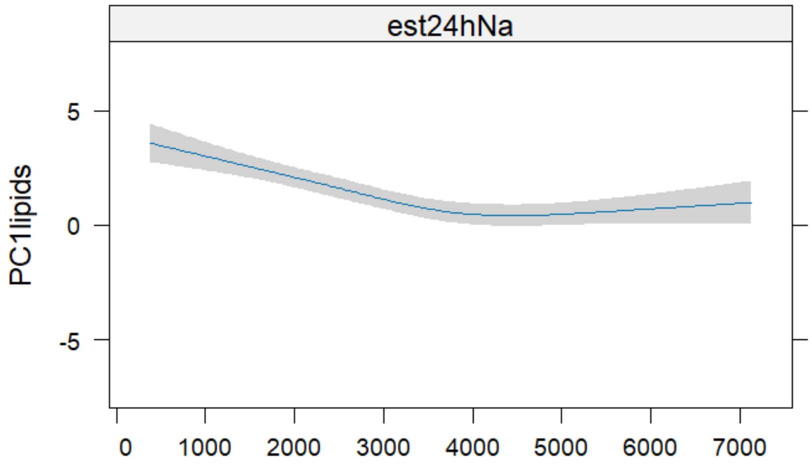

3f. Nucleotides

ANOVA of PC1 nucleotides vs est24hNa

Analysis of Variance Response: PC1nucleotides

Factor	P
est24hNa	0.0329
Nonlinear	0.0139

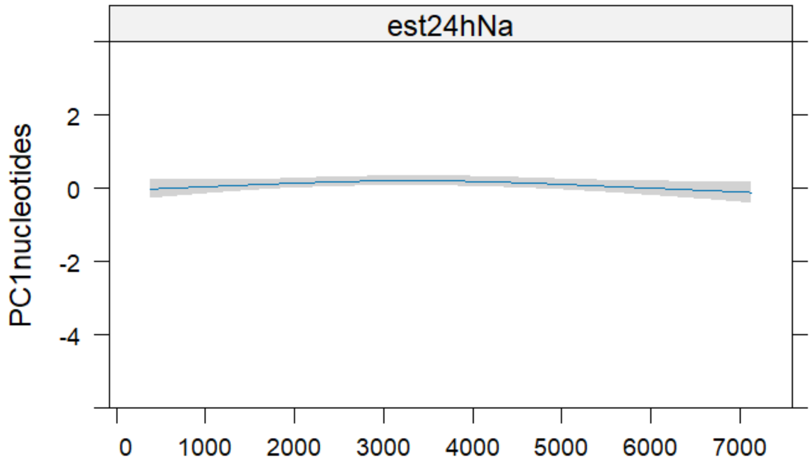

3g. Peptides

ANOVA of PC1 peptides vs est24hNa

Analysis of Variance   Response: PC1peptides

Factor	P
est24hNa	0.1958
Nonlinear	0.1081

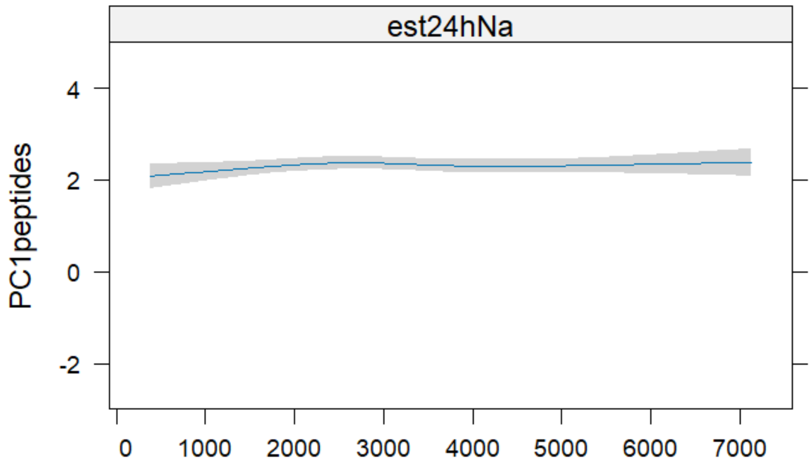

3h. Partially characterized molecules

ANOVA of PC1 partially characterized molecules vs est24hNa

Analysis of Variance   Response: PC1pcm

Factor	P
est24hNa	0.0007
Nonlinear	0.4768

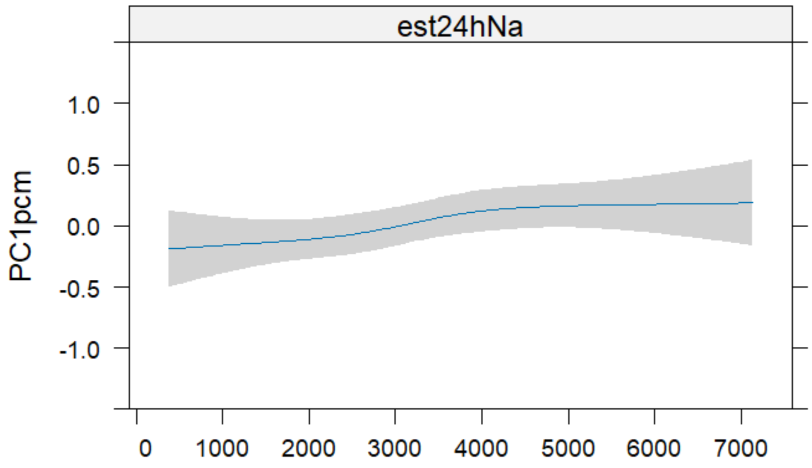

Restricted cubic splines for selected covariates

4a. *Systolic blood pressure (sex and age adjusted).*

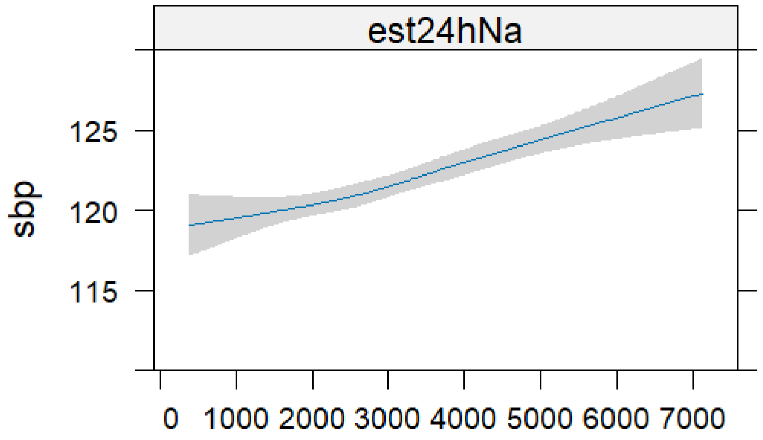

4b. *Diastolic blood pressure (sex and age adjusted).*

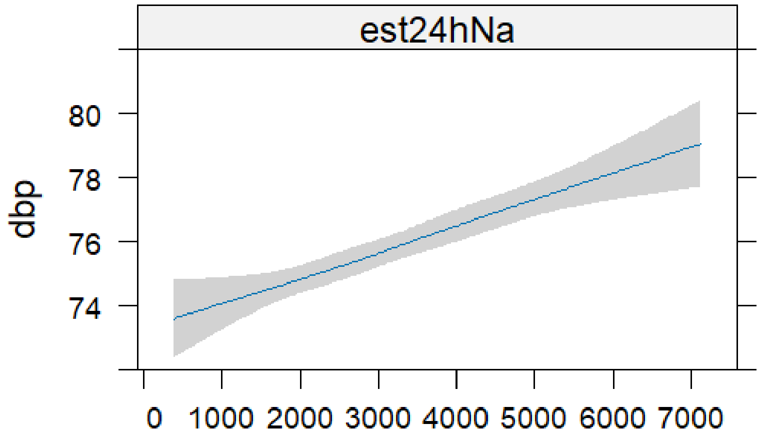

4c. *BMI (sex, age and calorie intake adjusted)*

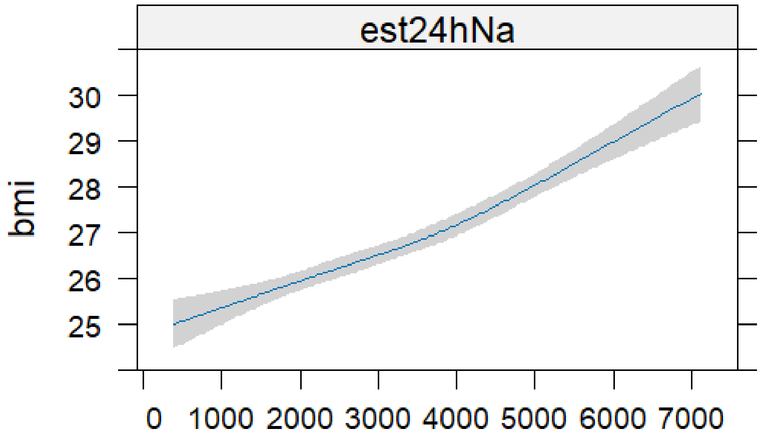

4d. *Cholesterol (sex and age adjusted)*

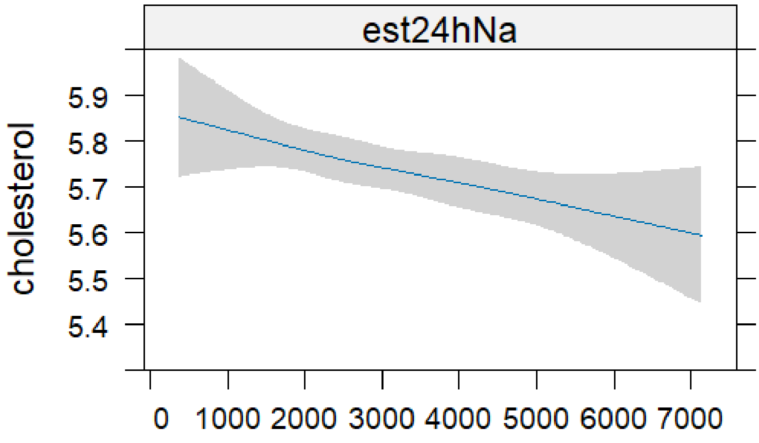

Add-on analysis for Piperine and Proline-Betaine

5a. *Restricted cubic spline for Piperine (highy adjusted). ANOVA* $p < 0.0001$, $p_{non\text{-}linear} = 0.49$

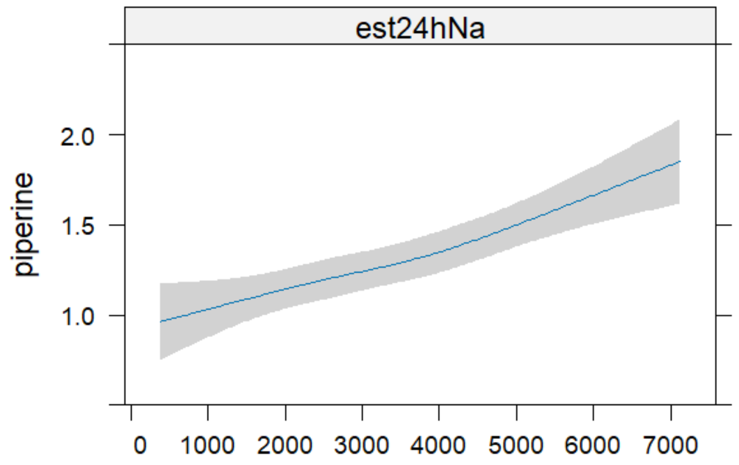

5b. *Proline-Betaine (highly adjusted). Anova:* $p < 0.0001$, $p_{non\text{-}linear} = 0.83$

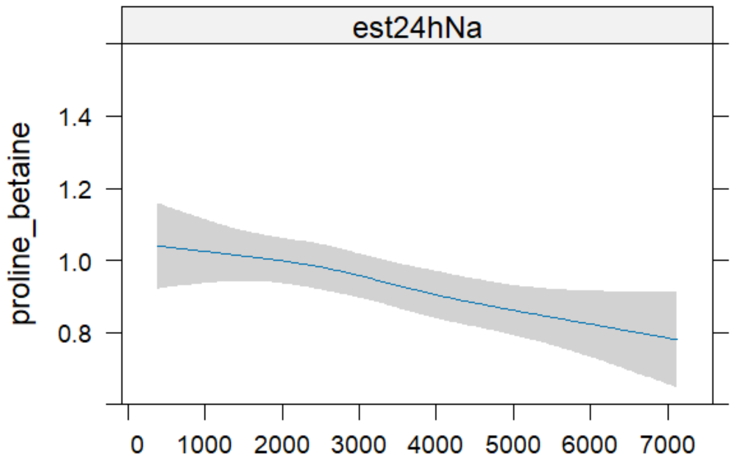

Supplement: Supplementary file 4 — Supplementary Material 4 [file 12986_2025_997_MOESM4_ESM.pdf]
